# Intervention through Short Messaging System (SMS) and phone call alerts reduced HbA1C levels in ~47% type-2 diabetics–results of a pilot study

**DOI:** 10.1371/journal.pone.0241830

**Published:** 2020-11-17

**Authors:** Kanakavalli K. Kundury, Basavanagowdappa Hathur

**Affiliations:** 1 Department of Health System Management Studies, JSS Academy of Higher Education & Research (JSS AHER), Mysore, Karnataka, India; 2 Special Interest Group in Patient Care Management (SIG-PCM), Mysore, Karnataka, India; 3 Department of Medicine, JSS Medical College & Hospital, Mysore, Karnataka, India; 4 JSS Medical College, Mysore, Karnataka, India; 5 Special Interest Group in Patient Care Management (SIG-PCM), JSS AHER, Mysore, Karnataka, India; Universidad Miguel Hernandez de Elche, SPAIN

## Abstract

**Purpose:**

Despite extensive research and newer methods of interventions, the incidence and prevalence of diabetes is increasing at an alarming rate. Currently, according to world health organization (WHO) statistics, 422 million individuals are suffering from diabetes worldwide. In India, recent estimates have reported a significant increase in the number of diabetics in the last decade. Poor dietary habits, minimal adherence to treatment regimens, lack of timely education are some of the contributing factors for increasing incidence and huge economic burden; which can be handled by life style behavior modifications backed up by hand holding through continuous education. Prior studies have demonstrated the efficacy of various self-management tools and educational programs in better disease management behaviors among individuals with diabetes. Among various self-management tools, educating the individuals and alerting them using mobile phone calls and short messaging system (SMS) are widely accepted due to (a) the increasing mobile phone users and (b) availability of short messaging systems in local languages in the recent years. Therefore, a pilot study was conducted to determine the benefit of educating patients through phone calls and SMS in the self-management of diabetes.

**Objectives:**

The objective of the study is to determine the feasibility and utility of SMS and phone call-based interventions in the management of diabetes by comparing the HbA1c values.

**Methodology:**

The study was conducted for a period of 14 months from December 2017 till Feb 2019. Out of 380 individuals initially enrolled into the study, 120 had completed the 14-months period. Diabetes education through SMS and phone calls was provided on regular basis, and HbA1C levels at baseline, 8-months and 14-months quantified. In addition, feedback on patients’ satisfaction and utility of the SMS / Phone calls was collected using questionnaires.

**Results:**

Data from our study demonstrated that after 8-months of intervention through phone calls, a significant increase in the number of individuals with HbA1c in the range of 5.1 to 7.0 was observed (from 27 individuals at base line to 37 individuals after 8-months intervention). Much more significant improvement in the number of individuals with lower HbA1c was observed at 14-months of intervention, indicating the benefit of regular phone call-based system in managing diabetes. A Chi square (χ^2^) test was performed to examine if the frequencies in the cells varied at baseline and at 8 and 14 months.

**Conclusion:**

Chronic diseases like diabetes needs awareness and education to patients in adopting disease self-management practices. As mobile phone users are increasing in number, providing diabetes management education through mobile phone intervention could be a viable strategy for controlling diabetes.

## Introduction

Diabetes and associated disorders are the major contributors for increasing morbidity, mortality and economic loss due to non-communicable disease across the globe [1–3]. According to World Health Organization statistics, the number of individuals suffering from diabetes has increased from 108 million in 1980 to 422 million in 2014 (ie., ~10 million individuals every year) [3, 4]. This number might in fact be more as a recent report by International Diabetes Federation (IDF) pointed that ~50% of individuals with diabetes are undiagnosed, especially in developing and under developed countries [5]. In India, according to IDF statistics, the total number of individuals with diabetes were ~73 million in the year 2017. While prevention of diabetes is critical to reduce the number of diabetic cases; developing strategies for managing and reducing the complications of diabetes are utmost important to control the effected population. Hence, better strategies are urgently required to educate affected individuals about diabetes, the signs and symptoms, its complications, treatment strategies including dietary habits, exercise and life style behavior modifications [6–8]. However, availability and reachability of diabetes education is a challenging task as majority of individuals are either un-educated or living in remote areas where accessibility to healthcare is very minimal. Therefore, a viable and cost-effective method is required to educate diabetes patients.

India is one of the fast-developing countries with majority of population using mobile phones with 3G and 4G networks. According to Telecom Regulatory Authority of India (TRAI), more than 90% of individuals are using mobile networks (ie., >1,176 million mobile users out of 1298 million total population). Among these mobile customers ~647 million are living in urban areas while the remaining 529 million are in rural areas [9]. Therefore, use of mobile phone-based education programs are likely to reach more than 90% of population in India. Earlier research has demonstrated the usage of SMS based health education programs for the management of chronic diseases and proven effective [10, 11]. However, it is currently unknown which method of mobile phone mediated communication (Short Messaging System (SMS) or Phone Calls) is effective in educating patients and providing options for better management of disease. Hence, behavioral theories such as Health Belief Model and Levanthal’s Common Sense Model of Self-Regulation were referred to understand various elements to be considered while providing health education through mobile phones.

A pilot study was conducted in individuals with diabetes using SMS and phone calls and assessed the feasibility by measuring the changes in HbA1c level before and after phone call mediated intervention. Results of our study showed a decrease of HbA1c in ~47% individuals due to mobile phone call and SMS mediated health education intervention. In conclusion, results of our study identified mobile phone calls could be an efficient way for self-management of diabetes.

## Materials and methods

The study was retrospectively registered in ISRCTN registry approved by WHO (ISRCTN12528490 https://doi.org/10.1186/ISRCTN12528490).

### Study site

The pilot study was conducted for a period of 14 months from December 2017 till Feb 2019. The study was approved by Institutional Ethics Committee of JSS Medical College, JSS Academy of Higher Education & Research (Protocol #: JSSMC/11/5976/2016-17; Dated: 30.11.2016; S1 & S2 Files). IEC of JSS AHER, an ISO recognized committee, operates under the guidelines of Indian Council of Medical Research (ICMR), Govt of India. The study was conducted at JSS Hospital, a 1800-bedded tertiary care teaching hospital, located in the Mysuru district of Karnataka, India.

### Recruitment of study participants

Participants in the current study were recruited for a period of 3 months from September-December 2017 using convenient sampling method. Patients with confirmed Type2 diabetes visiting General Medicine Department of JSS hospital and meeting the inclusion criteria were enrolled into the study. Informed consent was taken from the participants enrolled in to the study (n = 380).

Patients with confirmed Type-2 diabetes were tested for capillary blood glucose (CBG) in the Nutrition and Dietetics department; and those who provided a positive consent were considered for the study. Further details such as age, gender, duration of diabetes, postal address and contact number of the respective participant were collected; and a tentative date of study commencement and study orientation program were intimated.

### Research site

Study commenced with an orientation program on diabetes care management. A self-structured questionnaire was used to record demographic information of study participants as well as to identify diabetes self-management practices (S3 File). A diabetologist along with a certified dietician provided diabetes care management education using a Power Point Presentation (PPT) (S4 & S5 Files). After interaction and doubt clarification, 2.5mL blood was collected for HbA1C investigations from each participant. Samples were processed in the clinical laboratory of department of Bio-chemistry using D-10 Hemoglobin Testing System from Bio-Rad (Bio-Rad Laboratories India Pvt Ltd, Gurugram, Haryana, India), which separates HbA1C based on the Ion Exchange Chromatography (IEC) principle.

### Criteria for inclusion and exclusion

**Fig 1:**
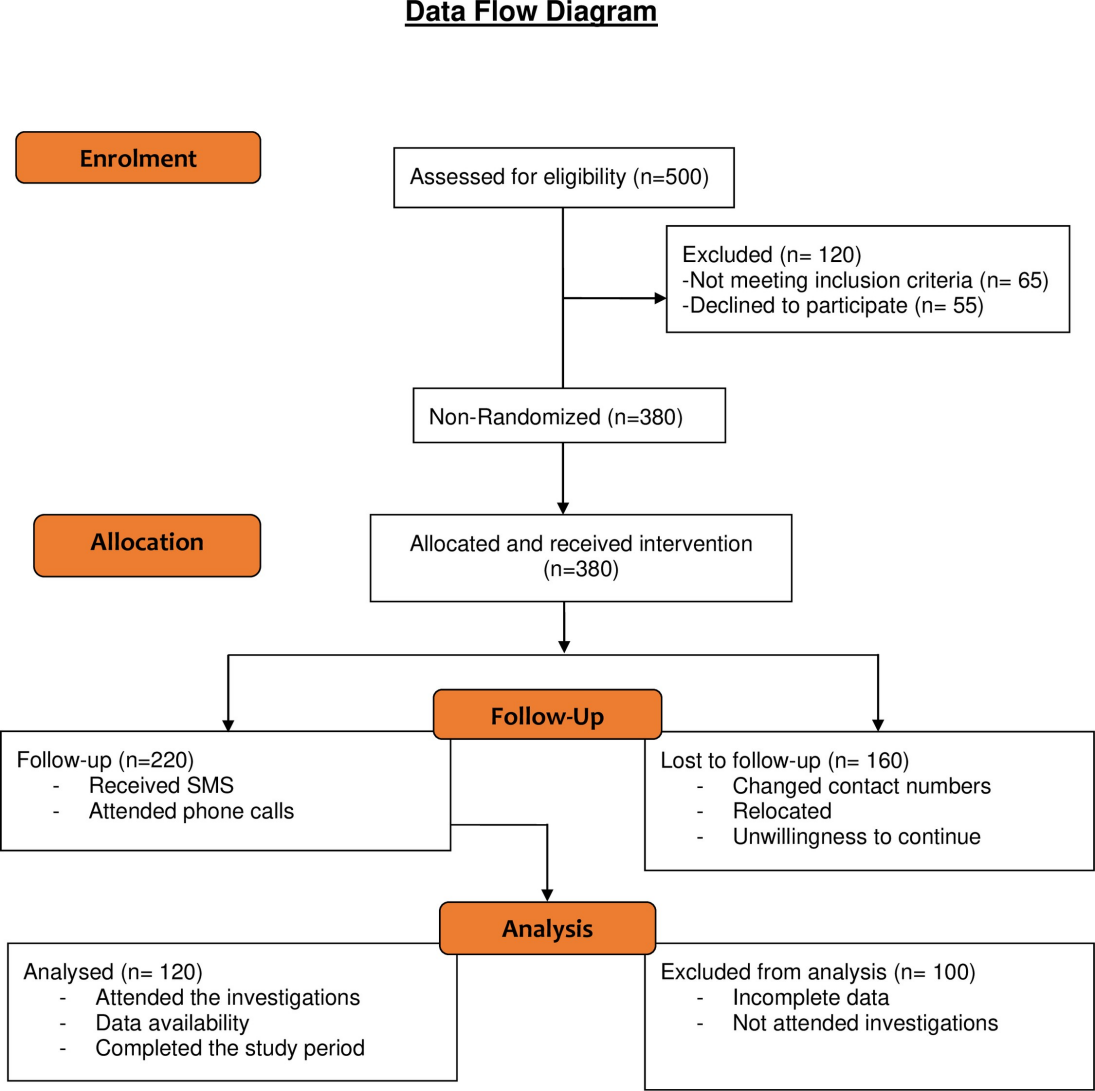
Consort flowchart. The flow chart describing the participant recruitment and follow-up procedures into the current study.

The inclusion criteria were: (a) Individuals with confirmed Type2 diabetes (b) no critical illness such as Cardio Vascular Diseases (CVDs), Cancer, and AIDS; (c) ability to use mobile phones and communicate in local language Kannada or in English; (d) ability to provide a consent to participate in the study. Information on informed consent was explained to each study participant in local language as well as in English (S6 & S7 Files) and signatures obtained confirming their participation into the study. Individuals with critical illness and/or no mobile phone access were excluded from the study. Although the study initiated with 380 individuals, only 120 participants have completed the 14-months duration. Hence, the final data pertaining to HbA1c variations was computed with 120 subjects (Fig 1).

### Study method

An orientation class was arranged and study commenced with a lecture on diabetes self-management education by a diabetologist and a certified dietician through a Power Point Presentation to the participants. Information related to diabetes and associated co-morbidities, dietary practices, physical activity, medication adherence, periodic doctor visits and life style modifications required for diabetes self-management was provided to the study participants in local language (S4 & S5 Files). Participants were also provided a handout containing information related to diabetes along with a tabular form to record their blood sugar values (S8 & S9 Files). Subsequently, diabetes education through SMS and phone calls was provided once in a week in local language ie., Kannada, and English (S10 File). In addition to the diabetes educational messages, wishes for festivals and any special events; information on orientation / re-orientation camps were also sent to study participants for better patient engagement. A total of 6 diabetes educational messages were composed and sent to the study participants on rotational basis every week. An SMS Log Sheet was created to record the delivery status of SMS (S11 File). Considering the feedback given by study participants at 8 months (preferring phone calls over SMS), only weekly phone calls were made for the extended study period of 14 months (S12 & S13 Files). Script for phone call-based education was provided in English and in local language Kannada (S14 & S15 Files). Delivery of diabetes educational content was further confirmed by a separate call-log sheet (S16 File). Data capture form was used to capture the participants’ diabetes management practices (S17 File).

Diabetes knowledge assessment and participants feedback on study was obtained using questionnaires (S12 File). All the records pertaining to this study were maintained in an iron safe with a lock and key, thus making them accessible only to the principal investigators of this study. Re-orientation class was arranged at every 4^th^ month to assess the participants’ knowledge on diabetes management. Any changes in the prescribed medicines/dosing schedule/dose were noted for individual patient. An about 8.0 to 9.0 minutes time was spent on each phone call with each participant to deliver diabetes education.

### Number of sessions

Considering 3 SMS and 1 phone call per month, 24 diabetes educational messages were sent and 8 phone calls were made to each participant during the study period of first 8 months. Considering the patient feedback and their preference to phone calls over SMS, 24 phone calls were made for the extended study period of 14 months.

### Frequency of camps

Diabetes educational camps were conducted every 4^th^ month, where each camp lasted for ~4 hours. Participants were re-oriented on diabetes management and diabetes self-management practices followed by HbA1C investigations.

### Sample collection and analysis

Blood (2.5 mL) was collected and HbA1c level estimated using Ion Exchange Chromatography (HPLC; D-10 Bio-Rad Hemoglobin Testing System) method at base line, 8 months and 14 months of intervention. HbA1c investigations were conducted free of cost as an incentive for participation in the study. As per the American Diabetes Association, HbA1c less than 5.7% was considered to be normal. Individuals with a HbA1c value between 5.7%-6.5% were considered Pre-diabetic and those with HbA1c level higher than 6.5% were considered as individuals with diabetes. If the HbA1c values of the diabetic patient is less than 5.7 (due to treatment or any other intervention(s), the patient is referred as under excellent glycemic control. If the HbA1c is between 6 to 7, the patient referred as under good control. Any value above 7 is referred as poor glycemic control.

#### Primary and secondary outcome measures

A decrease in 0.5% HbA1c value is the primary outcome. The secondary outcome measures of the study include knowledge enhancement in diabetes management practices.

### Statistical analysis

Variations in the HbA1C value for each individual was recorded at base line, 8-months and 14-months of intervention and analyzed by performing repeated measures ANOVA. In brief, the base-line value was compared with 8-months, and 14-months intervention value and between 8-months to 14-months intervention values. P value of < 0.05 was considered significant. In addition, the mean, standard deviation (SD) and standard error (SE) values of HbA1c at base line, 8-months post-intervention and 14-months post intervention were also calculated and compared as above. Analysis was carried out using Graph Pad Prism software. A decrease in HbA1C (a positive change) and an increase in HbA1C (a negative change) at 8-months post intervention and 14-months post intervention were also marked and compared to assess whether a 14-months period was required to observe a significant positive change in the study subjects. An increase in HbA1C might be due to poor adherence of individuals to the prescribed medication as reported in many other studies of similar intervention [12]. A Chi square (χ^2^) test was performed to examine if the frequencies in the cells varied at baseline and at 8 and 14 months. The comparison of HbA1C values <7.0 at base line and 14months intervention yielded significant (P <0.05) result.

## Results and discussion

Primary aim of this study was to determine whether intervention through a mobile SMS or phone call or both, helps in better management of diabetes, thereby reduce the complications associated with diabetes in a rural setting of Mysuru district, Karnataka, India. Experimentally, the recruited study participants were provided with information through power point presentation about the diabetes and its management (S4 & S5 Files), and followed up with SMS and or Phone calls every week as detailed in experimental section (S10, S14 & S15 Files).

### Demographics of study population

The study was conducted in a group of individuals (n = 380; Male: 209 and Female: 171) coming from Mysuru (n = 276; 72.6%), Mandya (n = 76; 20%), Chamarajanagara (n = 19; 5%) and Hassan (n = 9; 2.3%). The mean age of participants varied from 55 ± 2.4 years in males and 54 ± 2.5 years in females (Table 1). Forty five percent (n = 171) participants were from rural areas. The remaining participants were from semi-urban (n = 124; 32.6%) and urban (n = 85; 22.3%) areas (Table 1). Professionally, 42.3% of study participants were homemakers (n = 161), while other individuals belong to agriculture (n = 144; 37.8%), service industry (n = 47; 12.3%) and office (n = 28; 7.3%) (Table 1). Analysis of the individuals for average years of diabetes showed that male participants had a mean of 7.8 ± years compared to females with a mean of 7.1 ± years (Table 1).

**Table 1:** Demographic information of study participants.

Demographics	N = 380	% of total
Male	209	55
Female	171	45
**Age**		
**Gender**	**Mean value**	
Male	55	
Female	54	
**Age Range**		
**Age Range (Years)**	**Number of individuals**	**% of total**
31–40	29	7.6
41–50	114	30
51–60	86	22.6
61–70	104	27.4
71–80	47	12.4
**Location**		
**Location**	**Number of individuals**	**% of total**
Mysuru	276	72.6
Mandya	76	20
Chamrajanagara	19	5
Hassan	9	2.3
**Domicile**		
**Domicile**	**Number of individuals**	**% of total**
Urban	85	22.3
Semi-urban	124	32.6
Rural	171	45
**Profession**		
**Profession**	**Number of individuals**	**% of total**
Agriculture	144	37.8
Office	28	7.3
Service industry	47	12.3
Homemaker	161	42.3
**Average Years of diabetes (Mean)**		
**Gender**	**Mean Value**	
Male	7.8	
Female	7.1	
**Diabetes associated comorbidities**		
**Comorbidities**	**Number of individuals**	**% of total**
Hypertension	133	35
Thyroid disorders	10	2.6
Joint pains	19	5
Retinopathy	19	5
Renal disorders	57	15
Diabetic foot	10	2.6

Patients’ demographic details such as age, location, domicile and profession were captured along with number of years being diabetic. In addition, data pertaining to diabetes-associated co-morbidities such as hypertension, thyroid disorders, joint pains, retinopathy, renal disorders and diabetic foot were represented in the table.

Among various co-morbidities, 35% participants were suffering from hypertension (blood pressure value > 120/80 (n = 133) (Table 1). Among the other co-morbidities renal disorders (diabetic nephropathy) were observed in 15% (n = 57); while 5% individuals had retinopathy (n = 19) and another 5% had joint pains (n = 19). Only 2.6% individuals reported diabetic foot (n = 10) and thyroid disorders (n = 10) respectively. 44 individuals (11.5%) did not report any of these co-morbidities at the time of study commencement.

### Assessment of diabetes management practices

Disease management practices such as timely health checks; adherence to medications, dietary restrictions, and attending physical activity programs; and awareness about self-management are key factors contributing to the overall health of a diabetic individual [13–17]. Therefore, in this study, knowledge and adherence to these parameters have been assessed using structured questionnaire. Analysis of the data showed that: (a) all the participants visited the doctor at least once in six months. Further analysis showed that 65% participants (n = 247) had the habit of visiting the doctor at least once in 3 months; (b) 50% (n = 190) participants were checked for blood analysis at least once in a month (Table 2). Among various blood investigations, 57.6% had their fasting blood glucose checked while 37.3% (n = 142) had post-prandial blood sugar and 5% (n = 19) had random blood sugar tests performed.

**Table 2:** Participants’ disease management practices.

	N = 380	% of total
**Frequency of doctor visit**		
**Period**	**Number of individuals**	**% of total**
1 month	114	30
3 months	247	65
6 months	19	5
More than 6 months	0	0
**Frequency of blood investigation**		
**Period**	**Number of individuals**	**% of total**
1 month	190	50
3 months	171	45
6 months	9	2.3
More than 6 months	10	2.6
**Type of blood investigations**		
**Investigation**	**Number of individuals**	**% of total**
Fasting	219	57.6
Post prandial blood sugar (PPBS)	142	37.3
Random	19	5
HbA1C	0	0
**Medication regimen**		
**Medication**	**Number of individuals**	**% of total**
Single oral hypoglycemic agents	162	42.6
Multiple oral hypoglycemic agents	85	22.3
Insulin	133	35
**Adherence to Dietary restrictions**		
**Adherence**	**Number of individuals**	**% of total**
Always	143	37.6
Sometimes	218	57.3
Never	19	5
**Time spent on physical activity**		
**Time**	**Number of individuals**	**% of total**
<1 hour/ day	304	80
1–2 hours/ day	47	12.3
Not everyday	29	7.6
**Frequency of foot inspection**		
**Frequency**	**Number of individuals**	**% of total**
Always	57	15
Sometimes	228	60
Never	95	25
**Frequency of eye inspection**		
**Frequency**	**Number of individuals**	**% of total**
Always	57	15
Sometimes	228	60
Never	95	25
**Awareness on self-management of diabetes**		
**Awareness**	**Number of individuals**	**% of total**
Yes	333	87.6
No	47	12.3

Details regarding patients’ disease management practices such as frequency of doctor’s visit, blood investigations and types of investigations, medication, diet control practices, time spent on physical activity, frequency of foot and eye inspection along with the awareness on self-management of diabetes were reported in this table.

Interestingly none of the participants had ever tested for HbA1C before the study. Since HbA1C is an important determinant of the average 3months blood sugar and an indicator of glycemic control, it is essential that enough instructions and education should be provided to the individuals with diabetes about the investigation [18–22]. Lack of awareness and cost of HbA1C investigation (Vary from Rs. 200 to Rs. 700 depending on the city) were the key factors for not getting this test done periodically [23]. Offering these investigations under subsidized prices by government health schemes or diabetes control programs would encourage patients to undergo this test periodically. Further, research is also required to invent an economical and easy-to-perform strip based HbA1C test (similar to regular blood glucose monitors), making it comfortable to diabetic patients in periodic monitoring of blood glucose [24].

Diabetes can be well-controlled if the effected individual practices good diet, perform regular exercise and adhere to prescribed medicines. Testing these practices in the study participants showed that 57.3% (n = 218) individuals followed the dietary restrictions once a while (Table 2). About 37.6% individuals followed strict diet control every day. 5.0% participants (n = 19) never had any dietary restrictions (Table 2). Interestingly 80% individuals spent <1 hour/day in physical activity, whereas 12.3% reported that they were spending 1–2 hours/day in performing some physical activity (Table 2). 7.6% participants mentioned that they were not performing any physical activity (Table 2). Analysis of the medication regimen showed that 42.6% (n = 162) were on single oral hypoglycemic agent, while 35.0% of them were on insulin, and 22.3% were on multiple oral hypoglycemic agents.

As diabetic patients have increased risk towards retinopathy and foot ulcers, the frequency of eye and foot examinations was assessed [25–27]. Interestingly 60% participants (n = 228) reported that they had eye and foot examinations performed sometimes but not on regular basis. Only 15% (n = 57) had these tests on regular basis. 25% participants (n = 95) reported that they had ever tested for their HbA1C (Table 2).

Despite variations in participants’ response to the above-mentioned parameters, 87.6% (n = 333) individuals had mentioned about their awareness pertaining to self-management programs of diabetes (Table 2). Therefore, in addition to educating the individuals about diabetes through self-management programs continuous monitoring, follow-up and providing cost effective self-management/examination devices are also important to combat the diabetes.

### Influence of SMS and phone calls on participants’ knowledge about diabetes

Although 87.6% study participants reported their awareness about self-management of diabetes, determining the most effective educational tool that works better is yet to be identified. Hence, this pilot study focused on studying the influence of SMS and phone call-based diabetes management education on participants’ glycemic control and knowledge about various diabetes related aspects. Even though the study recruited 380 participants, only 120 (~1/3^rd^ of total participants) could complete the entire duration of 14months. Remaining 260 participants (2/3^rd^ of total) could not complete the study due to (a) lack of proper connectivity to the study site (31% of defaulters; n = 80); (b) moving to other places, which are much farther from study site (15% of defaulters n = 40); and (c) other reasons such as poor adherence to study, not attending the study camps etc (54% of defaulters; n = 140) (Supplemental information). Hence, the data in Table 3 is restricted to 120 participants. Analysis of the data after intervention showed that all the participants were aware of symptoms of diabetes. Among various symptoms, excess thirst was reported by 55.8% (n = 67) individuals; while 28.3% (n = 34) mentioned about frequent urination (Table 3).

**Table 3:** Knowledge assessment about diabetes management after intervention.

Knowledge Assessment	N = 120	% of total
**Symptoms of diabetes**		
**Symptoms**	**Number of individuals**	**% of total**
Excessive thirst	67	55.8
Hunger	30	25
Frequent urination	34	28.3
Weight loss	30	25
**Best practices of diabetes management**		
**Best Practices**	**Number of individuals**	**% of total**
Regular exercise	90	75
Controlled diet	94	78.3
Medication adherence	75	62.5
Periodic doctor visit	56	46.6
**Complications of Uncontrolled diabetes**		
**Complications**	**Number of individuals**	**% of total**
Heart disease	19	15.8
Eye problems	71	59.1
Stroke	21	17.5
Neurological diseases	22	18.3
Kidney diseases	30	25
Foot ulcers	49	40.8
**Symptoms of hypo-glycemia**		
**Symptoms**	**Number of individuals**	**% of total**
Dizziness	30	25
Hunger	26	21.6
Sweating	45	37.5
Shakiness	34	28.3
Anxiety	22	18.3
Moodiness	30	25
**Excellent glucose range**		
**Glucose value/ range**	**Number of individuals**	**% of total**
<80	0	0
80–120	90	75
120–180	30	25
>180	0	0
**Investigation that gives accurate glucose reading**		
**Investigations**	**Number of individuals**	**% of total**
HbA1c	113	94.1
PPBS	0	0
Fasting	7	5.8

Patient’s knowledge on diabetes self-management was assessed after intervention which included identification of diabetes symptoms, best practices for diabetes management, diabetes associated complications, symptoms of hypo glycemia, good glucose control range along with diabetes investigations were detailed.

Participants had confirmed their knowledge about best practices in keeping diabetes under control. As shown in Table 3, 75% participants were aware about the key role played by regular exercise and controlled diet (78.3%) in maintaining good glyceamic control, followed by medication adherence (62.5%) and periodic doctor visits (46.6%). Likewise, about 59.1% (n = 71) individuals reported that they were aware of complications related to eyes after providing continuous education on diabetes management. Among other complications, knowledge about foot ulcers was reported by 40.8% (n = 49). About 75% (n = 90) participants acquired the knowledge about normal blood glucose range ie., 80-120mg/dL. Encouragingly, due to the diabetes education provided in this study, >94.1% (n = 113) participants now reported that they were aware of good HbA1C reading ie., <6.0%.

Educating individuals with diabetes about hypoglycemic condition is also an equally important factor one should consider while designing knowledge providing materials [28]. Hence, in the current study, participants were educated about hypoglycemia and various methods of handling hypoglycemic condition. The SMS and phone call intervention showed that 37.5% (n = 45) participants were aware about sweating when a person experiences hypoglycemia (Table 3). Study participants had also reported shakiness (28.3%; n = 34), dizziness (25%; n = 30) and moodiness (25%; n = 30). In summary, the intervention had improved participants’ knowledge about diabetes and self-management behaviors.

### Assessment of patient feedback on acceptance of SMS and phone calls

India has a greater number of mobile phone users. According to recent statistics, currently, India has more than 800 million mobile users [29]. Therefore, education programs, such as the one implemented in this study, through mobile phones will be of high-reach and provide better visual impact and personal connectivity. SMS and phone calls were found to be the two widely used and feasible intervention strategies adopted to educate patients about self-management of chronic diseases [30–32]. However, it is unknown which of these two modes have better acceptability and impact. Hence, in our study we had assessed the impact of SMS and phone calls on diabetes management among participants; and feedback on the study was obtained.

Overall, 94.1% (n = 113) participants expressed their satisfaction about the diabetes self-management orientation program. Majority of participants (78.3%; n = 94) were of the opinion that the orientation classes should be conducted at least once in 3 months (Table 4). Interestingly, all the participants (100%, n = 120) felt that the orientation classes were of great help in the self-management of diabetes. Between SMS and phone calls, about 81.6% (n = 98) individuals expressed their comfort with weekly SMS. Similarly, 75% individuals (n = 90) showed their interest in receiving monthly phone calls (Table 4). However, although 81.6% individuals expressed comfort with weekly SMS, the most preferred mode of intervention was found to be through phone calls over SMS, which was due to (a) the participants satisfaction when they were approached through phone; (b) interactive nature of phone call rather than reading a message (as in SMS) and (c) both reasons. In summary, results of this pilot study found better response to phone calls over SMS for the self-management of diabetes.

**Table 4:** Patient feedback on SMS & phone call interventions.

Feedback	N = 120	% of total
**Satisfaction on diabetes self-management orientation class**		
**Satisfaction**	**Number of individuals**	**% of total**
Satisfied	113	94.1
Unsatisfied	7	5.9
**Frequency of orientation classes required**		
**Frequency**	**Number of individuals**	**% of total**
1 month	11	9.1
3 months	94	78.3
6 months	15	12.5
**Comfortable with weekly SMS**		
**SMS**	**Number of individuals**	**% of total**
Yes	98	81.6
No	22	18.3
**Comfortable with monthly phone calls**		
**Phone calls**	**Number of individuals**	**% of total**
Yes	90	75
No	30	25
**Study helps in self-management of diabetes**		
**Satisfaction on current study**	**Number of individuals**	**% of total**
Yes	120	100
No	0	0
**Preferred mode of intervention**		
**Participants preference**	**Number of individuals**	**% of total**
SMS	22	18.3
Phone calls	98	81.6
**Reason for preferring phone calls (multiple options)**		
**Reasons**	**Number of individuals**	**% of total**
Feels good to talk than read	67	55.8
Interactive communication	97	80.8
Both	113	94.1

Patient’s satisfaction on periodic re-orientation programs on self-management of diabetes, their comfort with weekly SMS and monthly phone calls, preference of SMS over phone call intervention and the reasons were obtained and analyzed.

### Education through phone calls reduced HbA1C in ~47% of study participants

Since the pilot study (Results at 8 months period) showed the preference of phone calls over SMS for the self-management of diabetes, the study was further continued to determine the benefit of weekly phone call-based diabetes education on improving participants HbA1C values and knowledge on diabetes self-management practices.

Analysis of the data showed about 47% individuals (55 out of 120 participants) showed a decrease in HbA1C (0.1 to 2.6 units) when tested after 8 months of intervention through SMS & mobile phone calls (P<0.05 by repeated measures ANOVA). Two individuals had no change in the HbA1C. Among the remaining 63 participants 44 individuals showed a slight increase <1.0 Unit in HbA1C, while 19 had an increase above 1 unit of HbA1C. Further analysis of the results showed a 0.3 to 2.6 units reduction in HbA1C in about 47.5% (57 out of 120) of study participants educated for 14 months (P<0.05 by repeated measure ANOVA).

Additional analysis of the data determining the number of individuals in different HbA1C categories (<5.0; 5.1–7.0; 7.1–9.0; 9.1–11.0; 11.1–13.0; and 13.1–15.0) showed a significant increase in the number of individuals with HbA1C in the range of 5.0 to 7.0 (from 27 at base line to 37 after 14months intervention) (Table 5; P<0.05 by repeated measure ANOVA; Refer S18 File for variations in individual HbA1C values). Since HbA1C in this range indicates a good control, the increase in number of individuals in this group is a positive sign of intervention through phone calls. A decrease in the number of individuals from 26 at base line to 21 at 14months intervention was observed in the 9.1–11.0 HbA1C group. Decrease in the number of individuals in this range is another positive sign as this HbA1C range shows poor control over diabetes. Only three individuals showed elevated HbA1C in the 13.1–15% group (Table 5; 2 individuals at base line to 5 individuals after 14months intervention; Refer S18 File for variations in individual HbA1C values). This increase could be due to the poor adherence to medication or phone call based instructions. In summary, although there was no much improvement when overall data was analyzed (53% individuals did not show improvement in the HbA1C readings), the intervention through phone calls increased the number of individuals with HbA1C in the range of 5.1–7.0, indicating that it could be a viable strategy to better manage individuals who are in the pre-diabetic state. A Chi square (χ^2^) test was performed to examine if the frequencies in the cells varied at baseline and at 8 and 14 months. Even though overall analysis showed no significant difference (P > 0.05) at base line and 8 months intervention; the comparison of HbA1C values <7.0 at base line and 14months intervention yielded significant (P <0.05) result.

**Table 5:** Comparison of HbA1C values throughout the study.

HbA1C Range	Baseline Data		Data at 8 Months		Data at 14 Months	
	Number of Participants	Baseline HbA1C Values	Number of Participants	HbA1C Values at 8 Months	Number of Participants	HbA1C Values at 14 Months
<5	0	0	0	0	5	4.16±0.06
5.1–7	27	6.4±0.08	37	6.2±0.09	37	6.2±0.09
7.1–9	55	8.0±0.08	38	7.7±0.08	42	8.1±0.08
9.1–11	26	9.9±0.10	29	9.8±0.10	21	9.9±0.11
11.1–13	10	12.2±0.13	12	12.1±0.16	10	11.8±0.17
13.1–15	2	13.9±0.10	4	14.3±0.46	5	13.8±0.20

Comparison of HbA1C investigations across various time points during the study were mentioned. Range of HbA1C values, number of participants at baseline, 8 months and 14 months along with average HbA1C values and standard error were computed. Comparisons were made and statistical significance was determined using repeated measures ANOVA. The data was compared between baseline-8 months, baseline-14 months and 8 months-14 months found highly significant with P<0.0001.

Recent studies by Abaza, H et al have reported a significant improvement in the diabetes management and HbA1C through SMS based intervention for managing diabetes [33]. Russell E. Glasgow et al reported significant improvement in behavioral, psychological and few biological outcomes when internet based self-management diabetes program (D-Net) was implemented for a period of 10months in a population of 320 adult patients suffering from type2 diabetes [34]. However, a non-significant increase (7.44 at baseline to 7.55 after 10 months; P value 0.210) was reported in the study [35]. In another study, Moyano, D., et al., assessed the perceptions and acceptability of SMS text messaging for diabetes care in a primary care setting in Argentina by using a 24-semi-structured questions, and showed that SMS-has a positive contribution in the care of individuals with diabetes [36]. A separate pilot study by Dobson R., et al., evaluated the ability of Self-Management Support for Blood Glucose (SMS4BG), a tailored text message-based intervention, among adults with uncontrolled diabetes. Authors reported that SMS4BG is acceptable and useful in supporting self-management in people with poorly controlled diabetes. However, authors have concluded that a longer duration (more than 3 months) efficacy study is required for further validation and suitability assessment [37]. Unlike these reported studies, results of current study showed a decrease in HbA1C by 0.3 to 2.6 units in 47.5% study participants after 14 months of intervention; indicating that phone call-based health education could be a better intervention method. A case control study evaluating the effect of phone calls is immediately required to conclusively ascribe and suggest phone call-based intervention for the management of diabetes.

Rosal, M.C., et al conducted a community-based diabetes self-management educational intervention by providing information on diabetes knowledge, attitudes, and self-management skills through culturally specific and literacy-sensitive strategies for a duration of 6 months. The study demonstrated a significant decrease in HbA1C by 0.8 units in the intervention group at 3-months and 6-months period [35]. Further, an increase in physical activity and self-monitoring of glucose was reported among study participants (interventional group n = 15) compared to non-interventional group (n = 10). In the current study, the interim analysis on knowledge assessment showed improvement in the secondary outcomes such as a) HbA1C (T_b_−None, T 8months− 93.5%). At baseline, none of the participants were aware of HbA1C investigation, however, after 8 months of educational intervention using SMS and Phone calls, the knowledge on HbA1C investigation and its importance in indicating glyceamic control over a period of 3-months was enhanced to 93.5%. Similarly, improvement in the following parameters was observed. b) Diet control (T_b_− 37.5%, T 8months− 78.1%; 2.1-fold increase) c) Eye examination (T_b_− 15%, T 8months− 59.3%; 3.9-fold increase) d) Foot examination (T_b_− 15%, T 8months− 32.5%; 2.16-fold increase)

*T_b-_ Timepoint at baseline, T_8months-_ Timepoint at 8 months Unlike the study by Rosal, M.C., et al, the participant adherence to the current study was much lower; 78% in Rosal M. C. et al VS 31.5% in the current study; which could be due to various reasons depicted in S19 File.

In summary, while finding the better educational mode (SMS/Phone call) for self-management of diabetes, the study could assess the feasibility of phone call-based intervention in reducing the HbA1C, an indicator of diabetes control. However, this study doesn’t rule out the benefits of using SMS as this route might be useful for those individuals with hearing ailments. Further evaluation in a large study population would provide more confident data, by which one can strongly recommend phone calls for not only managing diabetes but also to use this as a strategy to treat individuals with diabetes. Future studies should also consider overcoming the following barriers a) Effective way of communicating with the study participants in their own language and making them understand the study concept. In this regard prior sensitization using short videos on diabetes management might help b) Adherence to the time schedule for the phone calls was found to be another hinderance as majority of the participants do not have structured work schedules c) participants willingness to attend the scheduled camps.

Although the study results were satisfactory in identifying the phone call-based diabetes education as a preferred mode of intervention, a study by Matthew S. Thiese stated that the outcomes of these single arm, pre-post study designs are temporary, which could be influenced by several other factors. Therefore, the study outcomes and changes experienced in the results cannot be completely attributed to the intervention [38]. Despite of these limitations, single arm studies were proven to provide efficacy and safety of pilot data [39]. Further, minimal compliance of participants and limited generalizability to populations not included in the study are other drawback quiet often observed in single arm studies [40]. Moreover, the results reported using single arm studies may not be comparable as factors other than intervention also likely to play a role in the analysis of results. Despite these limitations, single-arm studies are widely used in clinical trials particularly to acquire the safety and efficacy data in many intervention studies [41]. In the current pilot study, the impact of phone call-based diabetes education intervention was found to improve HbA1C value among study participants and resulted in the gain of knowledge on diabetes management. Further studies are warranted to conclusively test and establish the utility of phone call-based intervention in a case-control study.

## Supporting information

S1 File Institutional ethical clearance certificate. The ethical clearance from the institution for conducting the current study was obtained from Institutional Ethical Committee, JSS Medical College, JSS Academy of Higher Education & Research.
(PDF)

S2 File Study protocol. The protocol describes the design, participant selection and method of conducting the study.
(PDF)

S3 File Demographics of study participants and their diabetes management practices. The sheet was designed to capture participants demographics and baseline data on diabetes management practices.
(PDF)

S4 File Orientation material on diabetes education in English and local language Kannada. Diabetes orientation/reorientation educational material was presented in English and local language Kannada.
(PDF)

S5 File Orientation material on diabetes education in English and local language Kannada. Diabetes orientation/reorientation educational material was presented in English and local language Kannada.
(PDF)

S6 File Informed consent forms in English & local language Kannada. Informed consent forms were made in English and local language Kannada to obtain participants consent for enrolling into the study.
(PDF)

S7 File Informed consent forms in English & local language Kannada. Informed consent forms were made in English and local language Kannada to obtain participants consent for enrolling into the study.
(PDF)

S8 File Handouts provided to study participants. Participants were given handouts containing information pertaining to diabetes education and pictures that reinforces their learning during orientation. The handout also facilitates them to enter blood investigations readings and, to keep track of their glucose levels. Handouts were made available in local language Kannada as well as in English.
(TIF)

S9 File Handouts provided to study participants. Participants were given handouts containing information pertaining to diabetes education and pictures that reinforces their learning during orientation. The handout also facilitates them to enter blood investigations readings and, to keep track of their glucose levels. Handouts were made available in local language Kannada as well as in English.
(TIF)

S10 File SMS details sent to the participants in English and local language Kannada. Periodic messages (SMS) that were sent to the participants during the study period of first 8 months.
(PDF)

S11 File SMS log sheet. The log sheet was designed to enter the collected information such as patient name, mobile number and SMS delivery status every time an SMS was sent to the study participant.
(PDF)

S12 File Participants feedback and diabetes knowledge assessment questionnaire. Participants’ knowledge on diabetes management practices was assessed using a simple structured questionnaire along with their feedback on study design.
(PDF)

S13 File Scheme. The scheme shows the overall study design. The study initially recruited 380 participants with diabetes. These participants had received diabetes education through weekly SMS and monthly phone calls for a period of 8months. Upon considering the participants feedback preferring phone calls over SMS messages, weekly phone call-based diabetes education was provided for an additional 6-months period. A total of 120 participants completed the 14 months study.
(PDF)

S14 File Phone call script in English and local language. The telephone call script drafted in English and local language Kannada was used for telephonic conversations with study participants for delivering diabetes self-management education as well to capture the disease management practices.
(PDF)

S15 File Phone call script in English and local language. The telephone call script drafted in English and local language Kannada was used for telephonic conversations with study participants for delivering diabetes self-management education as well to capture the disease management practices.
(PDF)

S16 File Weekly phone call log. The weekly phone call log sheet was designed to cross verify the delivery of diabetes educational materials as well as to add any additional remarks mentioned by study participants.
(PDF)

S17 File Diabetes practice evaluation form. The form was designed to capture information related to participants’ disease management practices every month after receiving weekly diabetes management education.
(PDF)

S18 File HbA1c values of study participants. The figure demonstrates the HbA1c values of 120 study participants across baseline, 8 months and 14 months of study periods.
(TIF)

S19 File Reasons for study drop-outs. The doughnut graph depicts the various reasons for 2/3rds of the participants non-adherence to the current study.
(TIF)

S20 File (ZIP)
