# Supplementary material for: Intervention through Short Messaging System (SMS) and phone call alerts reduced HbA1C levels in ~47% type-2 diabetics–results of a pilot study
Source: PLoS One. 2020 Nov 17;15(11):e0241830. doi: 10.1371/journal.pone.0241830 (PMC7671489; doi:10.1371/journal.pone.0241830)
Supplement: S1 File — The ethical clearance from the institution for conducting the current study was obtained from Institutional Ethical Committee, JSS Medical College, JSS Academy of Higher Education & Research. (PDF) [file pone.0241830.s001.pdf]

JSS MEDICAL COLLEGE
(Constituent College)
Jagadguru Sri Shivarathreeshwara University
(Deemed to be University)
Accredited 'A' Grade by NAAC

JSS UNIVERSITY

Members

Dr. M.Premanath
MD. General Medicine
Chairman

Dr.H.Basavana Gowdappa
MD. General Medicine
Member

Dr. R.Rajalakshmi
MD Physiology
Member

Dr.M.Guruswamy
MS. ENT
Member

Sri.NagendraMurthy M.P
B.Com. LL.M, M.Phil.
Member

Smt. Sudhaphaneesh
M.A., B.Ed.,(Ph.D)
Member

Dr.Madan Ramesh,
M.Pharm, PhD
Member

Sri. R.S.Nagaraj
B.Sc, MBA
Member

Dr.R.N.Suresha
MD. Pharmacology
Member Secretary

Dr. P. Salimath
Director Research
Special Invitee

JSSMC/IEC/11 / 5976 /2016-17 Date: 30.11.2016

INSTITUTIONAL ETHICAL COMMITTEE

CERTIFICATE

This is to certify that the below mentioned Project has been cleared and approved by the Institutional Ethical Committee at its meeting held on 29.11.2016.

**Title of Project**	" Effectiveness of Self Management in Type 2 Diabetic Patients Through ICT Interventions."
**Principal Investigator**	**Mrs. Kanakavalli K Kundury**<br>Lecturer<br>Dept of Health System Management Studies,<br>JSS University<br>Mysore

30/11/16

MEMBER SECRETARY
Member Secretary,
Institutional Ethical Committee
J.S.S. Medical College, S.S.
MYSORE-570 015

Sri Shivarathreeshwara Nagara, Mysuru - 570 015, Karnataka, India
P : +91 821 2548337 | 2548338 | F : +91 821 2548345 | 2493819
E : jssmc@jssuni.edu.in | jssmc09@gmail.com | W : www.jssuni.edu.in

Scanned by CamScanner
