# Supplementary material for: Intervention through Short Messaging System (SMS) and phone call alerts reduced HbA1C levels in ~47% type-2 diabetics–results of a pilot study
Source: PLoS One. 2020 Nov 17;15(11):e0241830. doi: 10.1371/journal.pone.0241830 (PMC7671489; doi:10.1371/journal.pone.0241830)
Supplement: S2 File — The protocol describes the design, participant selection and method of conducting the study. (PDF) [file pone.0241830.s002.pdf]

Annexure4- Study Protocol

Health education intervention through mobile phone calls for self-management of diabetes

Objectives:

The objective of the study is to determine the feasibility and utility of Phone Call based health education intervention in diabetes management and to measure it’s impact on HbA1c values

Study design:

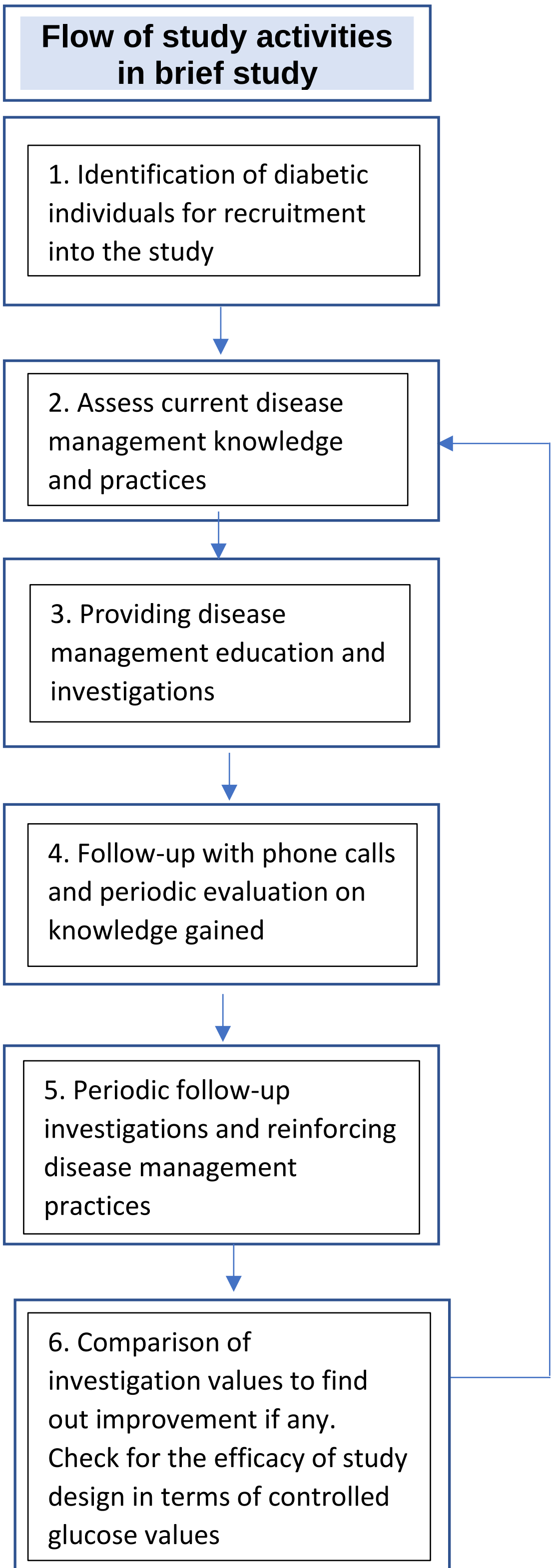

Study Methodology:

- Patient's with confirmed type 2 diabetes were identified for recruitment into the study with informed consent
- Participants will be oriented on study design and education will be provided by a physician and a dietician on diabetes care management practices such as fundamentals of diabetes, disease symptoms, associated complications, role of self-care in diabetes, practices to keep diabetes under control which includes diet control, medication adherence, physical activity, periodic blood investigations and follow-up visits to doctor.
- Baseline data such as participants demographics, years of having diabetes, current medications and contact telephone number of the participants will be collected
- HbA1c investigations will be conducted to the participants and baseline values are collected
- Patients will be then receiving diabetes educational message once a week on their telephone number and a follow-up phone call once a month to assess their diabetes care management behaviours.
- A follow-up camp will be conducted every 4th month to re-orient the participants on diabetes care and HbA1c investigations to be conducted
- The HbA1c results will be compared to identify the impact of health education intervention on glycemic control of diabetics.

Participants selection:

Inclusion criteria:

- Patients with confirmed type2 diabetes
- Should be visiting JSS Hospital
- Able to follow English or Kannada
- Should have basic knowledge of operating mobile phones

Exclusion criteria:

- Type 2 diabetics suffering from severe diseases/ infections

Study site:

Subjects identified by The Department of General Medicine, JSS Hospital, Mysuru, with confirmed diabetes of Type2 will be recruited into the study. HbA1c analysis will be conducted at Biochemistry department of the hospital

Sample size:

Adopting a voluntary sampling technique, interested patients with confirmed diabetes will be recruited into the study. The results of the study in terms of the improved results of HbA1C values and factors such as participant drop out of the study helps to determine the sample size required for conducting the main study.

Statistical methods:

HbA1c values obtained ta various time points will be compared with the baseline data obtained. The impact of health education intervention will be obtained by classifying the HbA1C values into various ranges and comparing with the number of participants within each range across various timepoints. Average values of HbA1C will be computer along with standard deviation to know the deviation of respective values with the mean value.

Ethical clearance:

Ethical clearance was obtained from Institutional Ethical Committee, JSS Medical College, JSS Academy of Higher Education & Research, Mysore. (JSSMC/11/5976/2016-17; Dated: 30.11.2016)

Data handling:

The data captured at various timepoints will be recorded in to Microsoft Excel sheets and saved for analysis. Hardcopies of the forms filled, questionnaires administered will be tagged with the informed consent forms of the respective study participant and periodic HbA1c reports will be filed along.

Study Outcomes:

The results obtained from the study will be analysed to identify the influence of health education intervention through phone calls on self-management of diabetes on HbA1c values of the study participants. The results thus obtained will be used to write manuscripts for publication into reputed journals, so that it helps to plan further research on similar grounds.

Project timelines:

Timeline (Months)	Activity	Progress Indicator
0-3	Preparations for conducting the study. Identifying confirmed type2 diabetic patients for enrolment into the study	Check on inclusion and exclusion criteria, confirmation and communication about orientation camp for the start of the study. Preparation of calling scripts and data collection forms
4-12	Study commences, informed consent from participants, orientation, collecting patient profile, baseline data along with HbA1c investigations. Obtaining patient feedback on study design, to check their comfort with Short Message Service and telephone calls. Periodic HbA1c investigations to be conducted	Record keeping, communicating investigation results, initiating weekly messages on diabetes education and monthly follow-up with phone calls. Diabetes Knowledge assessment forms after health education intervention, feedback forms to be made ready. Analysis of feedback obtained to modify study design, diabetes knowledge assessment, follow-up with phone call interventions

13-19	Obtaining patient feedback on study design, to check their comfort with Short Message Service and telephone calls. Periodic HbA1c investigations to be conducted<br>Considering participant feedback to make finer changes in the current study design, if required	Analysis of feedback obtained to modify study design, diabetes knowledge assessment, follow-up with phone call interventions<br>Implementing the intervention for further follow-up
20-22	Data analysis to identify the impact of health education intervention on HbA1c values	Manuscript preparation and communication for journal publication. To plan future research projects

References:

1. Margaret A. Powers, Joan Bardsley,Marjorie Cypress, Paulina Duker, Martha M. Funnell, Amy Hess Fischl, Melinda D. Maryniuk, Linda Siminerio,and Eva Vivian. Diabetes Self-management Education and Support in Type 2 Diabetes: A Joint Position Statement of the American Diabetes Association, the American Association of Diabetes Educators, and the Academy of Nutrition and Dietetics; Diabetes Care 2015;38:1372–1382
2. James E. Aikens, Kara Zivin, Ranak Trivedi,John D. Piette. Diabetes self-management support using mHealth and enhanced informal caregiving; J Diabetes Complications. 2014 ; 28(2): 171–176.
3. John D. Piette, Ann-Marie Rosland, Nicolle Stec Marinec, Dana Striplin, , Steven J. Bernstein, Maria J. Silveira. Engagement with Automated Patient Monitoring and Self-Management Support Calls: Experience with a Thousand Chronically-Ill Patients; Med Care. 2013 March ; 51(3): 216–223
4. http://patienteducation.stanford.edu/internet/diabetesol.html
5. Shilpa Joshi, Shashank R. Joshi, and Viswanathan Mohan. Methodology and feasibility of a structured education program for diabetes education in India: The National Diabetes Educator Program; Indian J Endocrinol Metab. 2013 May-Jun; 17(3): 396–401
6. Talha Riaz, Haris Riaz, Syed A Hussain and Danish Kherani; SMS reminders- future in self-care management of diabetes mellitus?Diabetology & Metabolic Syndrome 2012, 4:31
7. Josefien van Olmen, Grace Marie Ku, Maurits van Pelt, Christian Darras and Guy Kegels; Motivating betterdiabetes self-care withSMS text messaging; DiabetesVoice; June 2014, Volume 59.

8. Josefien van Olmen, Grace Marie Ku, Maurits van Pelt, Jean Clovis Kalobu, Heang Hen, Christian Darras, Kristien Van Acker, Balthazar Villaraza, Francois Schellevis and Guy Kegels; The effectiveness of text messages support for diabetes self-management: protocol of the TEXT4DSM study in the democratic Republic of Congo, Cambodia and the Philippines; BMC Public Health 2013, 13:423
9. Sheikh Mohammed Shariful Islam, Louis W. Niessen, Uta Ferrari, Liaquat Ali, Jochen Seissler and Andreas Lechner; Effects of Mobile Phone SMS to Improve Glycemic Control Among Patients With Type 2 Diabetes in Bangladesh: A Prospective, Parallel-Group, Randomized Controlled Trial; Diabetes Care; August 2015Volume 38.
10. Bree Holtz, Carolyn Lauckner, B.A; Diabetes Management via Mobile Phones: A Systematic Review; TELEMEDICINE and e-HEALTH; APRIL 2012; Vol18:3
11. Santosh Krishna, Suzanne Austin Boren; Diabetes Self-Management Care via Cell Phone: A Systematic Review; Journal of Diabetes Science and Technology; May 2008; Volume 2, Issue 3.
12. V. L. Franklin, A. Waller, C. Pagliari and S. A. Greene; A randomized controlled trial of Sweet Talk, a text-messaging system to support young people with diabetes; Diabetic Medicine, 2006, 23, 1332–1338
