# Supplementary material for: Intervention through Short Messaging System (SMS) and phone call alerts reduced HbA1C levels in ~47% type-2 diabetics–results of a pilot study
Source: PLoS One. 2020 Nov 17;15(11):e0241830. doi: 10.1371/journal.pone.0241830 (PMC7671489; doi:10.1371/journal.pone.0241830)
Supplement: S3 File — The sheet was designed to capture participants demographics and baseline data on diabetes management practices. (PDF) [file pone.0241830.s003.pdf]

Patient Demographics and Diabetes Self-Management Questionnaire

Patient ID: ______________ **Telephone:** _________________________

Name: ____

Age Group : 30 to 40 / 41 to 50 / 51 to 60 / 61 to 70 / 71 to 80

Gender: M ___ F ___

Address: ____

____

e-mail: _______________________________________

Domicile Category: Urban / Semi Urban / Rural

Work: Agriculture / Office / Service / Home Maker

Since how long are you diabetic? ________________

How frequently do you visit the doctor?

Once in 3 months / Once in 3-6 months / More than 6 months

How frequently do you get your blood testing done?

Once in 3 months / Once in 3-6 months / More than 6 months

What tests are normally done?

Fasting / PPBS / HbA1C / Lipid

Under what range will your HbA1C score stays

Less than 6 / Between 6 and 8 / Above 8

Do you adhere to the diet restrictions?

Always / Sometimes / Never

Under what kind of medication form are you in

Single therapy / Multiple therapy / Insulin

How much time do you spend every day on physical activity (walking, jogging, yoga or any fitness program)

Less than 1 hour / Between 1-2 hours / Not everyday

How often you inspect your foot and take eye examinations?

Always / Sometimes / Never

Are you aware of self-management of diabetes

Yes / No

Signature of the patient: ________________________________
