# Supplementary material for: Intervention through Short Messaging System (SMS) and phone call alerts reduced HbA1C levels in ~47% type-2 diabetics–results of a pilot study
Source: PLoS One. 2020 Nov 17;15(11):e0241830. doi: 10.1371/journal.pone.0241830 (PMC7671489; doi:10.1371/journal.pone.0241830)
Supplement: S4 File — Diabetes orientation/reorientation educational material was presented in English and local language Kannada. (PDF) [file pone.0241830.s004.pdf]

Diabetes Management

JSS Academy of Higher Education and Research
JSS Medical Institutions Campus
Sri Shivarathreeshwara Nagara
Mysuru – 570 015, Karnataka, INDIA
Phone: +91-821-2548416 / 400 **Fax:** +91-821-2548394

Diabetes

- Diabetes is a disease that occurs when your blood glucose, also called blood sugar, is too high
- Insulin, a hormone made by the pancreas, helps glucose from food get into your cells to be used for energy
- Sometimes your body doesn't make enough insulin or doesn't use insulin well
- Glucose then stays in your blood and doesn't reach your cells. having too much glucose in your blood can cause health problems

Diabetes

Type1:

- Body is unable to produce any insulin.
- Appears before the age of 40

Type2:

- Body can still make some insulin, but not enough
- Most commonly seen in ages above 40
- 85-90% diabetes is of this type

Gestational diabetes

-Arises during pregnancy

Symptoms of Diabetes

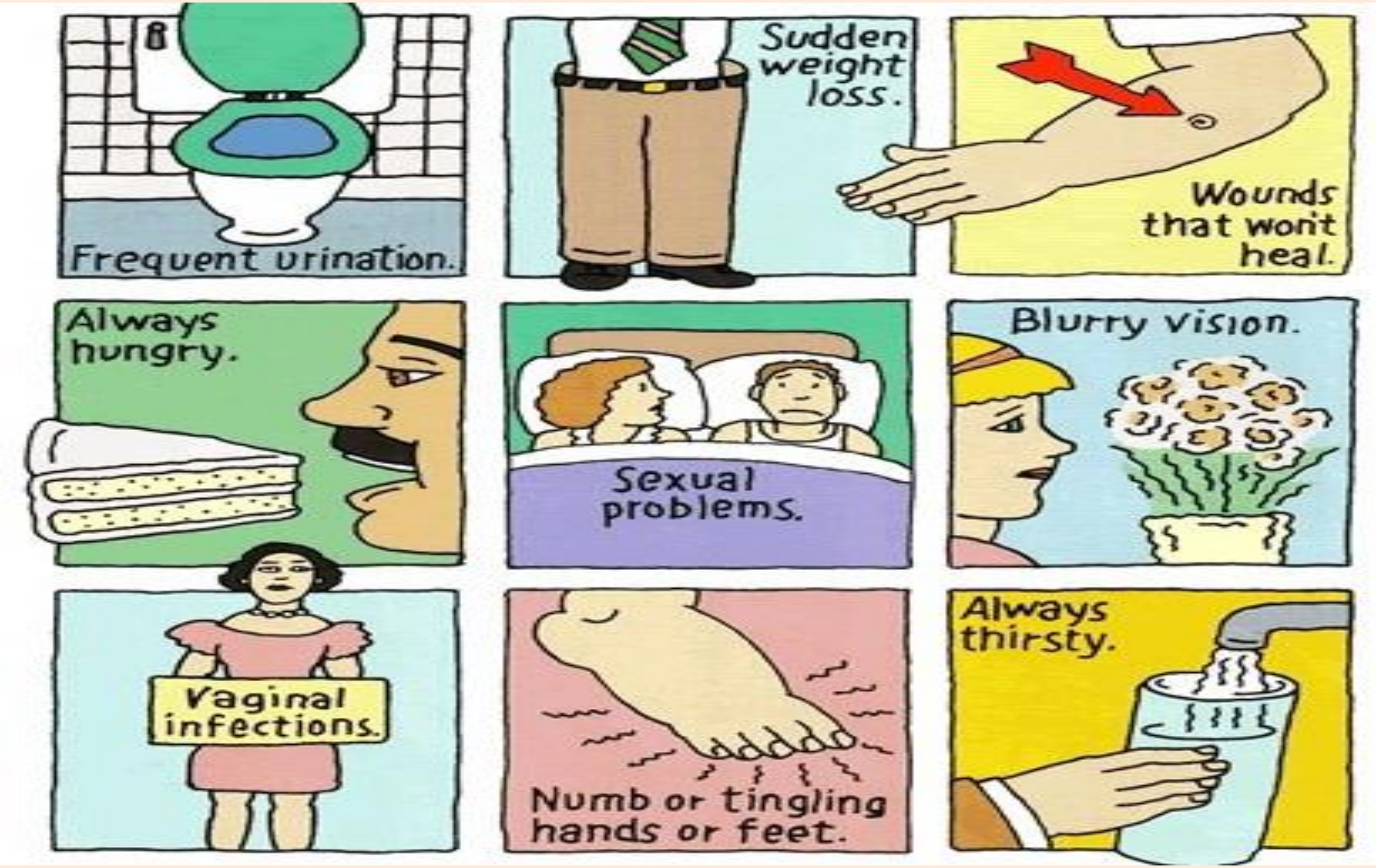

Complications of Diabetes

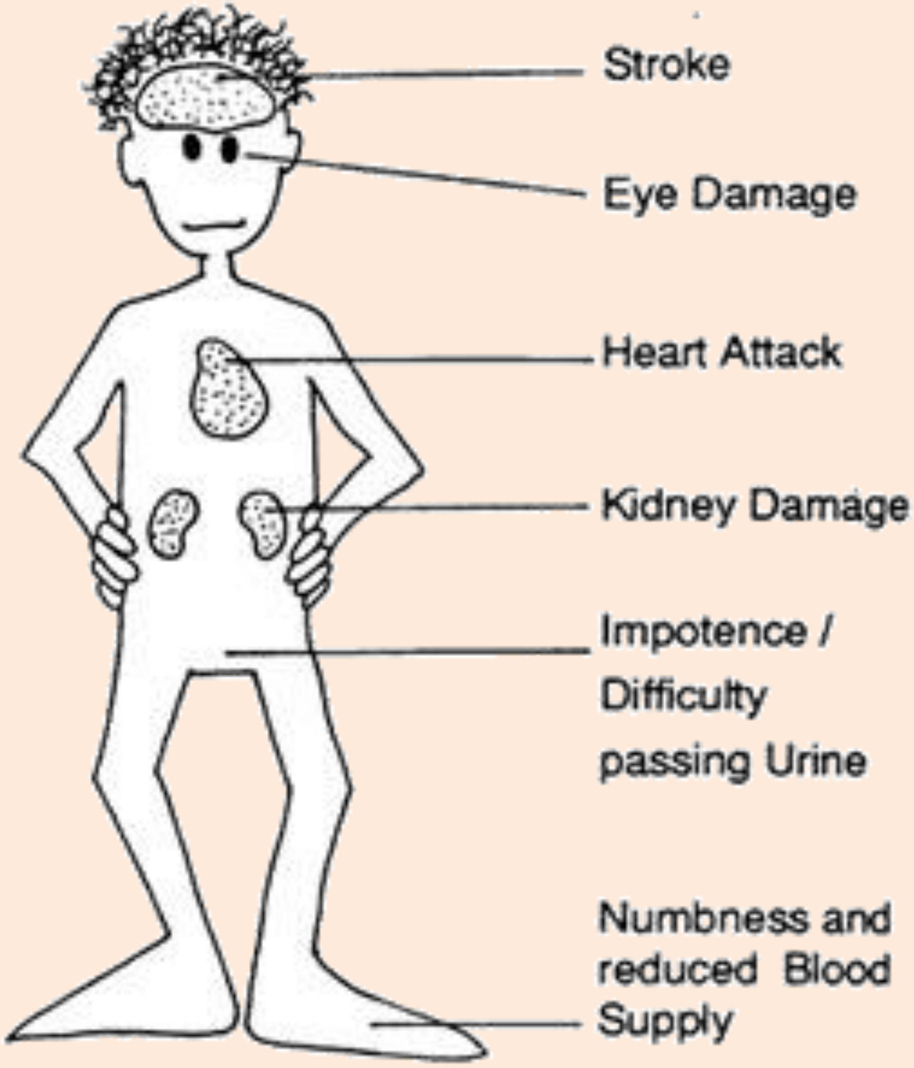

Damage to the Nerves and Blood Vessels

Diabetic Retinopathy

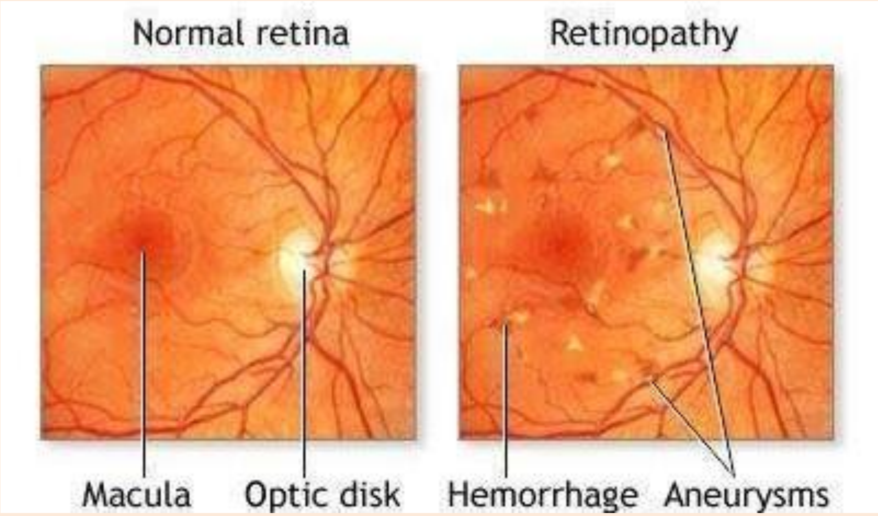

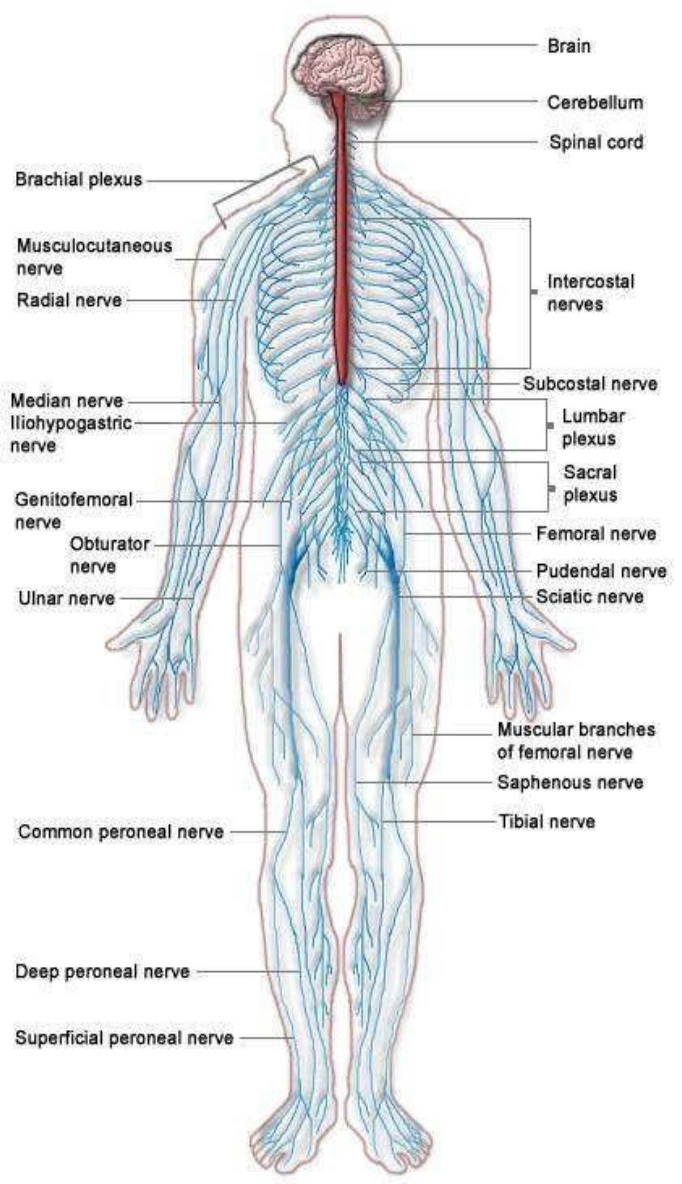

Diabetic Nephropathy

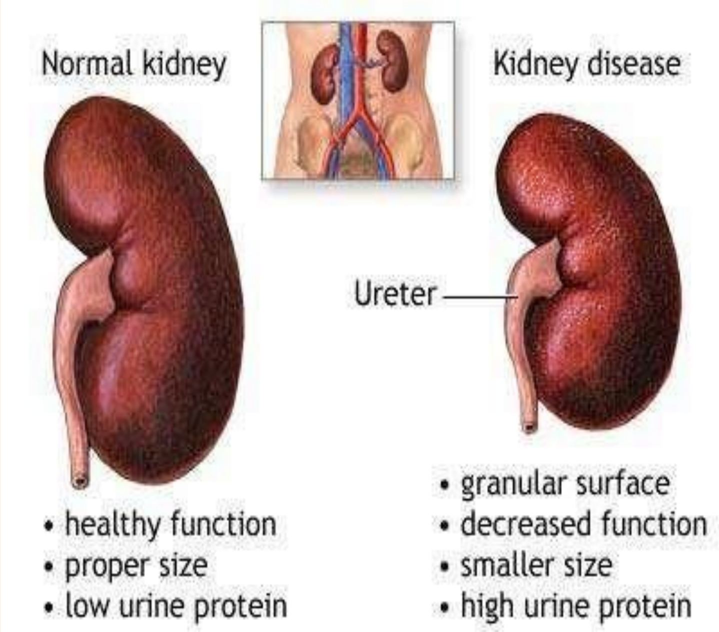

Diabetic Foot Disease

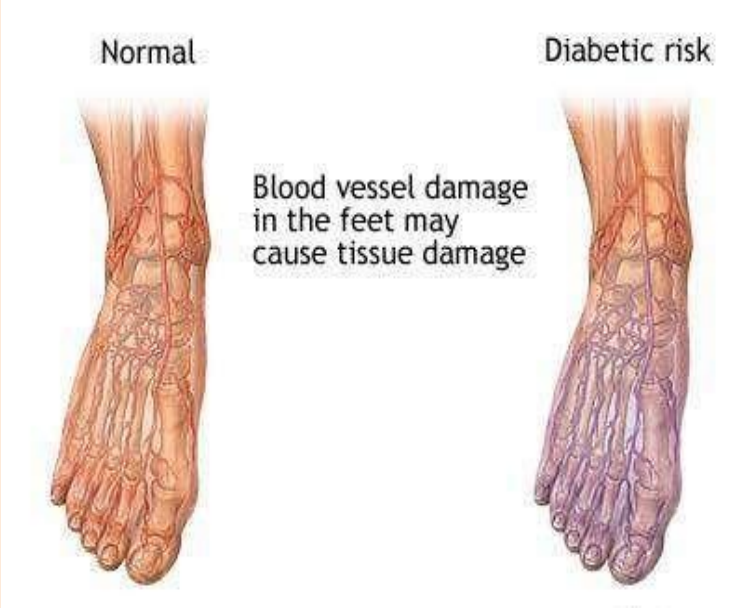

Symptoms of High Blood Sugar

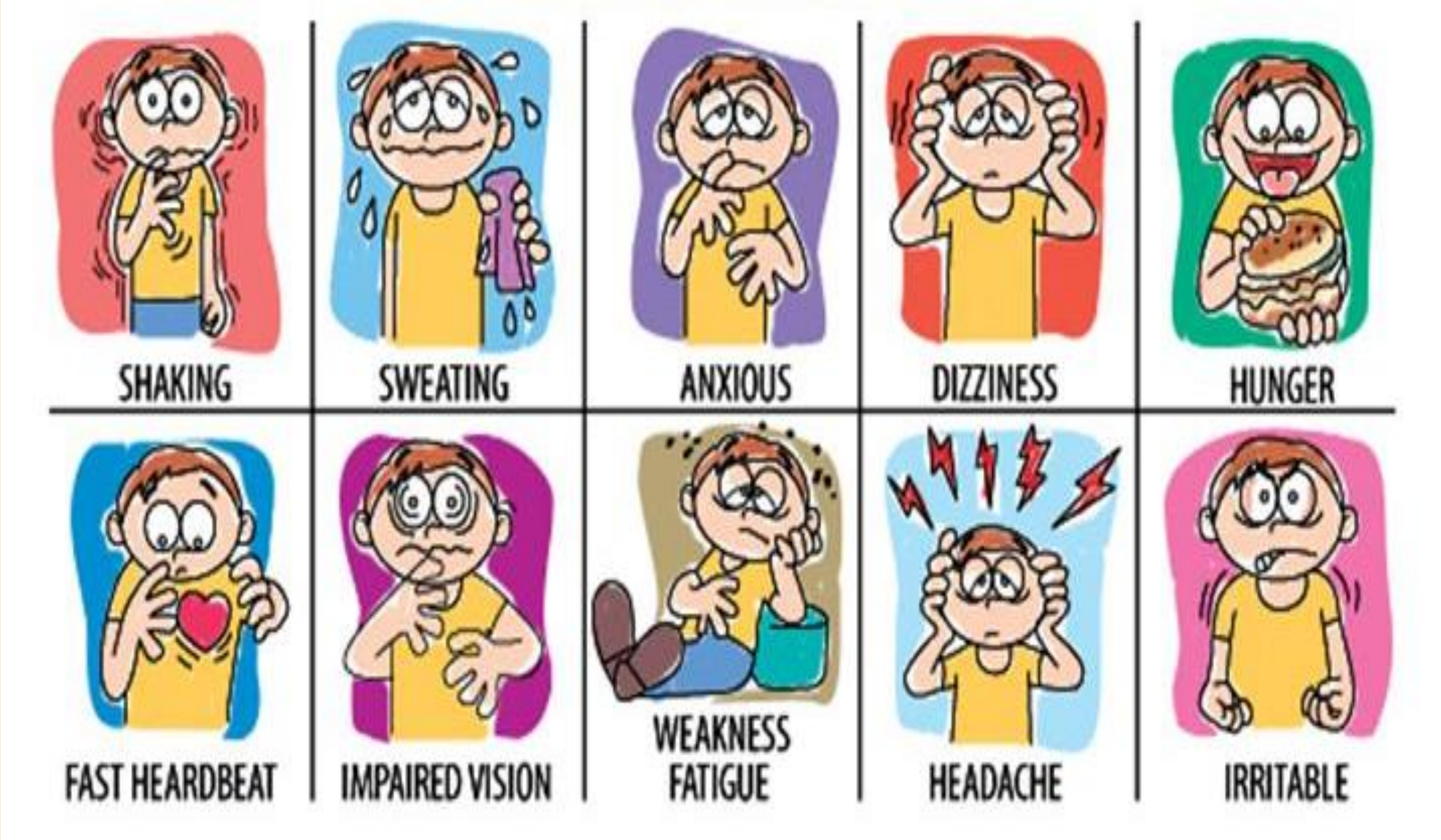

Symptoms of Hypoglycemia

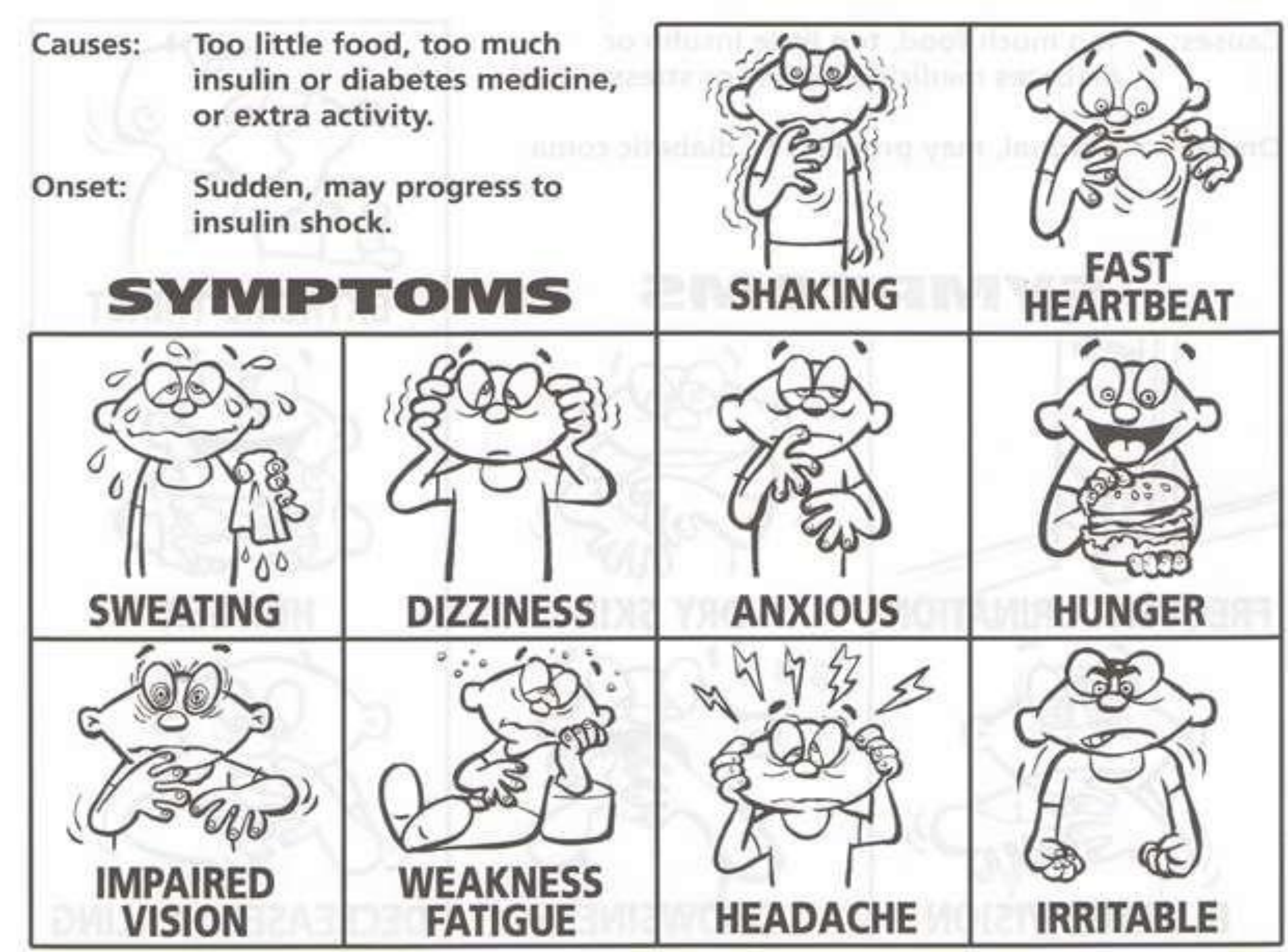

Diabetes Management

Diabetes cannot be prevented but it can be well managed by adopting certain control measures

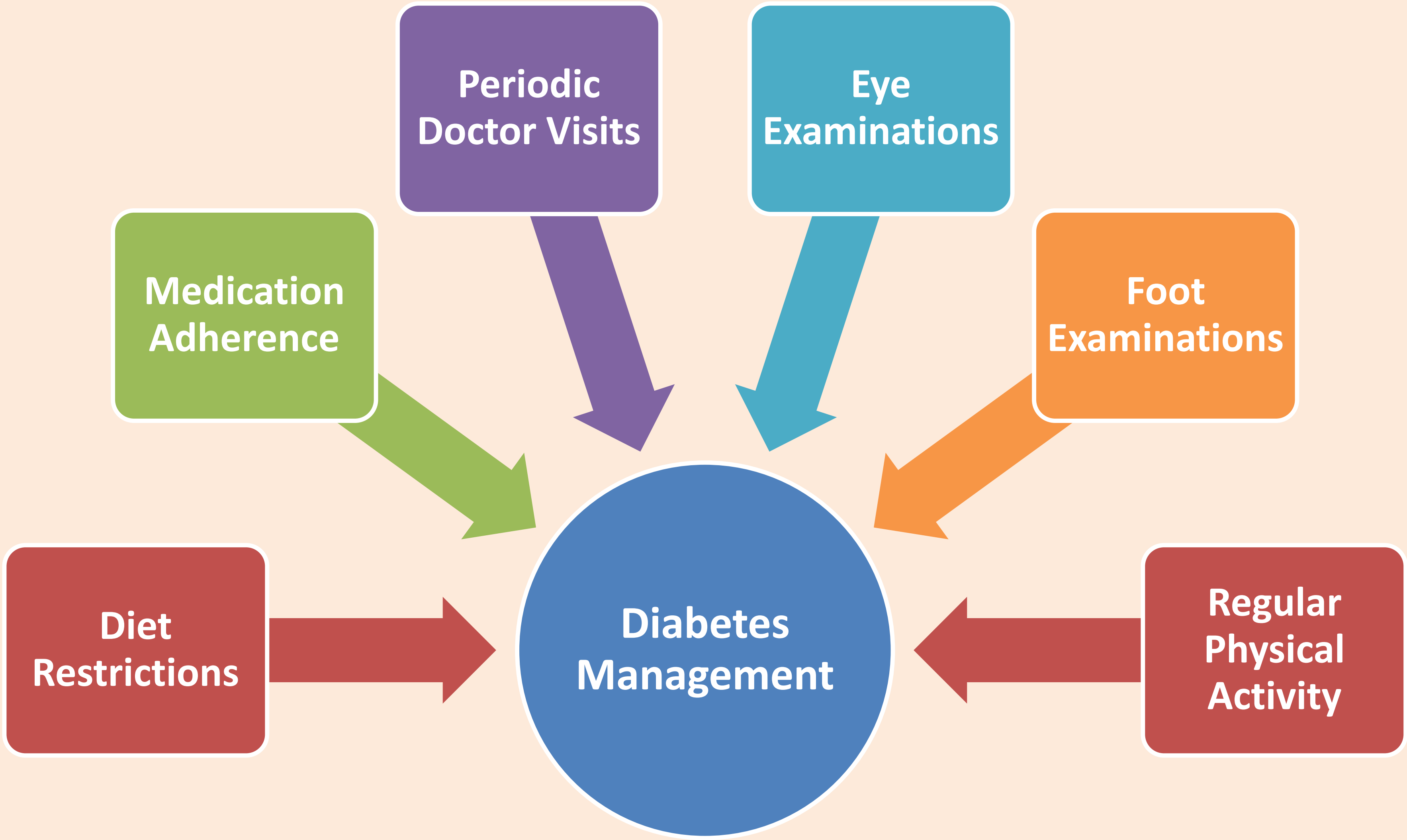

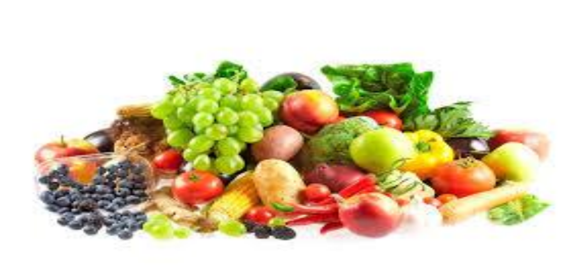

Diet

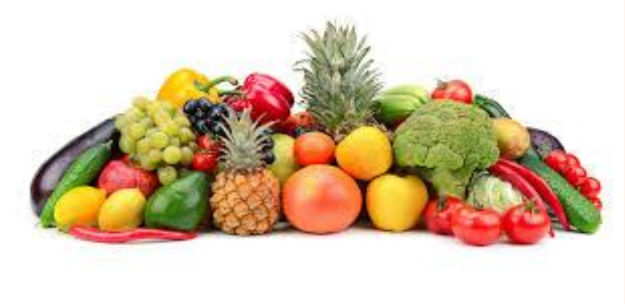

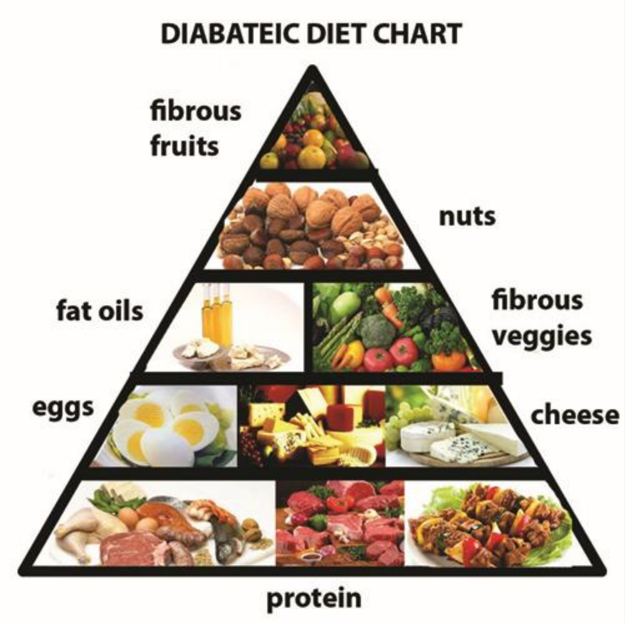

- Diabetic diet should have more salads and fresh vegetables

- Food should be consumed in smaller quantities at frequent intervals

- Bakery products and sweets should be avoided

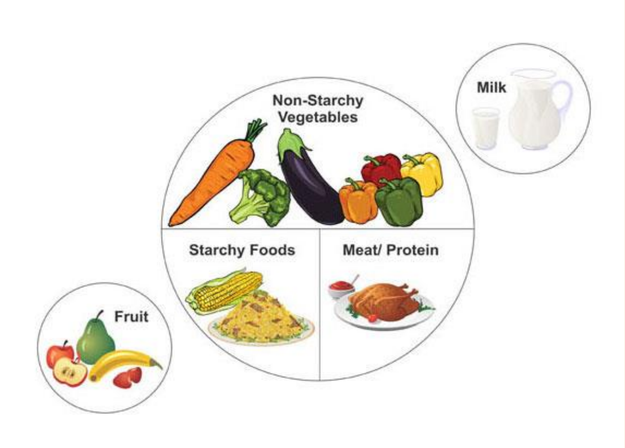

Whole grains and Legumes

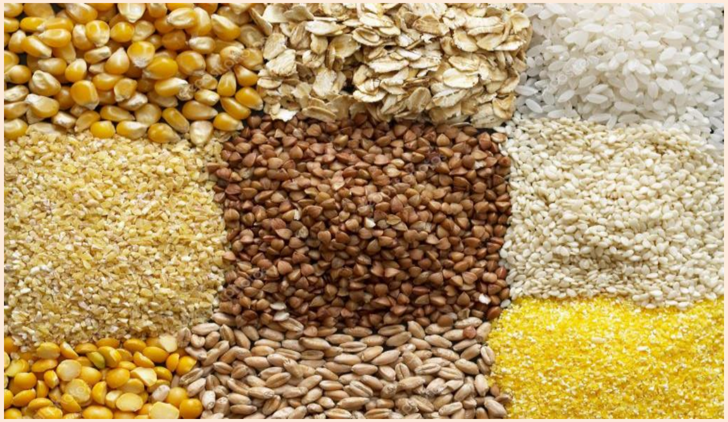

- Consumption of high carbohydrate food should be avoided.

- Ragi, Jowar, Pulses and Legumes that had good nutritious values and proteins should be consumed.

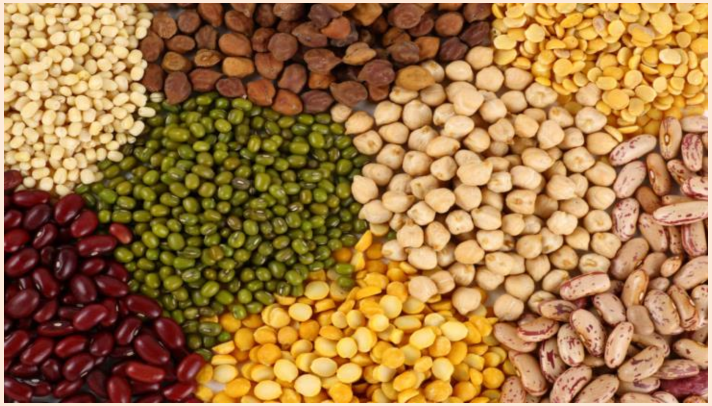

Vegetables

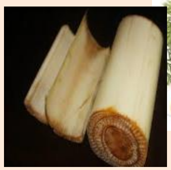
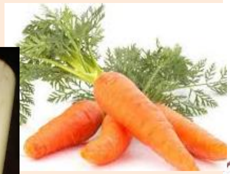
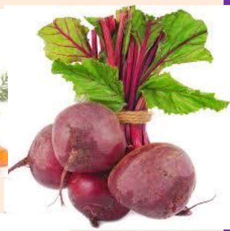

Fresh vegetables and salads can be consumed along with beans and sprouts

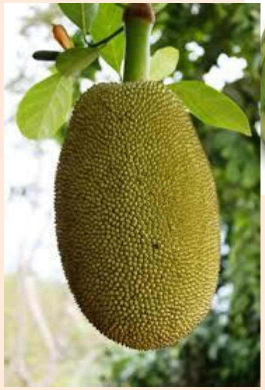
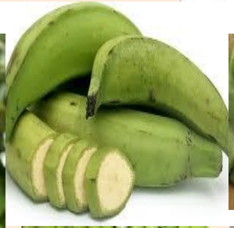
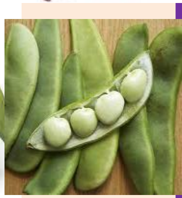
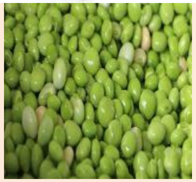
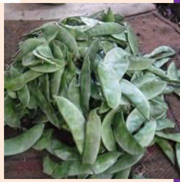

Where as sweet potato, pumpkin, potatoes should be consumed in very limited quantities as they are rich in carbohydrates

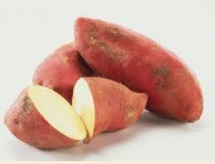
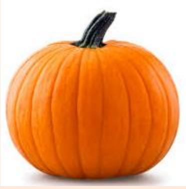
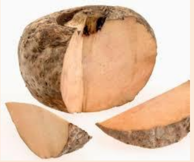
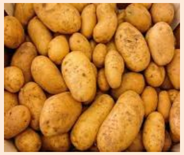

All kinds of green leafy vegetables can be consumed

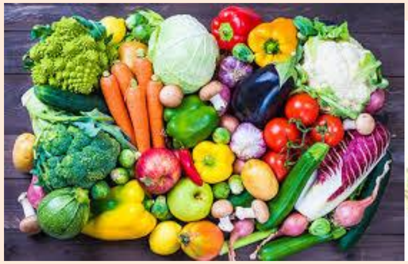
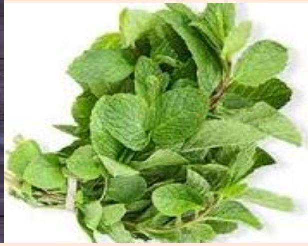
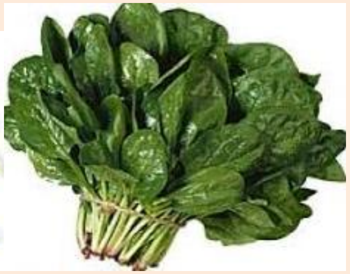
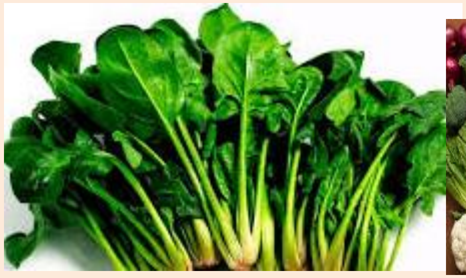
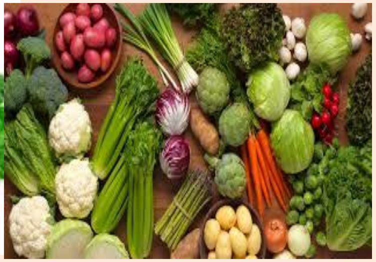

Fruits

- Fruits contain natural sugars, especially fruits such as banana
- Diabetics can consume the below mentioned fruits in smaller proportions

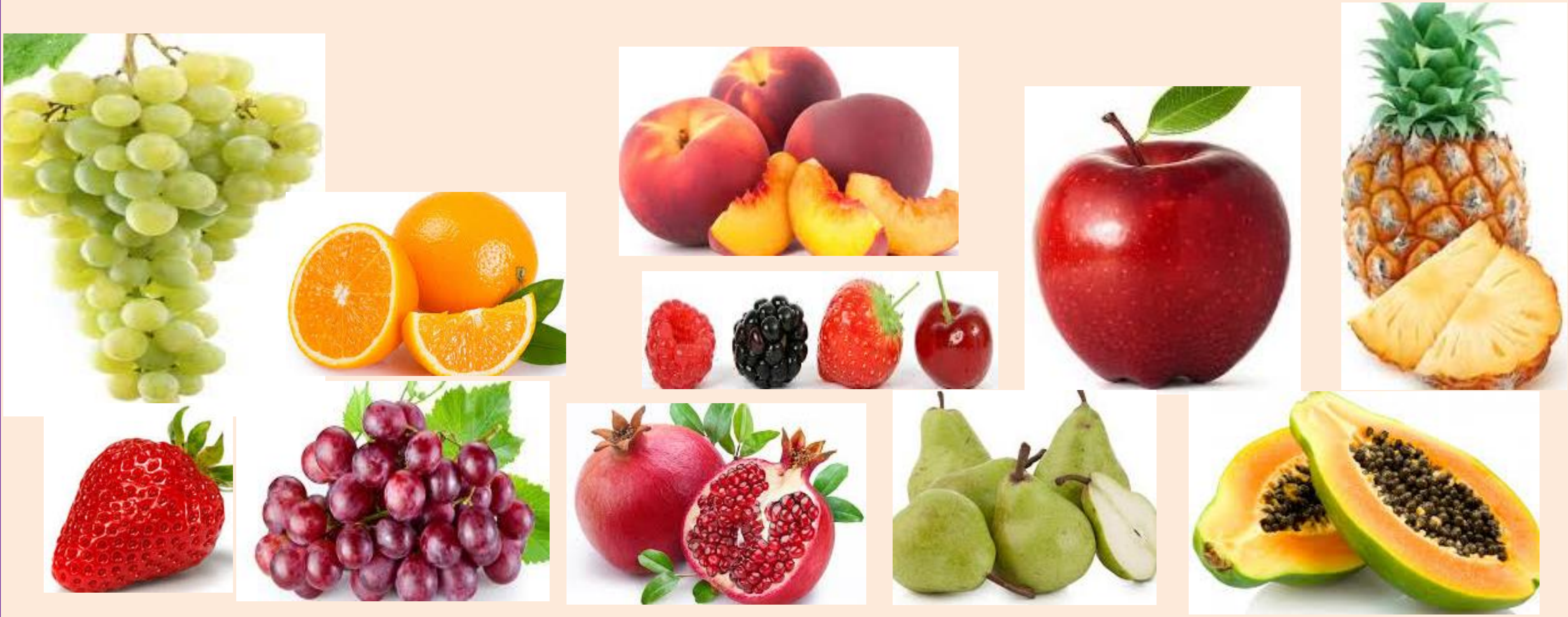

Dairy Products

Milk is a good source of protein and calcium.
Low fat milk, low fat curd and buttermilk can be consumed

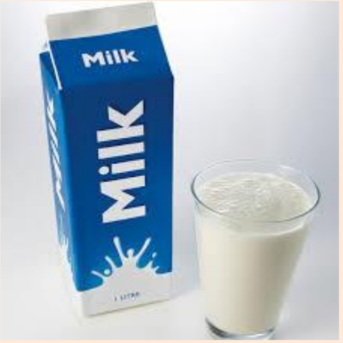

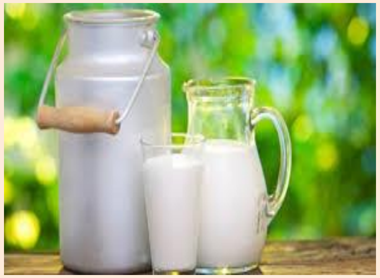

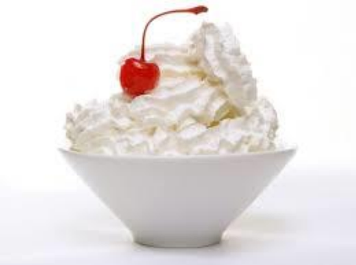

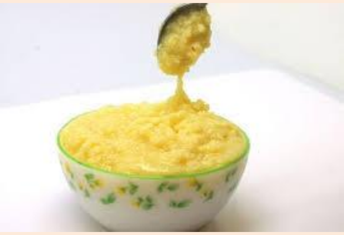

Milk products such as cream, butter, Ghee, condensed milk and paneer can be consumed in very limited quantities

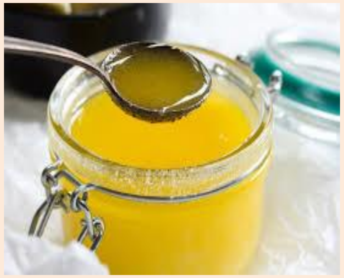

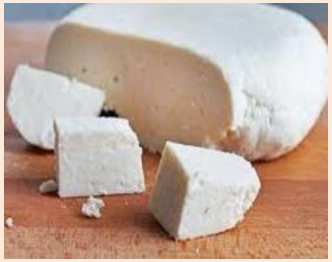

Oils

- Sun flower oil, refined oil and canola oils can be used to not more than 20 grams per day

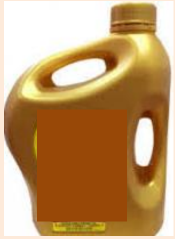
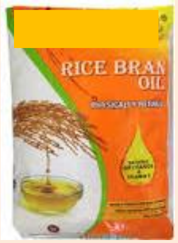

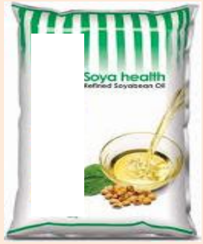

- Dalda and vanaspathi oils should be avoided

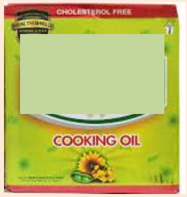

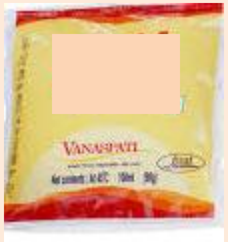

- Deep fried and processed foods should be avoided to the maximum possible

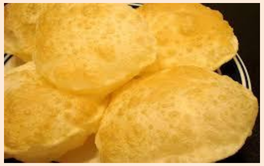
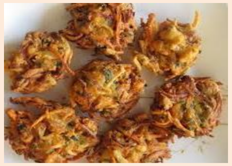
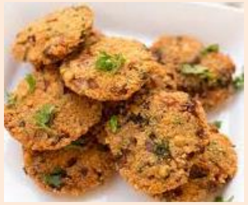
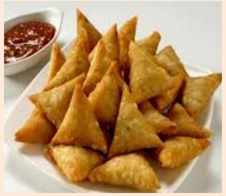
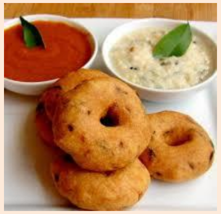

Dry fruits & nuts

Almonds, Cashew nuts, coconut and ground nuts can be consumed in smaller proportions

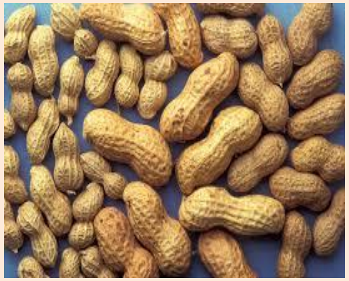

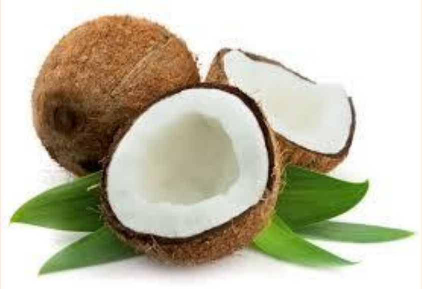

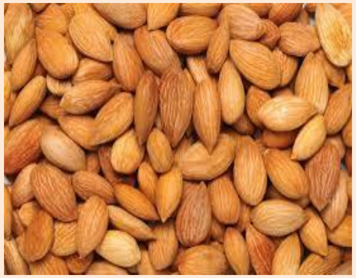

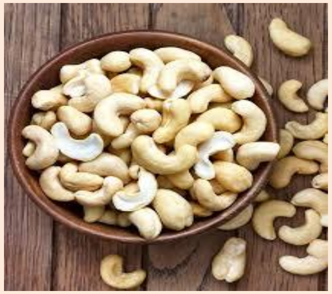

Meat products

Meat products should be consumed in very smaller proportions

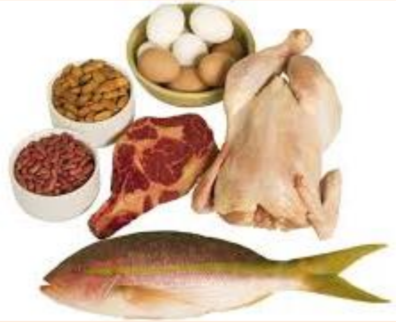

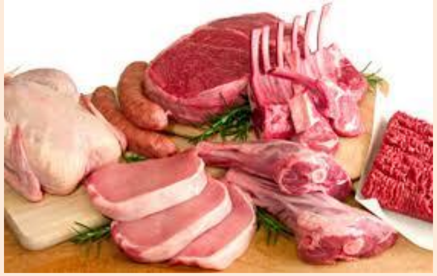

Beverages and food items that increases your blood sugar

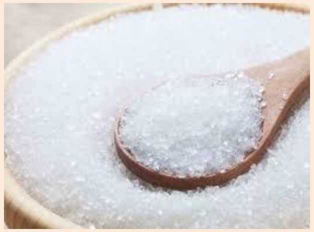

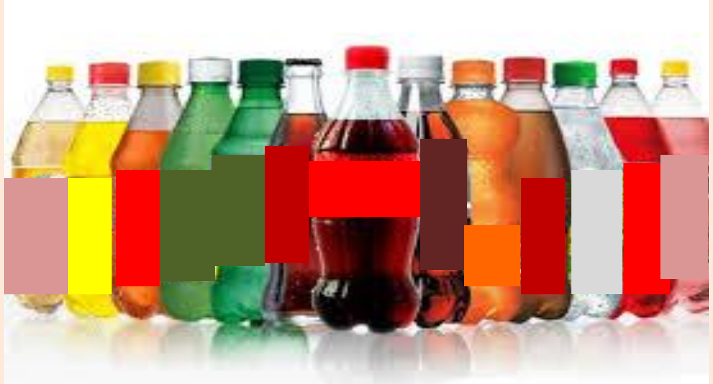

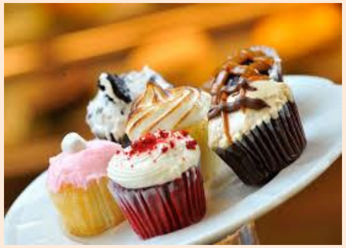

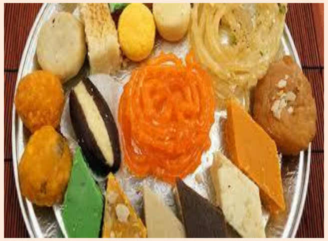

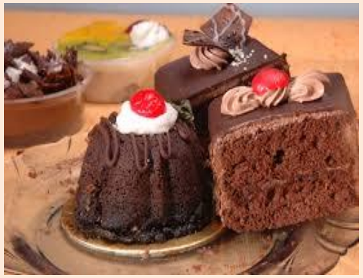

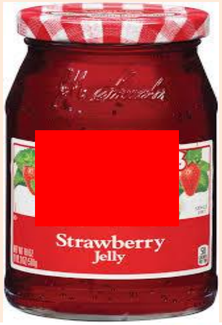

Sugar, Jelly, Jam, Powdered glucose, Sweets, Ice cream, Cool drinks, Chocolates increases blood sugar. So these foods should be avoided

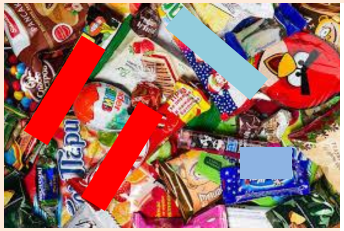

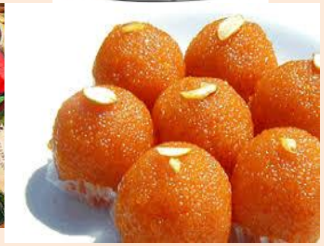

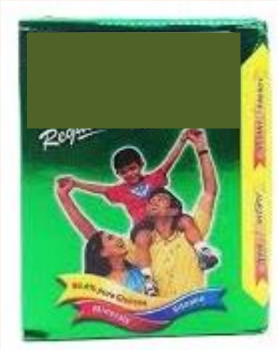

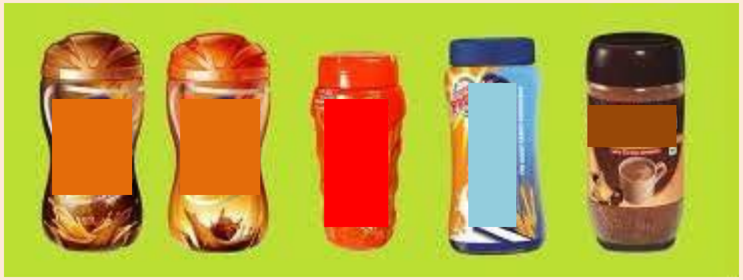

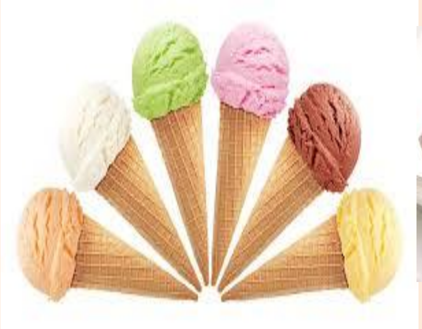

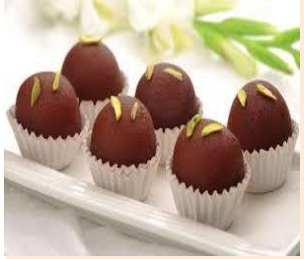

Medication Adherence and Doctor Visits

- Diabetes medicines helps in controlling the blood glucose levels

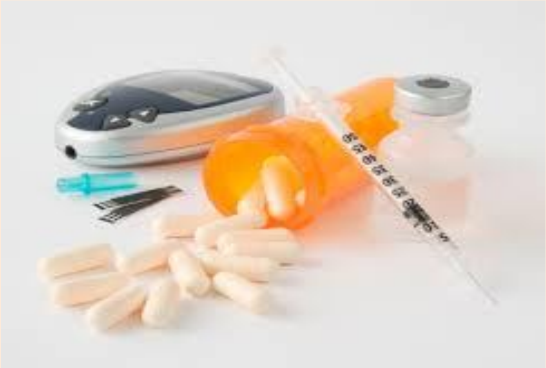

- So, Medicines should be taken as directed by the doctor at respective times

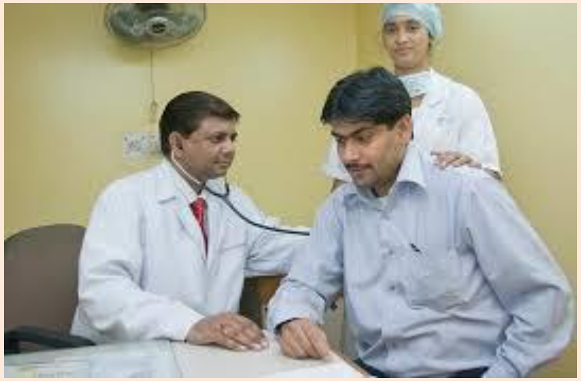

- Periodic doctor visits should be made and doctors instructions in diabetes care should be followed

Regular physical activity

- Regular exercise plays a significant role in controlling blood sugar values
- Physical activity such as walking, cycling, jogging or playing sports should be done for a minimum of 30 minutes per day without fail

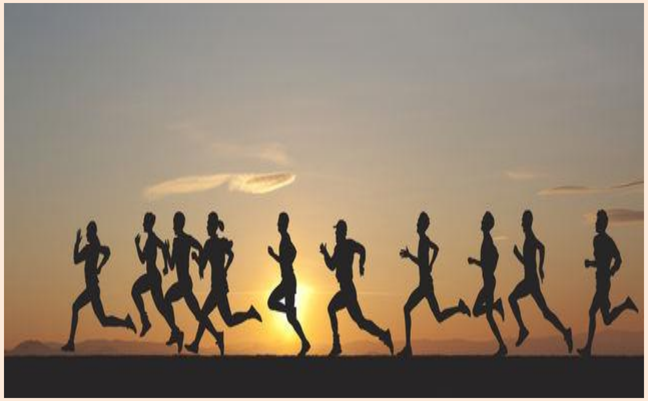
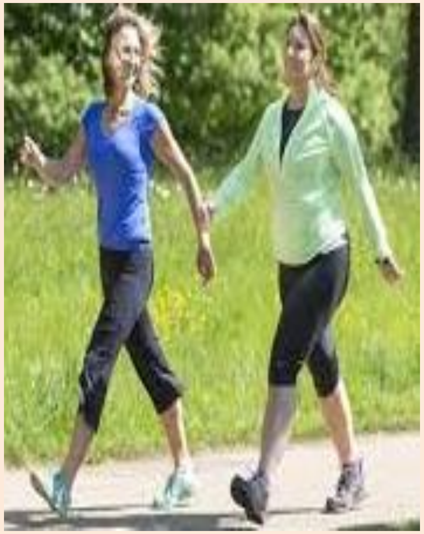
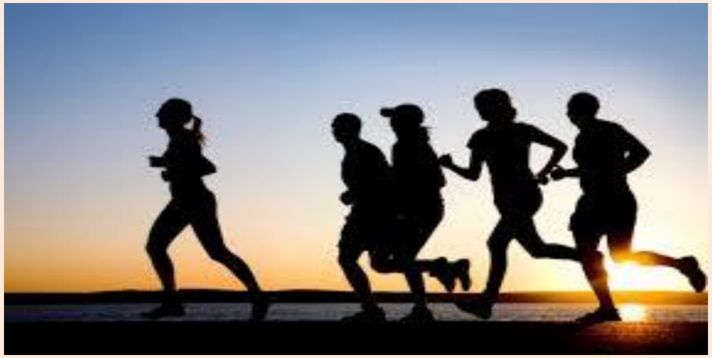
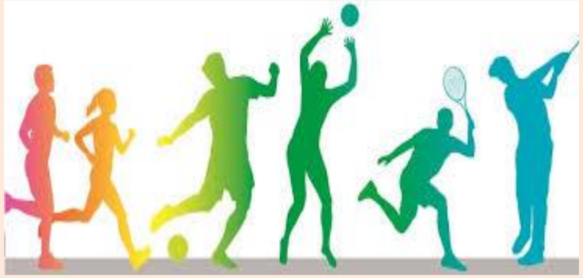

Eye Examination

Diabetic patients should be consulting ophthalmologist once in every 6 months at least for regular eye examination.

If you have any of the following

- Blurring of vision
- Itching and burning of eyes
- Poor vision

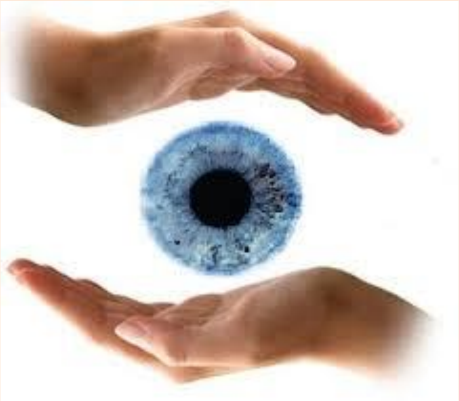

Protect your eyes

Please contact your doctor immediately

Foot Examination

- Check your feet every day
- Look for cuts, blisters, red spots, swelling
- Wash your feet every day in warm (not hot) water
- Keep your feet soft and smooth
- Trim toenails weekly
- Never walk barefoot

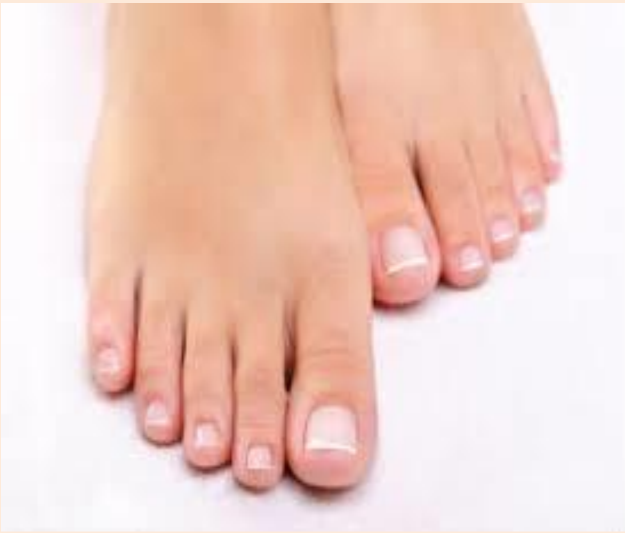

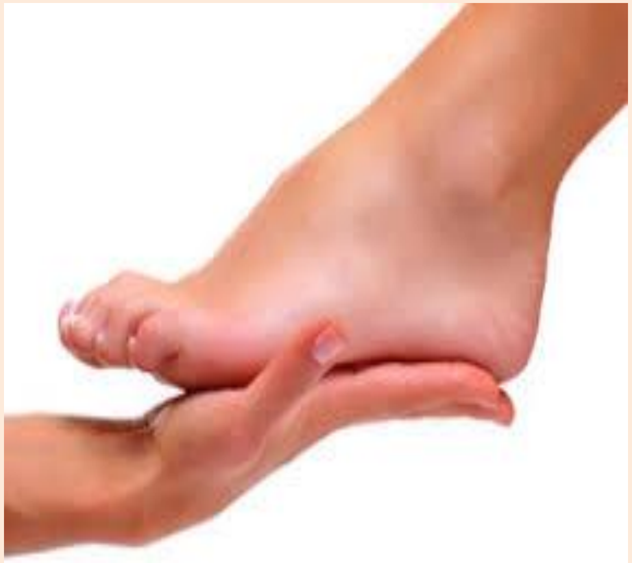

Know your blood sugar values

- Get your HbA1C tested once in every 3 months.
- HbA1c investigation helps in identifying the average blood glucose levels in your body for past 3 months.
- Diabetes can be better managed if periodic HbA1c levels are known.

Normal Blood Glucose: 80-120 mg/dl

HbA1C< 7

Diabetes Associated Complications

Uncontrolled diabetes could lead to the following complications

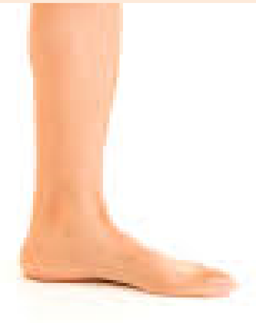

Foot Ulcers

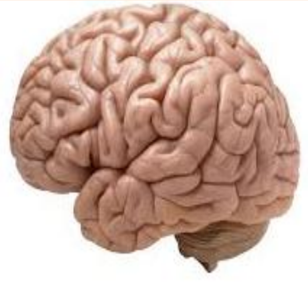

Stroke

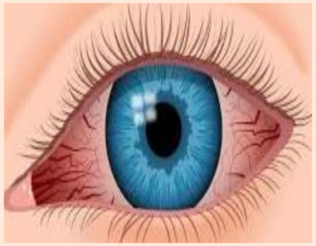

Eye problems

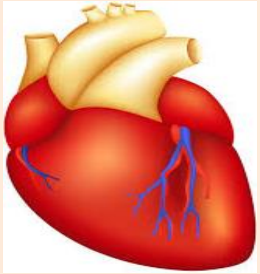

Heart Diseases

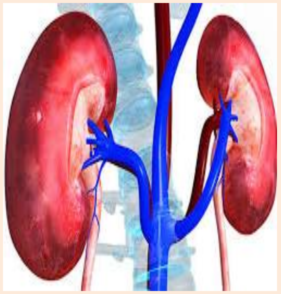

Kidney diseases
