# Supplementary material for: Intervention through Short Messaging System (SMS) and phone call alerts reduced HbA1C levels in ~47% type-2 diabetics–results of a pilot study
Source: PLoS One. 2020 Nov 17;15(11):e0241830. doi: 10.1371/journal.pone.0241830 (PMC7671489; doi:10.1371/journal.pone.0241830)
Supplement: S5 File — Diabetes orientation/reorientation educational material was presented in English and local language Kannada. (PDF) [file pone.0241830.s005.pdf]

JSS ACADEMY OF HIGHER EDUCATION & RESEARCH
(DEEMED TO BE UNIVERSITY) MYSURU

JSS HOSPITAL
IN SERVICE TO LIFE

ಮಧುಮೇಹ ನಿರ್ವಹಣೆ

JSS Academy of Higher Education and Research
JSS Medical Institutions Campus
Sri Shivarathreeshwara Nagara
Mysuru – 570 015, Karnataka, INDIA
Phone: +91-821-2548416 / 400 **Fax:** +91-821-2548394

ಮಧುಮೇಹ

- ನಿಮ್ಮ ರಕ್ತದಲ್ಲಿ ಗ್ಲುಕೋಸ್ ಅಂಶ ಅಥವಾ ಸಕ್ಕರೆಯ ಅಂಶವು ಅತಿಯಾಗಿ ಇದ್ದ ಪಕ್ಷದಲ್ಲಿ ನಿಮ್ಮನ್ನು ಮಧುಮೇಹ ರೋಗಿಗಳೆಂದು ಗುರುತಿಸುತ್ತಾರೆ.
- ಇನ್ಸುಲಿನ್, ಒಂದು ಹಾರ್ಮೋನ್ ಆಗಿದ್ದು, ಅದು ಮೇದೋಜೀರಕ ಗ್ರಂಥಿಯಿಂದ ಉತ್ಪನ್ನವಾಗುತ್ತದೆ ಮತ್ತು ಆಹಾರದಿಂದ ಗ್ಲುಕೋಸ್‌ನ್ನು ಜೀವಕೋಶಗಳಿಗೆ ಕಳುಹಿಸಿ ಶಕ್ತಿಯನ್ನು ಉತ್ಪಾದಿಸುತ್ತದೆ.
- ಕೆಲವೊಮ್ಮೆ ನಿಮ್ಮ ದೇಹವೂ ಇನ್ಸುಲಿನ್ನು ಉತ್ಪಾದಿಸುತ್ತದೆ ಮತ್ತೆ ಕೆಲವೊಮ್ಮೆ ಉತ್ಪಾದಿಸುವುದಿಲ್ಲ.
- ಹಾಗಾಗಿ, ಗ್ಲುಕೋಸ್ ನಿಮ್ಮ ರಕ್ತದಲ್ಲಿಯೇ ಉಳಿದು ಜೀವಕೋಶಗಳನ್ನು ತಲುಪದ ಕಾರಣ, ರಕ್ತದಲ್ಲಿ ಗ್ಲುಕೋಸ್ನ ಪ್ರಮಾಣ ಅತ್ಯಧಿಕವಾಗಿದ್ದು, ದೈಹಿಕವಾಗಿ ಹಲವಾರು ಸಮಸ್ಯೆಗಳಿಗೆ ಎಡೆಮಾಡಿಕೊಡುತ್ತದೆ.

ಮಧುಮೇಹ

ಮೊದಲನೇ ವಿಧ

ದೇಹದಿಂದ ಇನ್ಸುಲಿನ್
ಉತ್ಪಾದನೆಯಾಗುವುದಿಲ್ಲ.

30 ವರ್ಷದೊಳಗೆ ಕಾಣಿಸಿಕೊಳ್ಳುವುದು.

ಎರಡನೇ ವಿಧ

ದೇಹವು ಇನ್ಸುಲಿನ್‌ನ್ನು ಬೇಕಾಗುವಷ್ಟು ಉತ್ಪಾದಿಸುವುದಿಲ್ಲ. ಸಾಮಾನ್ಯವಾಗಿ 40 ವರ್ಷದ ಮೇಲ್ಪಟ್ಟವರಲ್ಲಿ ಈ ಸಮಸ್ಯೆ ಕಾಣಿಸಿಕೊಳ್ಳುತ್ತದೆ.ಶೇಕಡ 85 ರಿಂದ 90%, ಈ ವಿಧದ ಸಮಸ್ಯೆಯು

ಸಾಮಾನ್ಯವಾಗಿದೆ

ಮೂರನೇ ವಿಧ

ಗರ್ಭಿಣಿಯರಲ್ಲಿ

ಕಾಣಿಸಿಕೊಳ್ಳುವುದು

ಮಧುಮೇಹದ ಚಿಹ್ನೆಗಳು

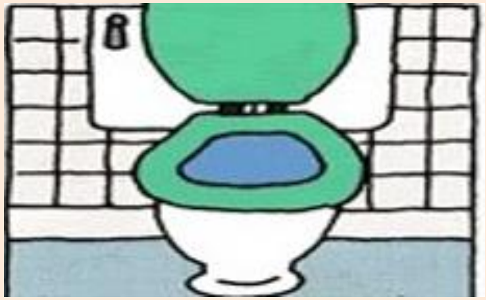

ಪದೇಪದೇ ಮೂತ್ರ ವಿಸರ್ಜನೆ

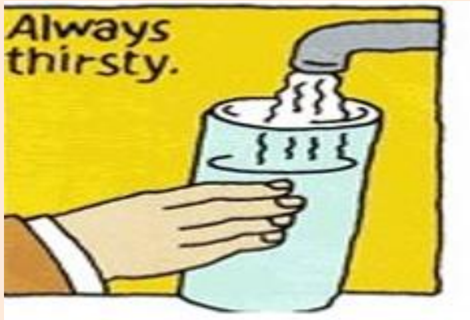

ಯಾವಾಗಲೂ ಬಾಯಾರಿಕೆ

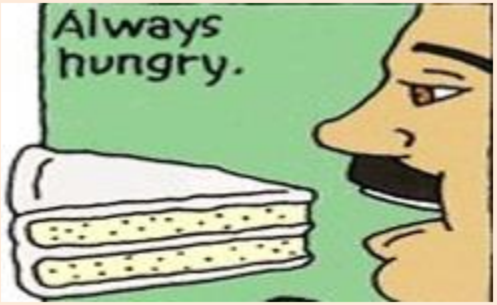

ಯಾವಾಗಲು ಹಸಿವಿರುವುದು

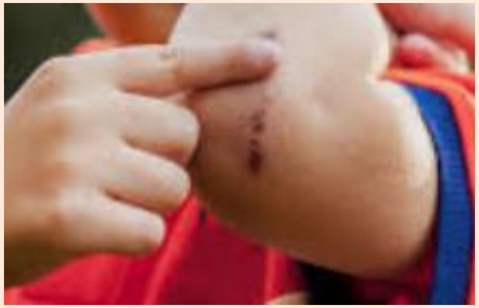

ಗಾಯಗಳು ಬೇಗ ವಾಸಿಯಾಗುವುದಿಲ್ಲ

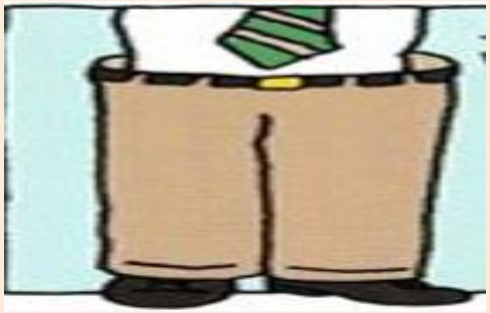

ಹರಾತ್ತಾಗಿ ತೂಕ ಕಳೆದುಕೊಳ್ಳುವಿಕೆ

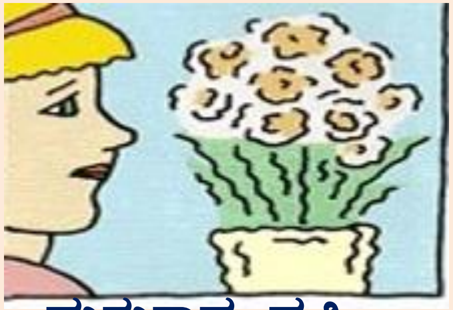

ಮಸುಕಾದ ದೃಷ್ಟಿ

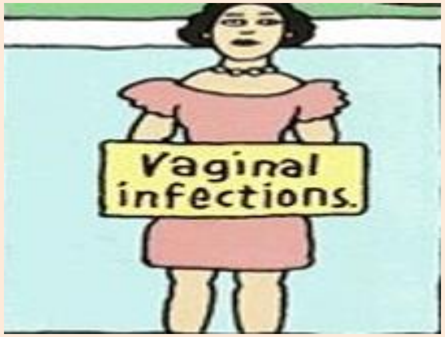

ಯೋನಿಯ ಸೋಂಕು

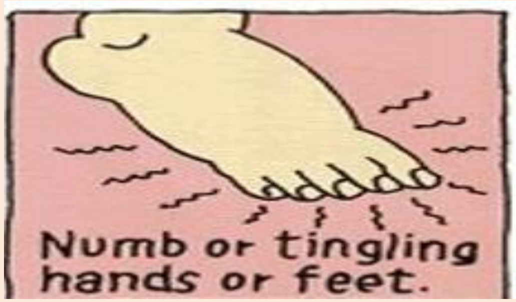

ಕೈಕಾಲು ಬೆರಳುಗಳ ಊತ

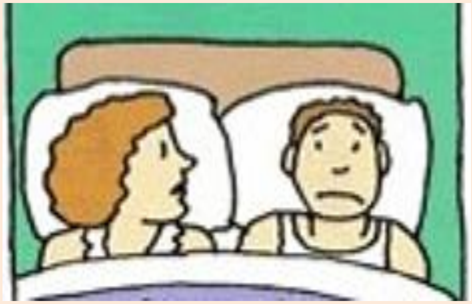

ಲೈಂಗಿಕ ಸವಸ್ಯೆಗಳು

ಮಧುಮೇಹ ರೋಗದ ಉಲ್ಬಣದಿಂದಾಗುವ ತೀವ್ರತರವಾದ ಸಮಸ್ಯೆಗಳು

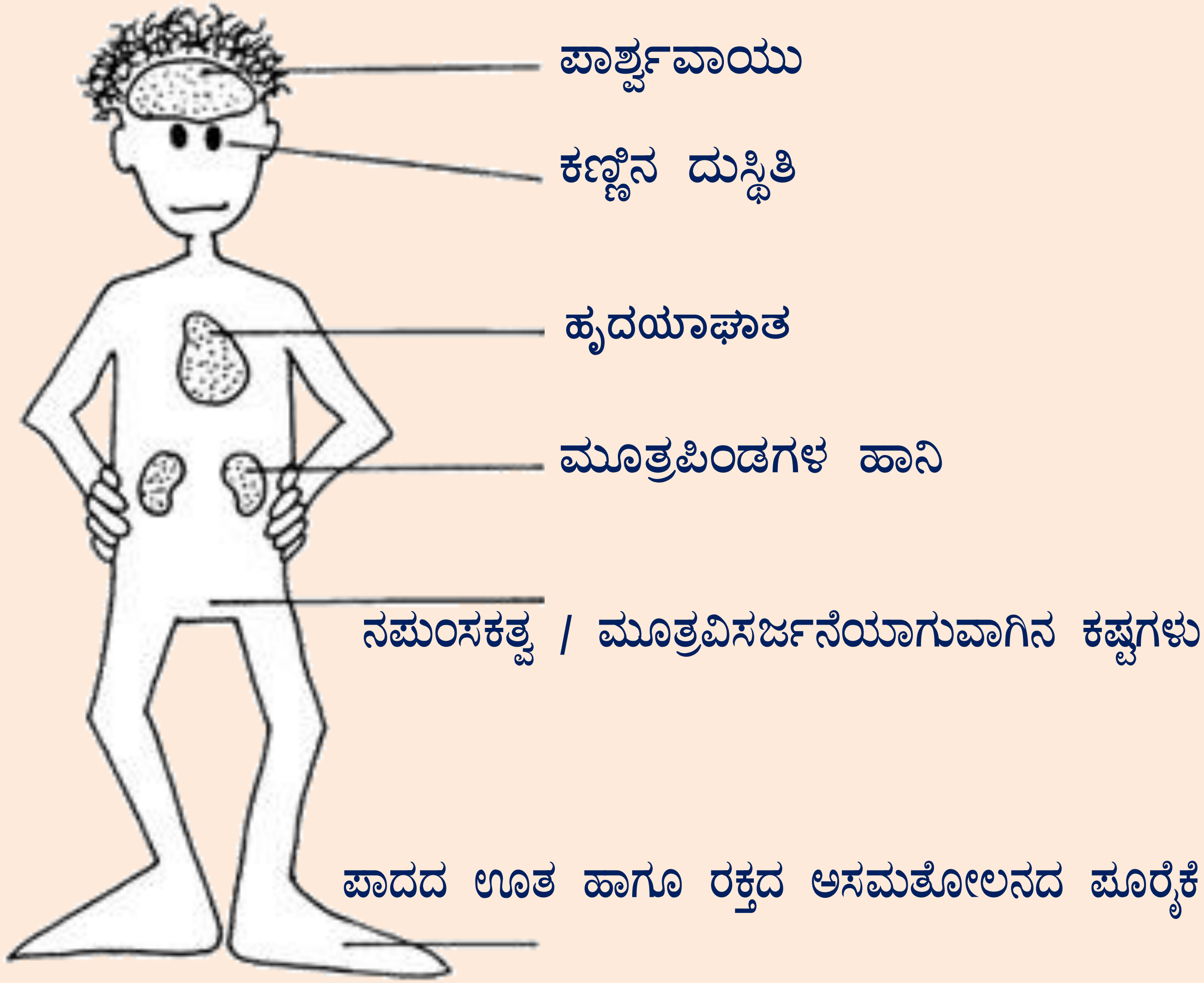

ಮಧುಮೇಹದ ರೆಟಿನೋಪತಿ

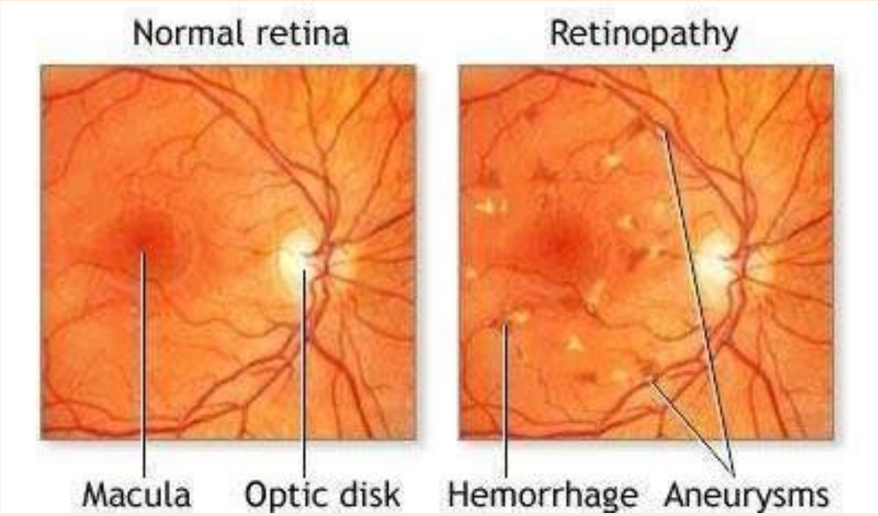

ನರ ಮತ್ತು ರಕ್ತನಾಳಗಳ ಸಮಸ್ಯೆ

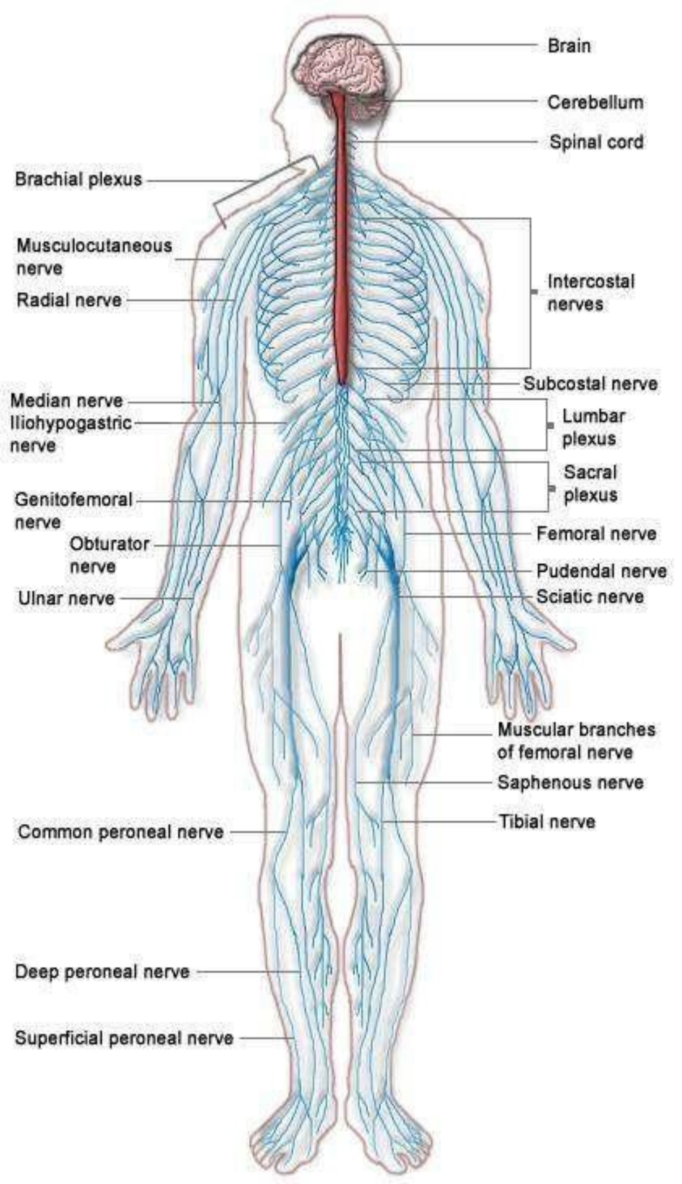

ಮಧುಮೇಹ ರೋಗದಲ್ಲಿನ ಮೂತ್ರ ಜನಕಾಂಗದ ಸ್ಥಿತಿ

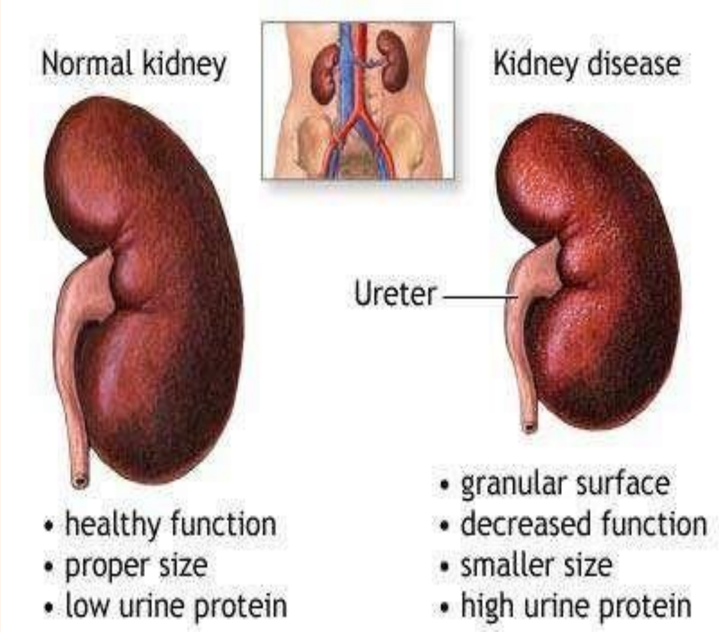

ಮಧುಮೇಹದಿಂದಾಗುವ ಪಾದಗಳ ಸಮಸ್ಯೆ

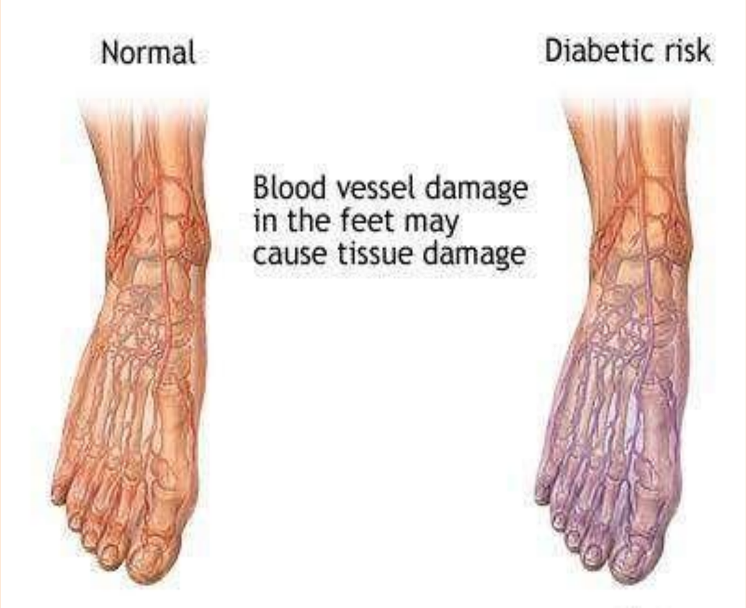

ತೀವ್ರತರವಾದ ಮಧುಮೇಹ ರೋಗದ ಚಿಹ್ನೆಗಳು

ನಡುಕ   ಬೆವರುವಿಕೆ   ಗಾಬರಿಯಾಗುವುದು   ತಲೆಸುತ್ತುವುದು   ಹಸಿವಾಗುವುದು

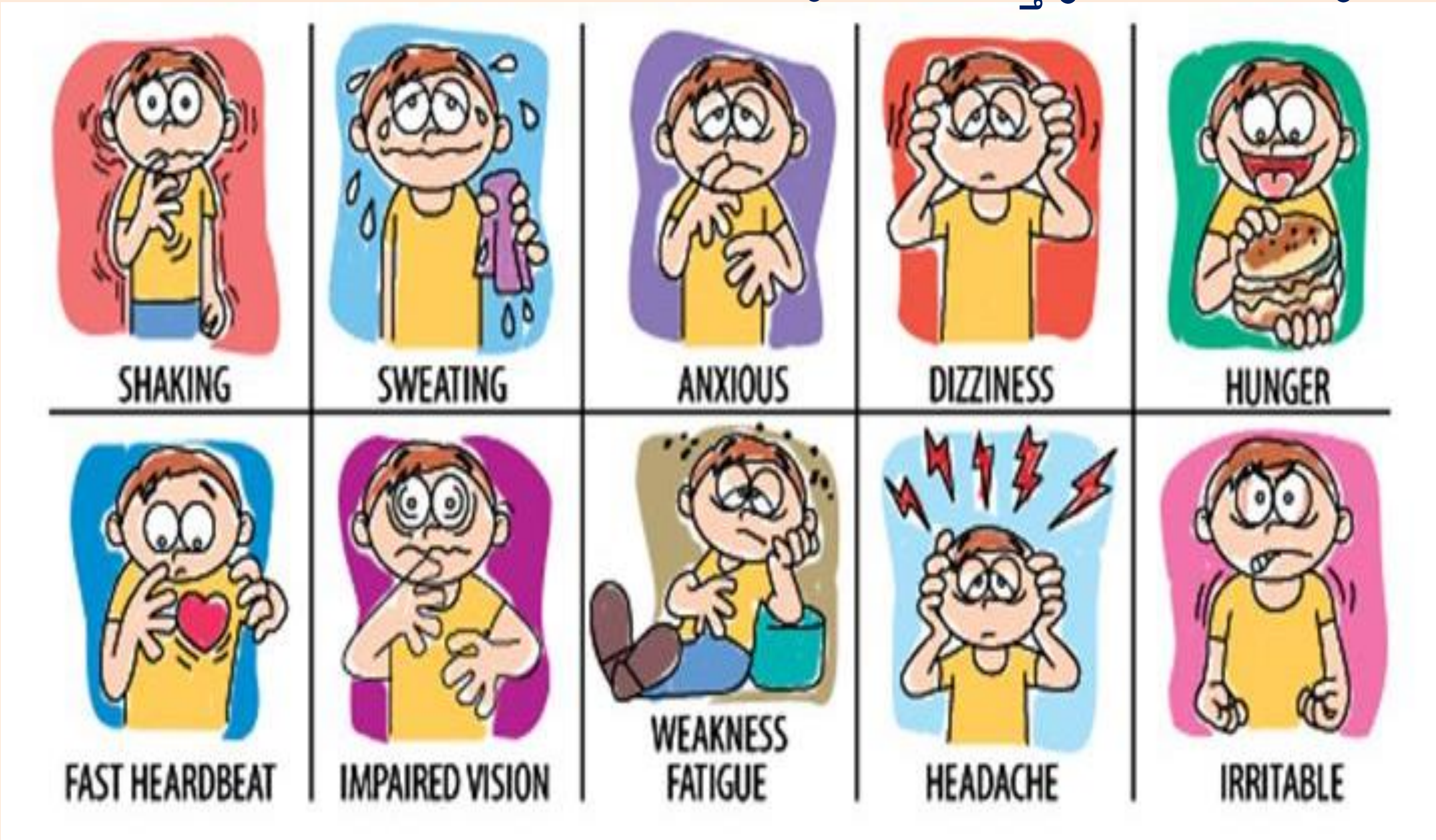

ಹೃದಯದ ಬಡಿತ ತೀವ್ರವಾಗುವುದು   ದೃಷ್ಟಿಯು ಮಸುಕಾಗುವುದು   ನಿಶ್ಯಕ್ತಿಯಿಂದ ಸುಸ್ತಾಗುವಿಕೆ   ತಲೆನೋವು   ಕಿರಿಕಿರಿಯಾಗುವುದು

ಹೈಪೋಗ್ಲೇಸಿಮಿಯಾದ ಚಿಹ್ನೆಗಳು

ಕಾರಣಗಳು : ಕಡಿಮೆ ಆಹಾರ ಸೇವನೆ, ಹೆಚ್ಚಿನ ಇನ್ಸುಲಿನ್ ಸೇರ್ಪಡೆ, ಅಥವಾ ಮಧುಮೇಹ ಔಷದಿಯ ಹೆಚ್ಚುವರಿ ಸೇರ್ಪಡೆ ಅಥವಾ ಇನ್ನಿತರ ಚಟುವಟಿಕೆ.

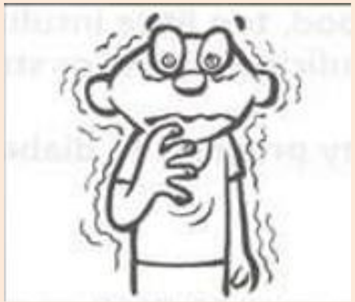

ನಡುಕ

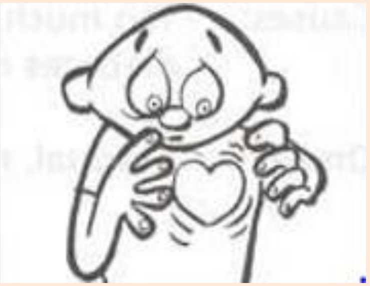

ತೀವ್ರ ಹೃದಯ ಬಡಿತ

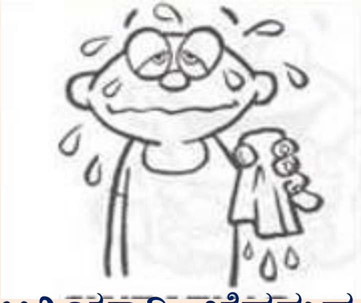

ಅತಿಯಾಗಿ ಬೆವರುವುದು

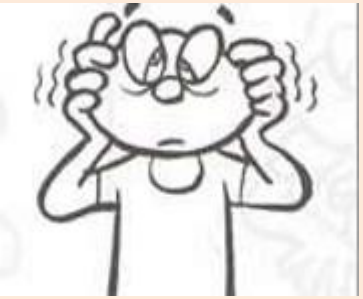

ತಲೆಸುತ್ತುವುದು

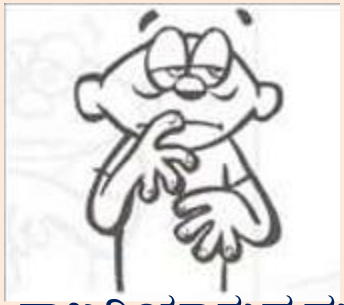

ಗಾಬರಿಯಾಗುವುದು

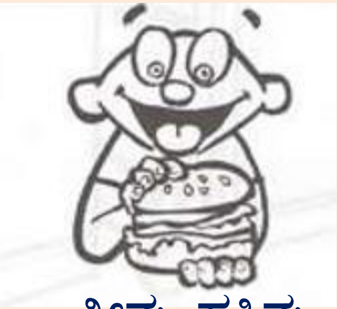

ತೀವು ಹಸಿವು

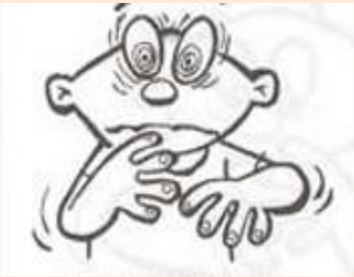

ದೃಷ್ಟಿಯು ಮಸುಕಾಗುವುದು

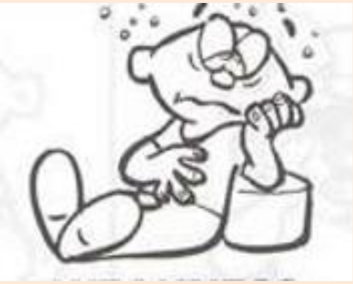

ನಿಶ್ಯಕ್ತಿಯ ಬಳಲಿಕೆ

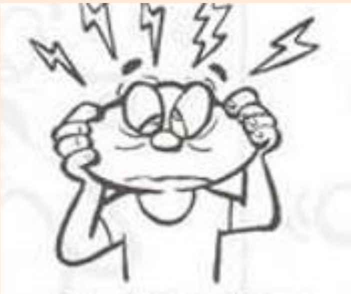

ತಲೆನೋವು

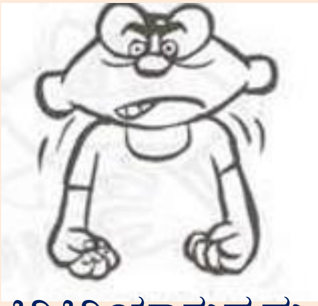

ಕಿರಿಕಿರಿಯಾಗುವುದು

ಮಧುಮೇಹದ ನಿರ್ವಹಣೆ

ಮಧುಮೇಹನ್ನು ಸಂಪೂರ್ಣವಾಗಿ ಹೋಗಲಾಡಿಸಲು ಅಸಾಧ್ಯವಾದರೂ ಉತ್ತಮ ನಿರ್ವಹಣೆಗಾಗಿ ಅನುಸರಿಸಬೇಕಾದ ಮುಂಜಾಗರೂಕತೆಗಳು

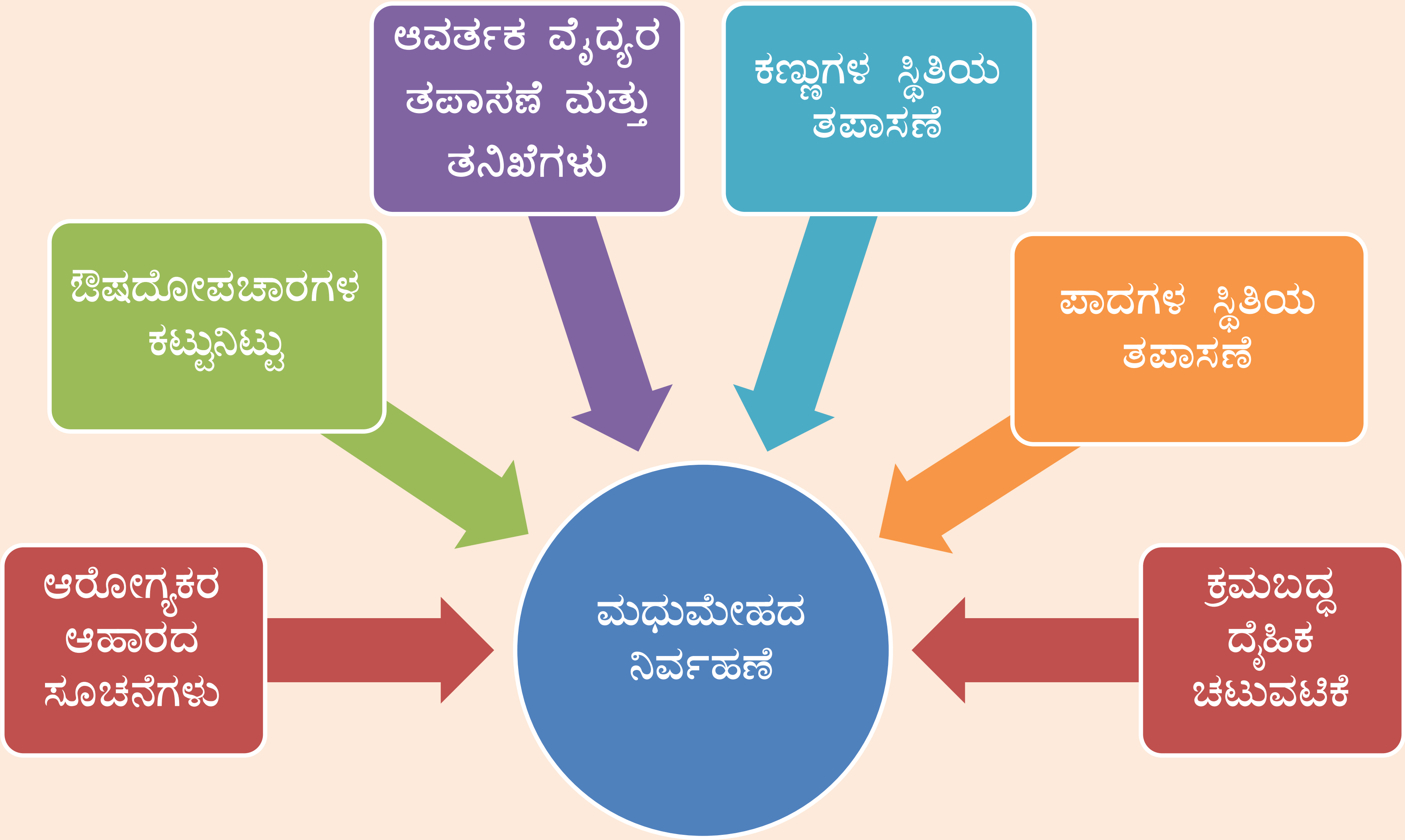

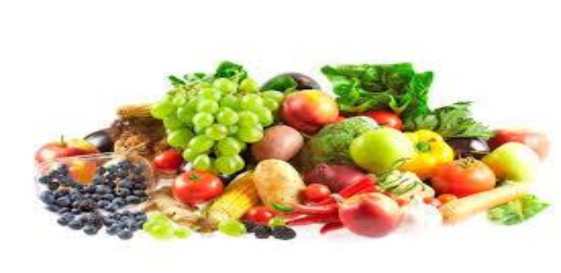

ಆಹಾರಕ್ರಮ

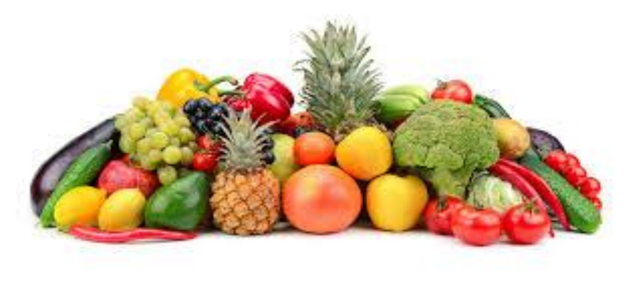

•ಮಧುಮೇಹ ಆಹಾರದಲ್ಲಿ ಹೆಚ್ಚಿನ ಸಂಖ್ಯೆಯ ಹಸಿ ತರಕಾರಿಗಳು ಒಳಗೊಂಡಿರಬೇಕು

•ಸಣ್ಣ ಪ್ರಮಾಣದಲ್ಲಿ ಆಹಾರವನ್ನು ಆಗಾಗ ಸೇವಿಸಿ

•ಸಿಹಿ ಮತ್ತು ಬೇಕರಿ ಪದಾರ್ಥಗಳನ್ನು ಸೇವಿಸಬೇಡಿ

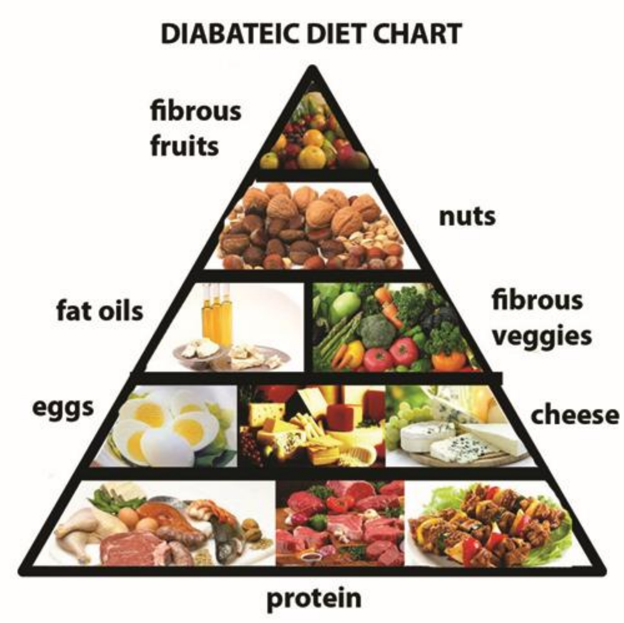

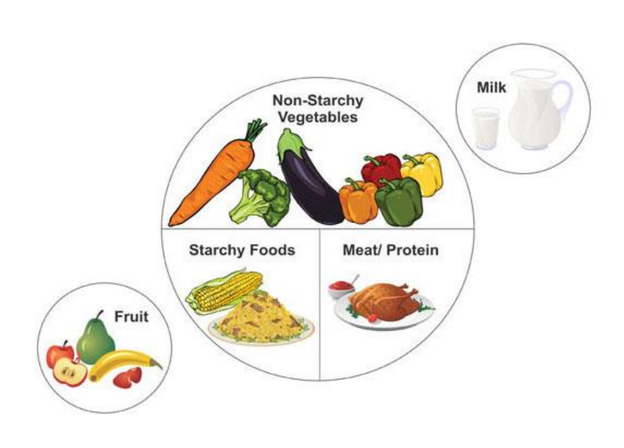

ಧವಸ / ಧಾನ್ಯಗಳು

ಸಕ್ಕರೆ ಕಾಯಿಲೆ ಇರುವವರು ಅಕ್ಕಿ ತೆಗೆದುಕೊಳ್ಳಬಾರದು.ಬರಿ ರಾಗಿ, ಗೋಧಿಯನ್ನು ಮಾತ್ರಉಪಯೋಗಿಸಬೇಕು

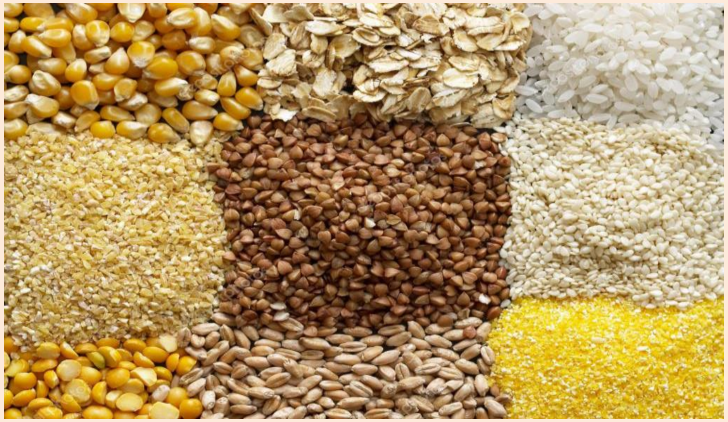

ಕಾಳುಗಳು/ಬೇಳೆಗಳು

ಕಾಳುಗಳು, ಬೇಳೆಗಳನ್ನು ನಿತ್ಯಆಹಾರದಲ್ಲಿ ಸೇರಿಸಿಕೊಳ್ಳುವುದರಿಂದ ಉತ್ತಮಪ್ರೋಟೀನ್ ಸಿಗುತ್ತದೆ

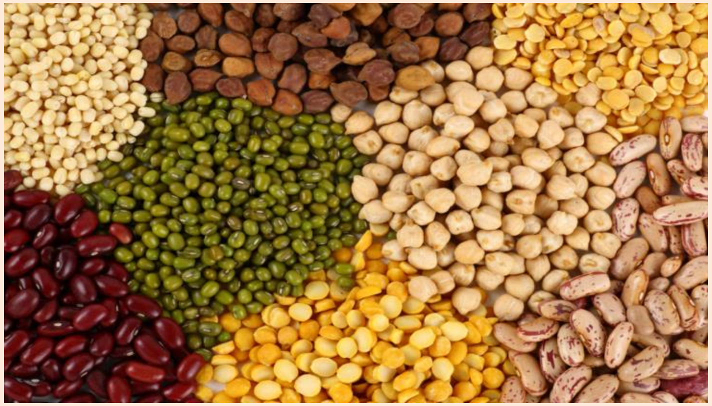

ತರಕಾರಿಗಳು

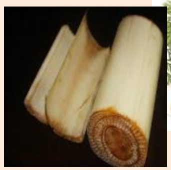
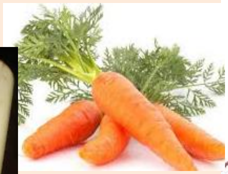
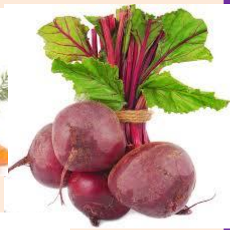

ಈ ತರಕಾರಿಗಳನ್ನು ಮಿತವಾಗಿ ಬಳಸಿ

ಕ್ಯಾರೆಟ್ (ಹಸಿ), ಬೀಟ್ರೋಟ್ (ಹಸಿ), ಬಾಳೆಕಾಯಿ, ಹಲಸಂದೆ ಕಾಯಿ, ಹಸಿ ಅವರೆಕಾಯಿ, ಬಾಳೆದಿಂಡು, ಚಪ್ಪರದ ಅವರೆಕಾಯಿ

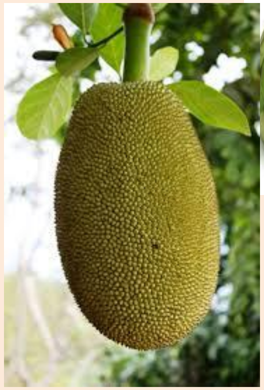
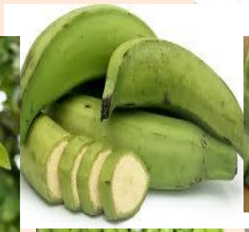
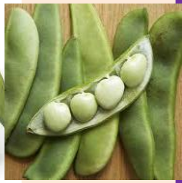
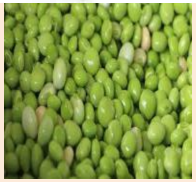
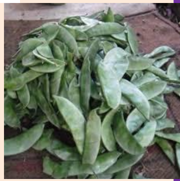

ಈ ತರಕಾರಿಗಳನ್ನು ಮಿತವಾಗಿ ಬಳಸುವುದು

ಸಿಹಿಗೆಣಸು, ಸುವರ್ಣಗೆಡ್ಡೆ, ಆಲೂಗೆಡ್ಡೆ, ಕ್ಯಾರೆಟ್, ಬೀಟ್ರೋಟ್

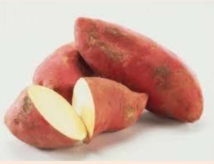
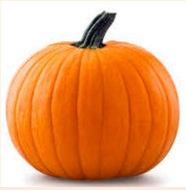
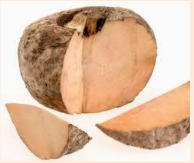
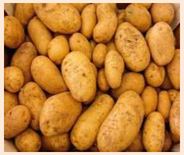

ಎಲ್ಲಾತರಹದ ತರಕಾರಿಗಳು ಹಾಗೂ ಸೊಪ್ಪುಗಳನ್ನು ಧಾರಾಳವಾಗಿ ಸೇವಿಸುವುದು

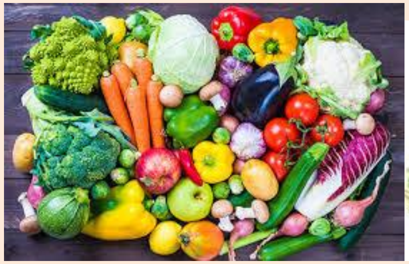
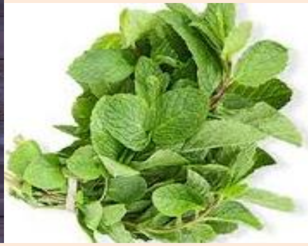
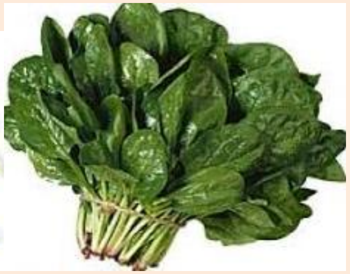
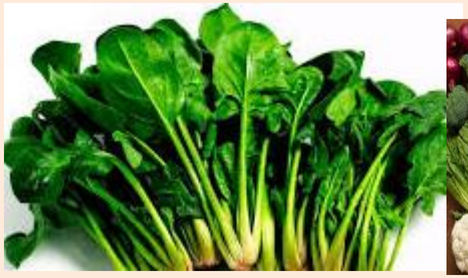
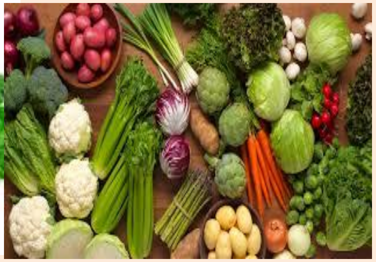

ಹಣ್ಣು

- ಹಣ್ಣುಗಳಲ್ಲಿ ಕೂಡ ಸ್ವಲ್ಪ ಪ್ರಮಾಣದ ಸಕ್ಕರೆ ಇರುತ್ತದೆ

- ಸಕ್ಕರೆ ಕಾಯಿಲೆ ನಿಯಂತ್ರಣದಲ್ಲಿದ್ದರೆ ಮಾತ್ರ ಕೆಳಗೆ ಕೊಟ್ಟಿರುವ ಹಣ್ಣುಗಳಲ್ಲಿ ದಿನಕ್ಕೊಂದು ಹಣ್ಣು ಮಾತ್ರ ತೆಗೆದುಕೊಳ್ಳಬಹುದು

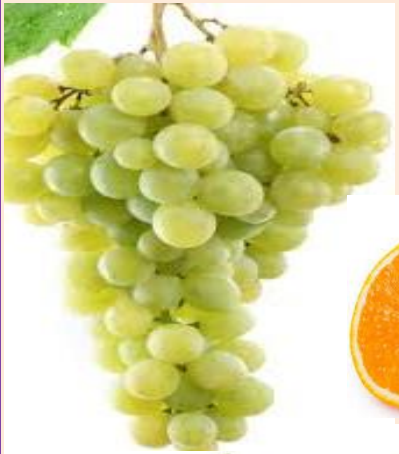
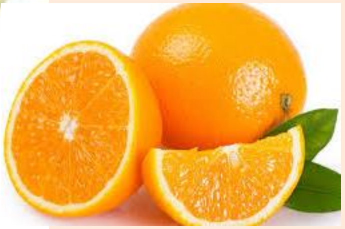
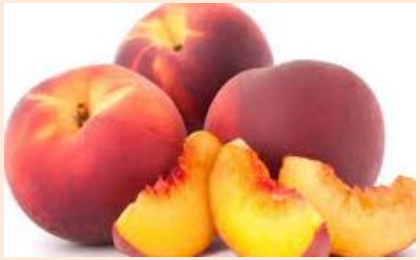
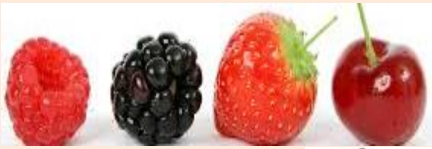
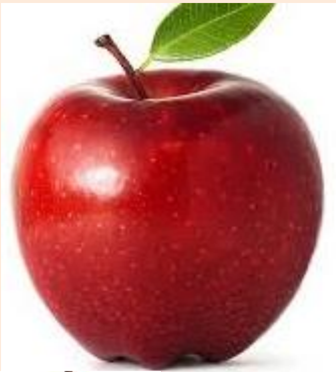
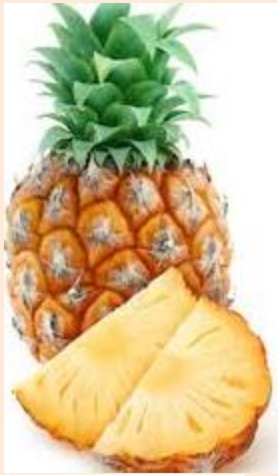
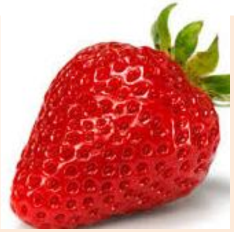
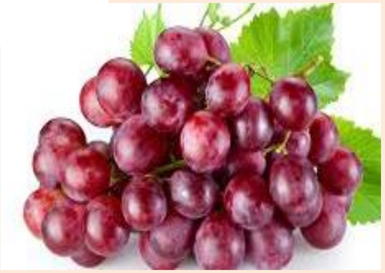
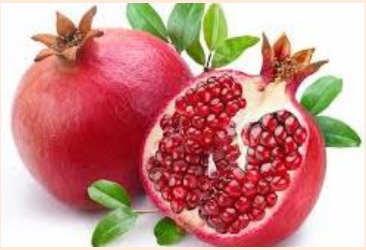
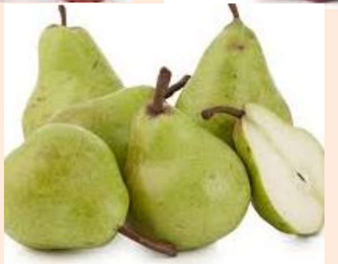
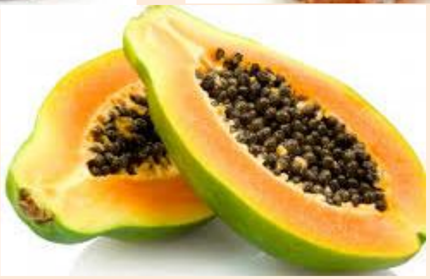

ಹಾಲು ಮತ್ತು ಹಾಲಿನ ಉತ್ಪನ್ನಗಳು

ಕೆನೆರಹಿತ ಹಾಲು, ಕೆನೆತೆಗೆದ ಮೊಸರು ಮತ್ತು ಮಜ್ಜಿಗೆಯನ್ನು ಉಪಯೋಗಿಸಬಹುದು

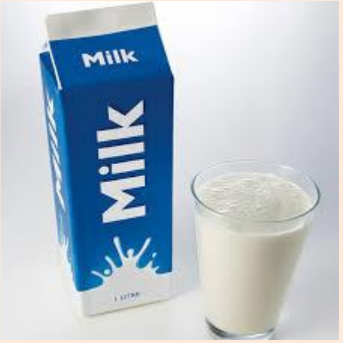

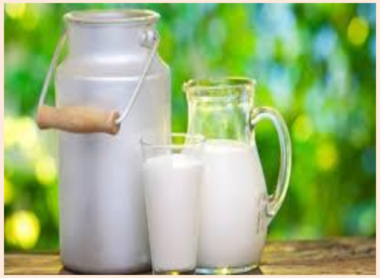

ಹಾಲಿನ ಇತರೆ ಉತ್ಪನ್ನಗಳಾದ ಖೋವಾ, ಪನ್ನೀರ್‌ಚೀಸ್, ಕ್ರೀಮ್, ಗಿಣ್ಣು, ತುಪ್ಪ, ಬೆಣ್ಣೆ ಇವುಗಳನ್ನು ಅಪರೂಪಕ್ಕೊಮ್ಮೆ ಬಹಳ ಮಿತವಾಗಿ ಬಳಸಬಹುದು

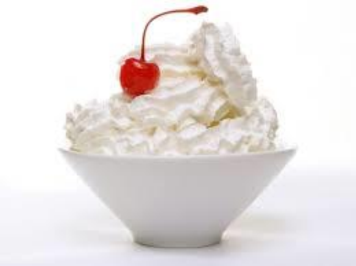

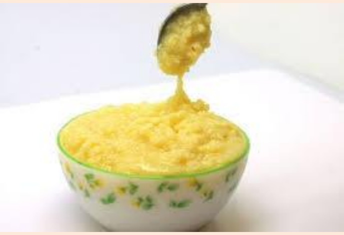

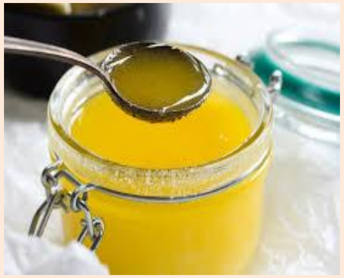

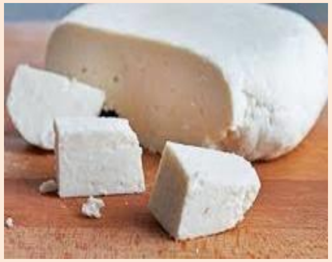

ಎಣ್ಣೆ

- ಸೂರ್ಯಕಾಂತಿ, ರಿಫೈನ್ಡ್‌ಆಯಿಲ್, ಕಾರ್ನ್‌ಆಯಿಲ್ ಇವುಗಳನ್ನು ಪ್ರತಿದಿನ 20 ಗ್ರಾಂಕ್ಕಿಂತ ಹೆಚ್ಚಾಗಿ ಉಪಯೋಗಿಸಬಾರದು

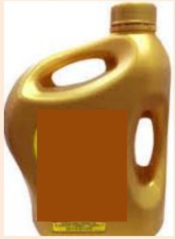
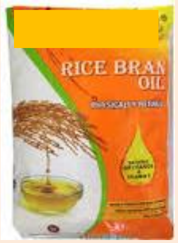

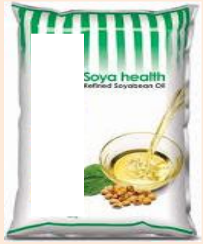

- ಡಾಲ್ಡಾ, ವನಸ್ಪತಿ ಇವುಗಳನ್ನು ಉಪಯೋಗಿಸಬಾರದು

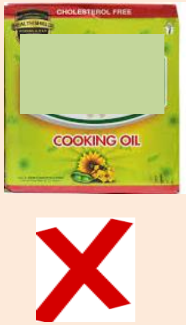

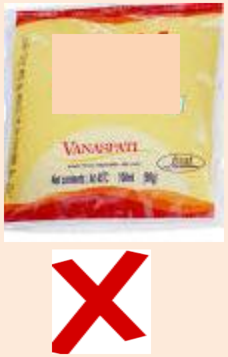

- ಬೆಣ್ಣೆ, ತುಪ್ಪ, ಅತಿಕಡಿಮೆ ಪ್ರಮಾಣದಲ್ಲಿ ಬಳಸಬೇಕು.

- ಎಣ್ಣೆಯಲ್ಲಿ ಕರಿದ ಪದಾರ್ಥಗಳನ್ನು ಕಡಿಮೆ ಬಳಸಬೇಕು.

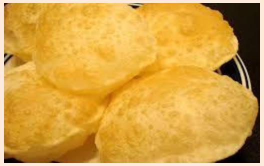
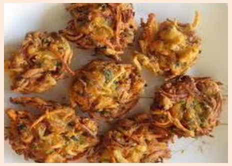
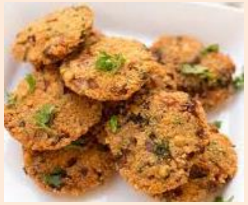
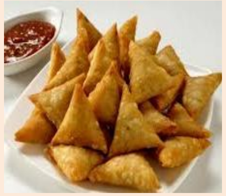
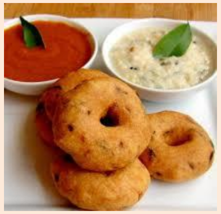

ಎಣ್ಣೆ ಬೀಜ

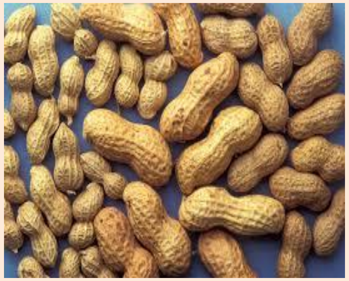
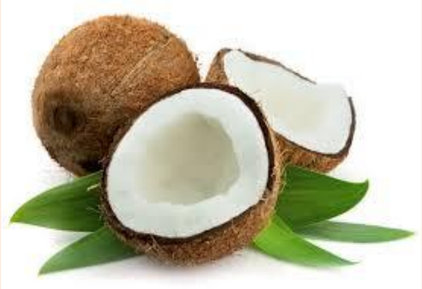

ಬಾದಾಮಿ, ಗೋಡಂಬಿ, ತೆಂಗಿನಕಾಯಿ, ಕಡಲೆಕಾಯಿ ಬೀಜ ಇವುಗಳನ್ನು ಮಿತವಾಗಿ ಪ್ರಮಾಣದಲ್ಲಿ ಬಳಸಬೇಕು

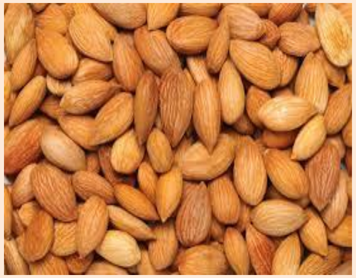
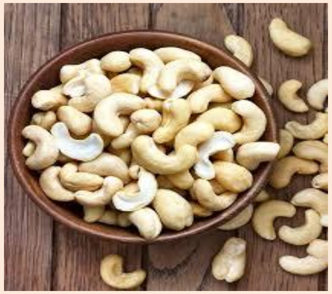

ಮಾಂಸಹಾರ

ಮಾಂಸದಿಂದ ತಯಾರಿಸಿದ ಆಹಾರ ಪದಾರ್ಥಗಳನ್ನು ಕಡಿಮೆ ಮಟ್ಟದಲ್ಲಿ ಉಪಯೋಗಿಸಬೇಕು

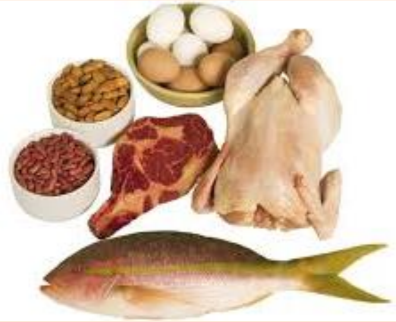
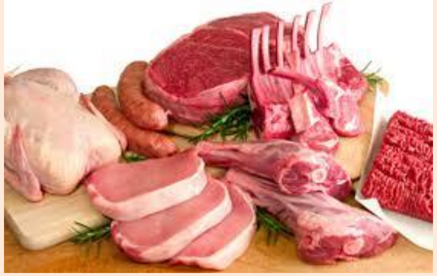

ಸಕ್ಕರೆ ಕಾಯಿಲೆ ಇರುವವರು ತೊರೆಯಬೇಕಾದ ಆಹಾರಗಳು

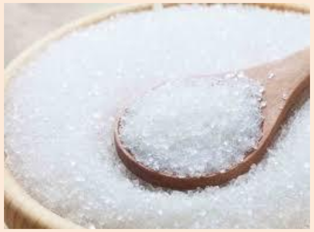

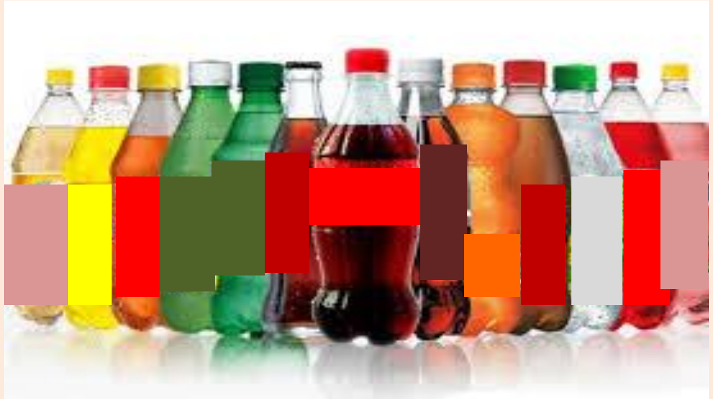

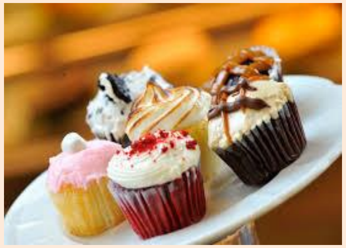

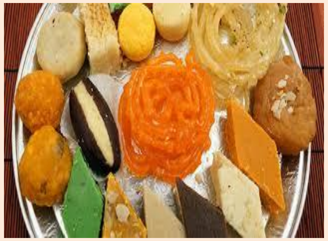

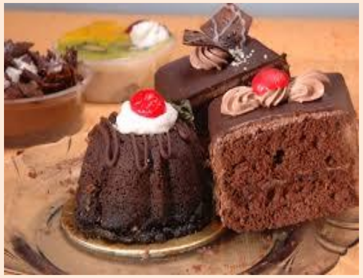

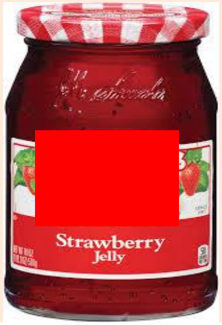

ಸಕ್ಕರೆ, ಜಿಲ್ಲಿ, ಜ್ಯಾಂ, ಗ್ಲೂಕೋಸು, ಸಿಹಿ ಪದಾರ್ಥ, ಐಸ್‌ಕ್ರೀಂ, ಪೇಯಗಳು ಕೊಕೊ ಪಾನೀಯಗಳು, ಚಾಕಲೇಟ್ **ಪದಾರ್ಥಗಳನ್ನು ಸೇವಿಸಬೇಡಿ**

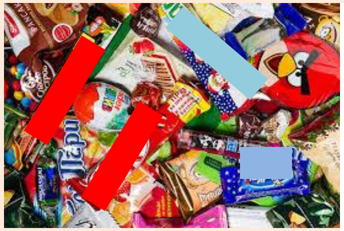

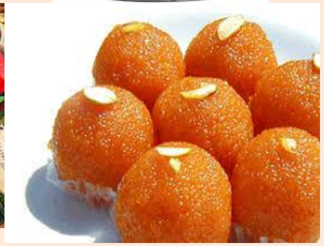

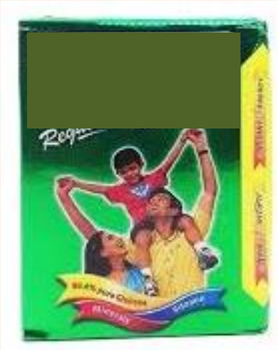

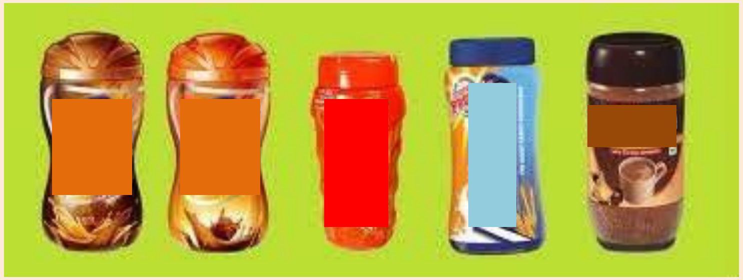

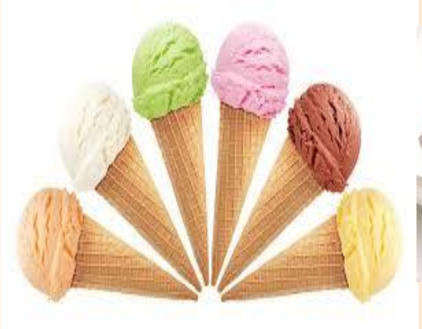

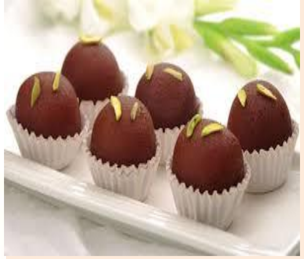

ಔಷದೋಪಚಾರದ ನಿಷೆ

ಮಧುಮೇಹ (ಡಯಾಬಿಟಿಸ್) ಔಷಧವು ರಕ್ತದಲ್ಲಿನ ಸಕ್ಕರೆಯನ್ನು ನಿಯಂತ್ರಿಸುತ್ತದೆ. ಆದ್ದರಿಂದ ವೈದ್ಯರು ಸೂಚಿಸಿದಂತೆ ಯಾವಾಗಲೂ ನಿರ್ಧಿಷ್ಟ ಸಮಯದಲ್ಲಿ ಔಷಧಿಗಳನ್ನು ತೆಗೆದುಕೊಳ್ಳಿ ಹಾಗೆ ವೈದ್ಯರನ್ನು ತಪ್ಪದೇ ಭೇಟಿ ಮಾಡಿ

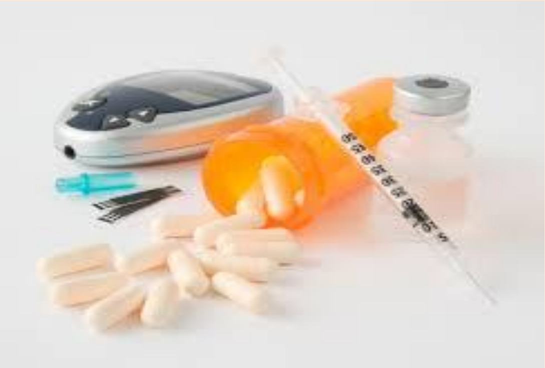

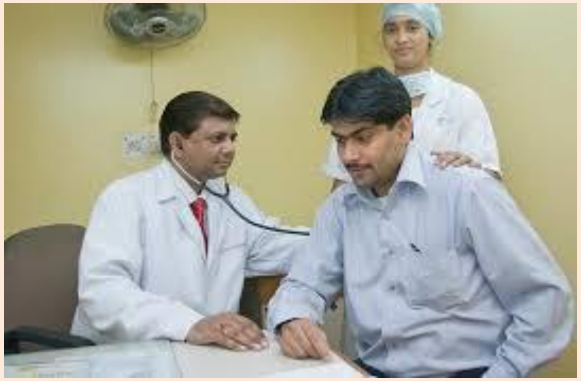

ದೈಹಿಕ ವ್ಯಾಯಾಮ

ದೈಹಿಕ ವ್ಯಾಯಾಮ ರಕ್ತದಲ್ಲಿನ ಸಕ್ಕರೆ ಅಂಶವನ್ನು ನಿಯಂತ್ರಣದಲ್ಲಿಟ್ಟುಕೊಳ್ಳುವಲ್ಲಿ ಮಹತ್ವದ ಪಾತ್ರ ವಹಿಸುತ್ತದೆ. ಆದ್ದರಿಂದ ಕನಿಷ್ಟ 30 ನಿಮಿಷಗಳ ಕಾಲ ದೈನಂದಿನ ವ್ಯಾಯಾಮ ಮಾಡಿ

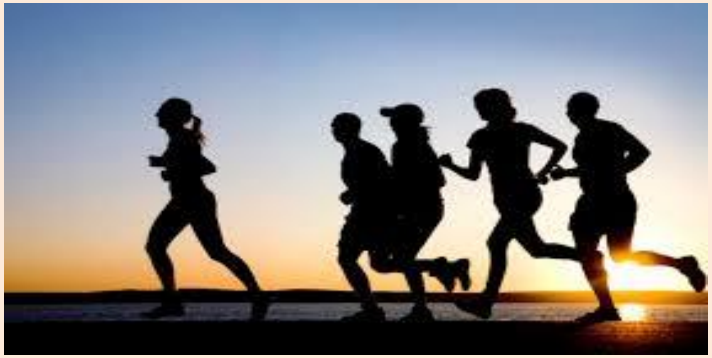

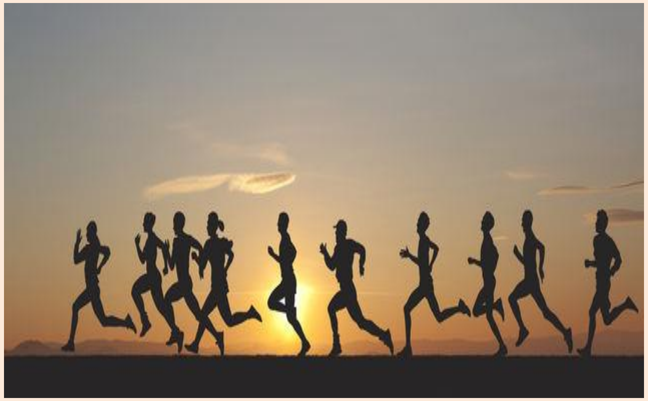

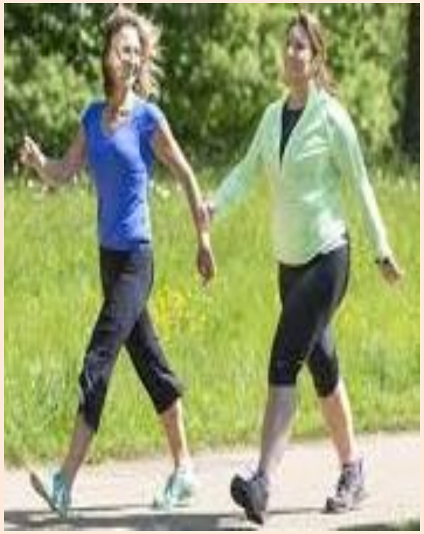

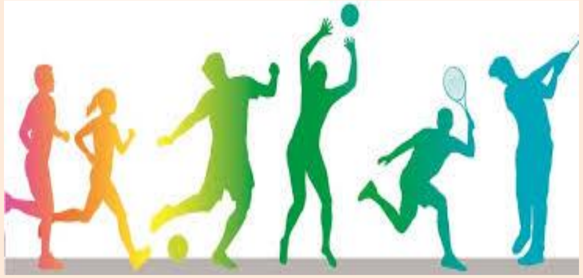

ಕಣ್ಣಿನ ಸ್ಥಿತಿಯ ತಪಾಸಣೆ

- ಪ್ರತಿ 6 ತಿಂಗಳಿಗೊಮ್ಮೆ ಮಧುಮೇಹದ ರೋಗಿಗಳು ಕಣ್ಣಿನ ತಜ್ಞರನ್ನು ಕಂಡು ತಮ್ಮ ಕಣ್ಣುಗಳನ್ನು ಸುಸ್ಥಿತಿಯಲ್ಲಟ್ಟುಕೊಳ್ಳುವುದು.

ಈ ಕೆಳಗಿನ ಸಮಸ್ಯೆಗಳು ನಿಮಗೆ ಇದ್ದಲ್ಲಿ,

- ಮಸುಕಾದ ದೃಷ್ಟಿದೋಷ
- ಕಣ್ಣಿನ ಉರಿ ಹಾಗೂ ನವೆ
- ದುಸ್ಥಿತಿಯ ದೃಷ್ಟಿ

ಕಣ್ಣುಗಳನ್ನು ಕಾಪಾಡಿ

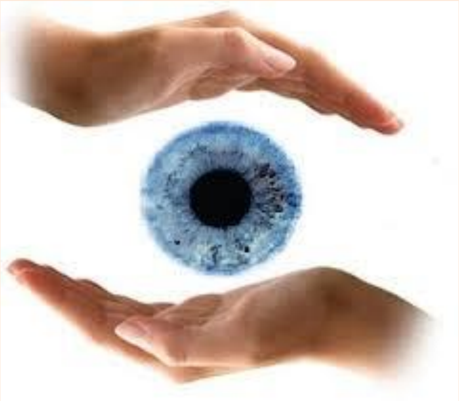

ದಯಮಾಡಿ ನಿಮ್ಮ ವೈದ್ಯರನ್ನು ಕೂಡಲೇ ಭೇಟಿಮಾಡಿ

ಪಾದಗಳ ಪರಿಶೀಲನ

- ಪ್ರತಿನಿತ್ಯ ಪಾದಗಳನ್ನು ಗಮನಿಸಿಕೊಳ್ಳಿ
- ಕೆಂಪುಚುಕ್ಕೆಗಳು, ಊತ ಹಾಗೂ ಬಿರುಕುಗಳು ಕಂಡುಬಂದಲ್ಲಿ.
- ಪ್ರತಿನಿತ್ಯ ನಿಮ್ಮ ಪಾದಗಳನ್ನು ಬೆಚ್ಚನೆಯ ನೀರಿನಲ್ಲಿ ತೊಳೆದುಕೊಳಿ
- ನಿಮ್ಮ ಪಾದಗಳು ಎಂದಿಗೂ ಮೃದುವಾಗಿರಲಿ.
- ಉಗುರುಗಳನ್ನು ವಾರಕ್ಕೊಮ್ಮೆ ಕತ್ತರಿಸಿ.
- ಬರಿಗಾಲಿನಲ್ಲಿ ನಡೆಯಬೇಡಿ

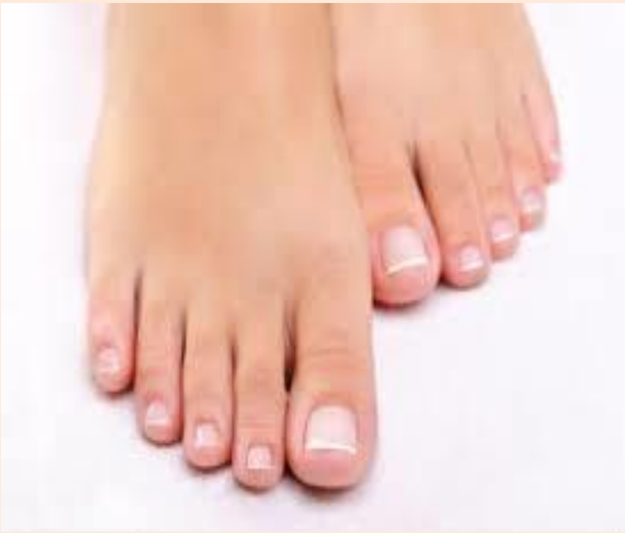

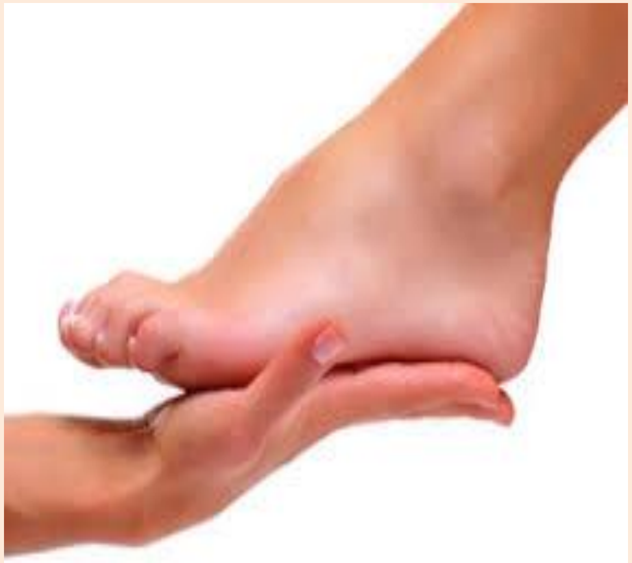

ಮಧುಮೇಹ ಸ್ಥಿತಿಯನ್ನು ಗಮನಿಸಿಕೊಳಿ :

HbA1C ಎಂದರೇನು ?

- ಪ್ರತಿ 3 ತಿಂಗಳಿಗೊಮ್ಮೆ ರಕ್ತದಲ್ಲಿನ ಗ್ಲುಕೋಸ್ (ಸಕ್ಕರೆ) ಮಟ್ಟವನ್ನು ಮತ್ತು ಅದರ ಪ್ರಮಾಣವನ್ನು ಅಳತೆ ಮಾಡುತ್ತದೆ

- HbA1C ಯ ಪ್ರಮಾಣದಿಂದ ಬಹಳ ಕಾಲದವರೆಗೆ ಮಧುಮೇಹವನ್ನು ನಿಯಂತ್ರಣದಲ್ಲಿಟ್ಟುಕೊಳ್ಳಬಹುದು

ರಕ್ತದಲ್ಲಿನ ಸಕ್ಕರೆ : 80-120 mg/dl

HbA1C< 7

ಮಧುಮೇಹದಿಂದಾಗುವ ತೀವ್ರತರವಾದ ಸಮಸ್ಯೆಗಳು

ಸಕ್ಕರೆ ಕಾಯಿಲೆಯನ್ನು ನಿಯಂತ್ರಣದಲ್ಲಿಡದೆ ಹೋದರೆ ತಲೆದೋರುವ ದೀರ್ಘಾವಧಿಯ ನಸಮಸ್ಯಗಳು

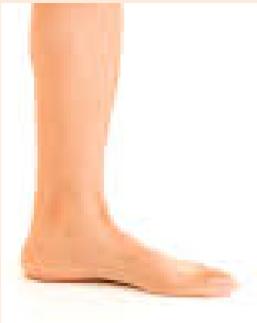

ಕಾಲು ಮತ್ತು ಪಾದಗಳ ತೊಂದರೆ

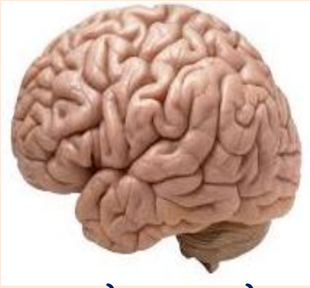

ಪಾರ್ಶ್ವವಾಯು

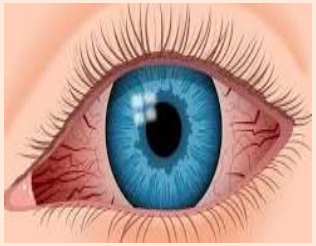

ಕಣ್ಣಿನ ಸಮಸ್ಯೆ

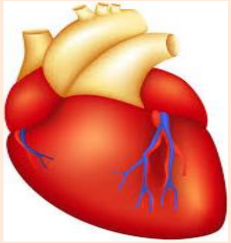

ಹೃದಯಘಾತ

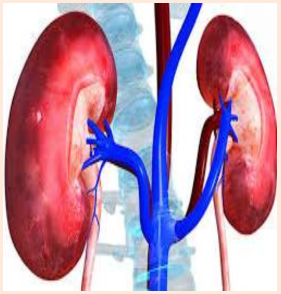

ಕಿಡ್ನೀ ತೊಂದರೆ

For

Diabetic Care

Please Contact

ಮಧುಮೇಹ ಆರೈಕೆಗಾಗಿ ದಯವಿಟ್ಟು ಸಂಪರ್ಕಿಸಿ

JSS HOSPITAL

Mahathma Gandhi Road, Mysuru-570 004,
Karnataka, India.
Phone : 0821-2335555
Fax : 0821-2335556
Email : contact@jsshospital.in

Special Clinic Appointments

(Working Hours : 8AM to 6.30PM) * Sunday Holiday

0821-2335261 / 5267 or +91-70220-32866
