# Supplementary material for: Intervention through Short Messaging System (SMS) and phone call alerts reduced HbA1C levels in ~47% type-2 diabetics–results of a pilot study
Source: PLoS One. 2020 Nov 17;15(11):e0241830. doi: 10.1371/journal.pone.0241830 (PMC7671489; doi:10.1371/journal.pone.0241830)
Supplement: S7 File — Informed consent forms were made in English and local language Kannada to obtain participants consent for enrolling into the study. (PDF) [file pone.0241830.s007.pdf]

This Informed Consent Form is for patients who attend JSS Hospital, and whom we are inviting to participate in research titled as "Effectiveness of Self Management in Type2 Diabetic Patients through Information Communication Technology (ICT) Interventions"

Principal Investigator:

Kanakavalli K. Kundury

Lecturer

Department of Health System Management Studies

JSS University

Mysuru - 570015

Information Sheet

Introduction

I'm Kanakavalli K. Kundury, working for JSS University. We are doing a research on **Effectiveness of Self Management in Type2 Diabetic Patients through ICT Interventions**. I'm going to give you information and invite you to be a part of this research. There may be some words that you do not understand. Please ask me in this regard, and I'll take time to explain. Before you decide on participating in this research, if you wish, you can discuss with your friends or family about the research

Purpose of the research

Diabetes is one of the most common diseases and according to 2015, Global Health Reports; India is the Diabetic capital of the world. The ill effects of diabetes include extreme thirst, weight loss, and excessive urination, lack of concentration, foot problems, heart diseases, and stroke, cataract and vision defects. The burden of this disease not only effects the population and productivity but also creates additional healthcare expenditure on nations. Diabetes can be managed to a great extent with patient education, and systematically monitoring changes in patients with diabetes, their health status and support self care. Various Diabetic Educator Programs were initiated across nations around the globe; to facilitate self monitoring of diabetes. Increasing acceptance of Information and Communication Technology is making the healthcare information available at finger tips. In the present research, we are encouraging the patients to continuously self monitor the diabetes by Information and Communication Technological tools that we provide.

Type of Research Intervention

After giving due consent to participate in the study with a valid mobile number, participants will be receiving diabetic educational messages and calls, encouraging them to consciously monitor their glycaemic parameters. Also web access to diabetic educational information will be facilitated and detailed instructions will be provided to access the same. Periodic glycaemic readings of the participants will be collected to assess the effectiveness of self management of diabetes using ICT tools.

Participant selection

We are inviting the patients with Diabetes who attend JSS Hospital to participate in the research on **Effectiveness of Self Management in Type2 Diabetic Patients through ICT Interventions**

Voluntary Participation

Your participation in this research is entirely voluntary. It is your choice whether to participate or not. Whether you choose to participate or not, all the services you receive at this clinic will continue and nothing will change. You may change your mind later and stop participating even if you have agreed earlier.

Duration

The research takes place over 12 months in total. Periodic glycaemic parameters of the participants will be collected for the study.

Confidentiality

The information that we collect from this research project will be kept confidential. The information collected during the research will be accessed only by researchers of the project.

Who to Contact

If you have any questions you may ask them now or later, even after the study has started. If you wish to ask questions later, you may contact: Kanakavalli K. Kundury, Lecturer, Department of Health System Management Studies, JSS University, Mysore. Mobile : 9980803301. e-mail: kirankundury@gmail.com

This proposal has been reviewed and approved by Ethics Review Committee, JSS University.

Study Title: **Effectiveness of Self Management in Type-2 Diabetic Patients through Information Communication Technology (ICT) Interventions**

Subject’s Initials: _______________ Subject’s Name:_______________

Date of Birth / Age: _________________

(i)	I confirm that I have read and understood the information sheet of the above study and have had the opportunity to ask questions.	[ ]
(ii)	I understand that my participation in the study is voluntary and that I am free to withdraw at any time, without giving any reason, without my medical care or legal rights being affected.	[ ]
(iii)	I understand that the Ethics Committee and the regulatory authorities will not need my permission to look at my health records both in respect of the current study and any further research that may be conducted in relation to it, even if I withdraw from the study. However, I understand that my identity will not be revealed in any information released to third parties or published.	[ ]
(iv)	I agree not to restrict the use of any data or results that arise from this study provided such a use is only for scientific purpose(s)	[ ]
(v)	I agree to take part in the above study.	[ ]

Signature (or Thumb impression) of the Subject with date: ______________________

Name and Signature of the Witness with date ______________________

Name and Signature of the Principal Investigator with date: _________________________________
