# Supplementary figures and images for: Intervention through Short Messaging System (SMS) and phone call alerts reduced HbA1C levels in ~47% type-2 diabetics–results of a pilot study
Source: PLoS One. 2020 Nov 17;15(11):e0241830. doi: 10.1371/journal.pone.0241830 (PMC7671489; doi:10.1371/journal.pone.0241830)

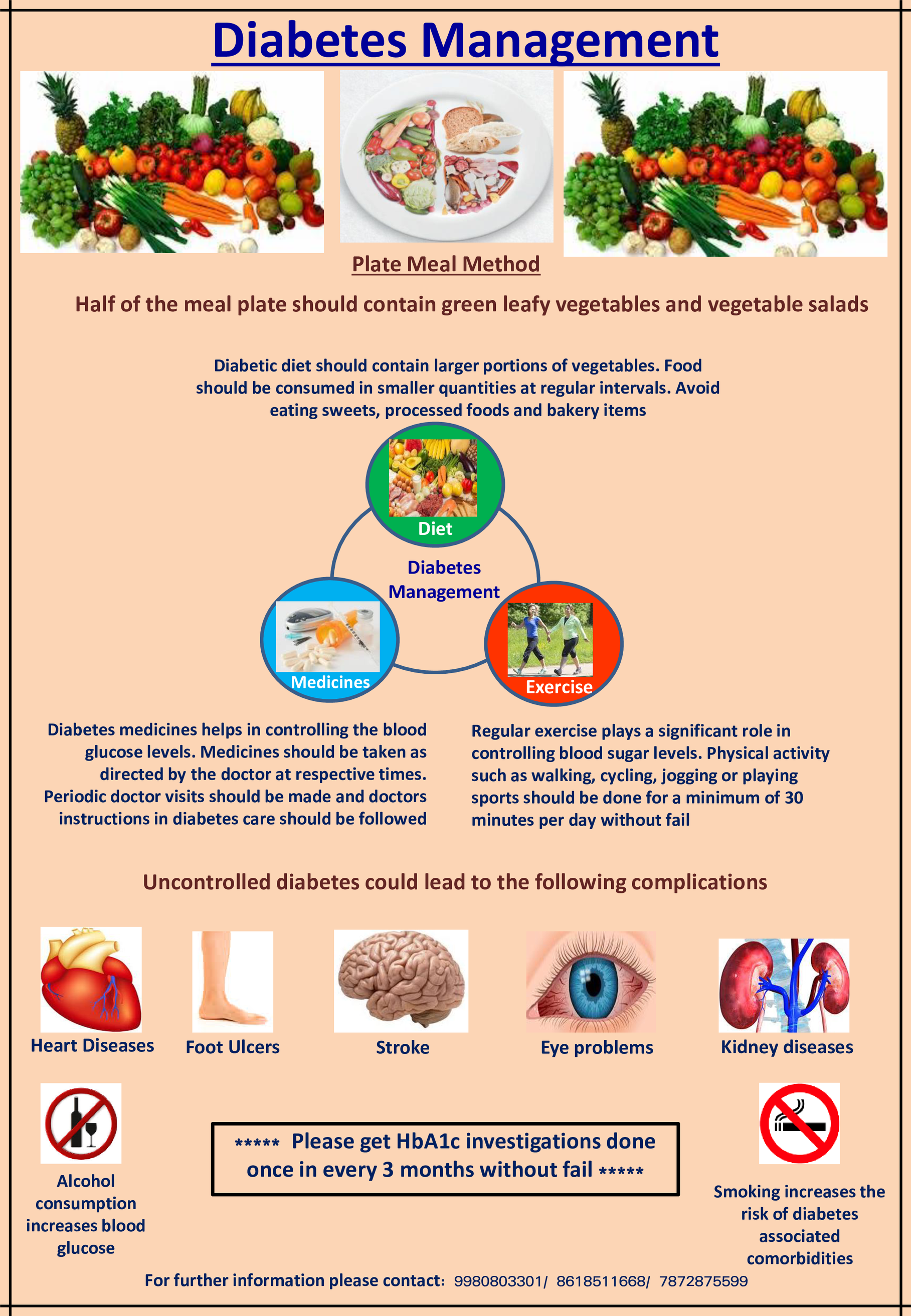

Supplement: S8 File — Participants were given handouts containing information pertaining to diabetes education and pictures that reinforces their learning during orientation. The handout also facilitates them to enter blood investigations readings and, to keep track of their glucose levels. Handouts were made available in local language Kannada as well as in English. (TIF) [file pone.0241830.s008.tif]

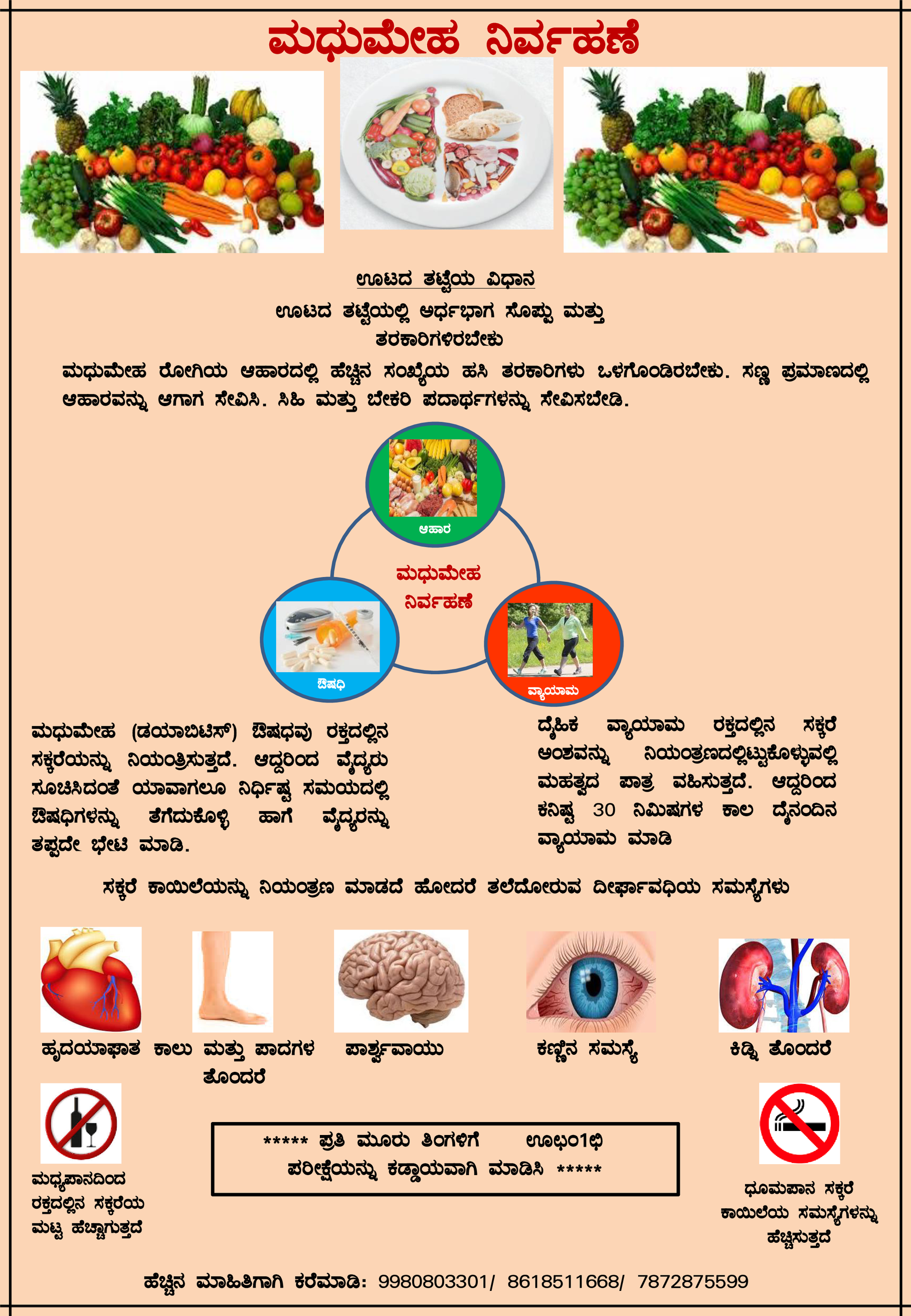

Supplement: S9 File — Participants were given handouts containing information pertaining to diabetes education and pictures that reinforces their learning during orientation. The handout also facilitates them to enter blood investigations readings and, to keep track of their glucose levels. Handouts were made available in local language Kannada as well as in English. (TIF) [file pone.0241830.s009.tif]

## Study Design

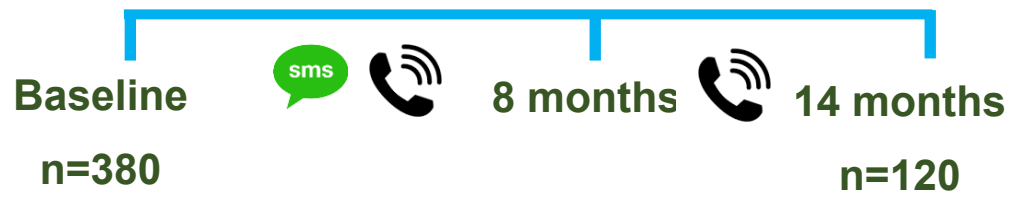

Supplement: S13 File — The scheme shows the overall study design. The study initially recruited 380 participants with diabetes. These participants had received diabetes education through weekly SMS and monthly phone calls for a period of 8months. Upon considering the participants feedback preferring phone calls over SMS messages, weekly phone call-based diabetes education was provided for an additional 6-months period. A total of 120 participants completed the 14 months study. (PDF) [file pone.0241830.s013.pdf]

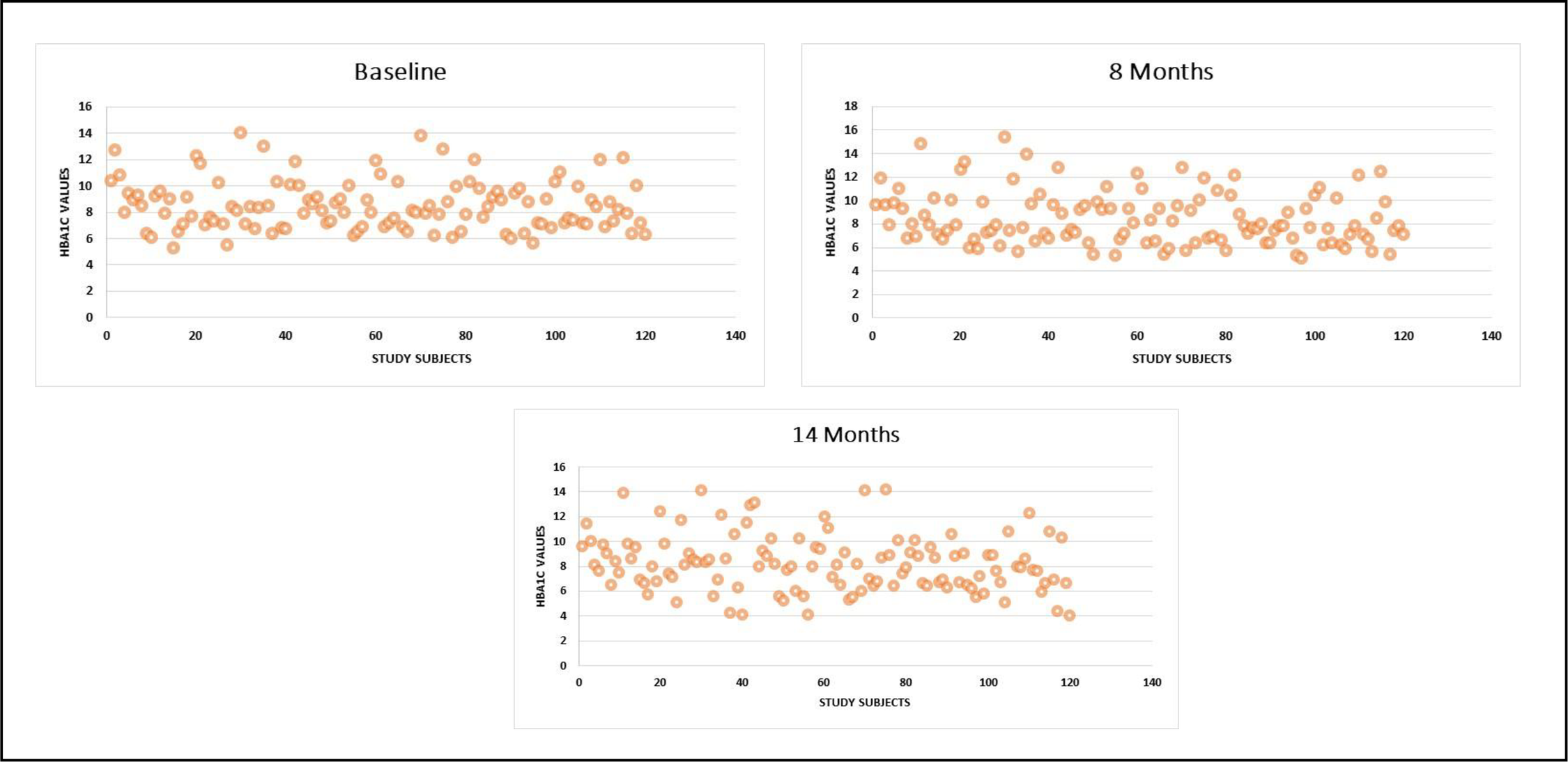

Supplement: S18 File — The figure demonstrates the HbA1c values of 120 study participants across baseline, 8 months and 14 months of study periods. (TIF) [file pone.0241830.s018.tif]

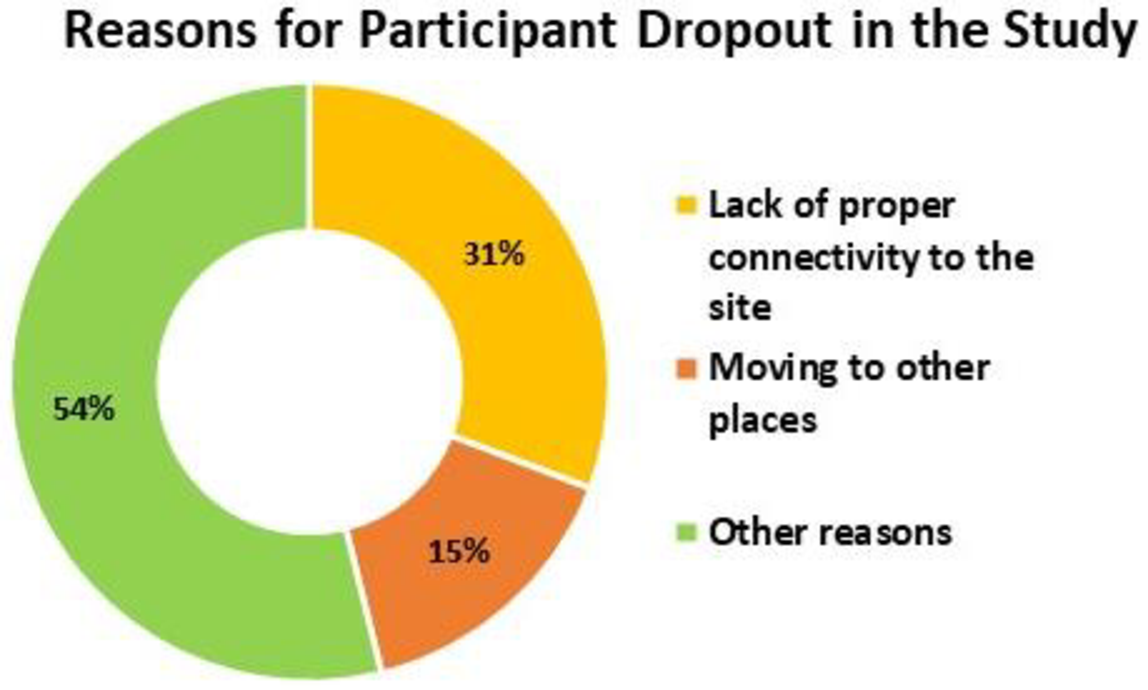

Supplement: S19 File — The doughnut graph depicts the various reasons for 2/3rds of the participants non-adherence to the current study. (TIF) [file pone.0241830.s019.tif]
