# Supplementary material for: Intervention through Short Messaging System (SMS) and phone call alerts reduced HbA1C levels in ~47% type-2 diabetics–results of a pilot study
Source: PLoS One. 2020 Nov 17;15(11):e0241830. doi: 10.1371/journal.pone.0241830 (PMC7671489; doi:10.1371/journal.pone.0241830)
Supplement: S10 File — Periodic messages (SMS) that were sent to the participants during the study period of first 8 months. (PDF) [file pone.0241830.s010.pdf]

Annexure-5
Diabetes Educational Short Messages Sent to Study Participants

ಮಧುಮೇಹ ನಿರ್ವಹಣೆ ಕುರಿತು ಕಾರ್ಯಕ್ರಮಾ ಜೆ. ಎಸ್. ಎಸ್ ಆಸ್ಪತ್ರೆ (ಮಧುಮೇಹ ಪರೀಕ್ಷೆ ಮಾಡುವ ಕೊಠಡಿಯ ಸಮೀಪ)
ಸಮಯ: ಬೆಳಗ್ಗೆ 10.30
ದಿನಾಂಕ: 9.12.17 ಶನಿವಾರ
ಉಚಿತ ಮಧುಮೇಹ ಪರೀಕ್ಷೆ ಮಾಡಲಾಗುವುದು

Diabetes Management Program at JSS Hsopital
Time: 10.30 am
Date: Saturday 9.12.2017
Free blood investigations will be conducted
****

****ಮಧುಮೇಹ ನಿರ್ವಹಣೆ****

💐ಹೊಸ ವರ್ಷದ ಹಾರ್ಧಿಕ ಆರೋಗ್ಯದಾಯಕ ಶುಭಾಷಯಗಳು 💐

Diabetes Management
Wish you a very happy new year
****

"ನಿಮ್ಮ ಮಧುಮೇಹ ನಿಮ್ಮ ನಿರ್ವಹಣೆಯ ಕೈಯಲ್ಲಿ"
ನಿಯಮಿತವಾಗಿ ಕೆಳಗಿನ ನಿಯಮಗಳನ್ನು ಕಡ್ಡಾಯವಾಗಿ ಪಾಲಿಸಿ
🍱ನಿಯಮಿತವಾಗಿ ಆಹಾರವನ್ನು ಸ್ವಲ್ಪ ಸ್ವಲ್ಪ ಸೇವಿಸಿ
🏥ವೈದ್ಯರನ್ನು ಸಂಪರ್ಕಿಸಿ
💊 ಔಷಧಗಳನ್ನು ತೆಗಿದುಕೊಳ್ಳಿ
🏃 ಧೈಹಿಕ ವ್ಯಾಯಾಮ ಮಾಡಿ
****ಮಧುಮೇಹ ನಿರ್ವಹಣೆ****

Your diabetes in your control
Kindly follow the below
Take limited quantities of food at regular intervals
Visit your doctor periodically
Take your medications as prescribed by your doctor
Regularly do your physical activity
******* Diabetes Management*******
****

ಮಧುಮೇಹ (ಡಯಾಬಿಟಿಸ್) ಔಷಧವು ರಕ್ತದಲ್ಲಿನ ಸಕ್ಕರೆಯನ್ನು ನಿಯಂತ್ರಿಸುತ್ತದೆ. ಆದ್ದರಿಂದ ವೈದ್ಯರು ಸೂಚಿಸಿದಂತೆ ಯಾವಾಗಲೂ ನಿರ್ಧಿಷ್ಟ ಸಮಯದಲ್ಲಿ ಔಷಧಿಗಳನ್ನು ತೆಗೆದುಕೊಳ್ಳಿ ಹಾಗೆ ವೈದ್ಯರನ್ನು ತಪ್ಪದೇ ಭೇಟಿ ಮಾಡಿ.

Diabetic medications will control the blood glucose values. As directed by your doctor adhere to your medications and visit doctor periodically

****

ಮಧುಮೇಹ ನಿರ್ವಹಣೆ
🥗ಮಧುಮೇಹ ಆಹಾರದಲ್ಲಿ   ಹೆಚ್ಚಿನ ಸಂಖ್ಯೆಯ ಹಸಿ ತರಕಾರಿಗಳನ್ನು ಒಳಗೊಂಡಿರಬೇಕು🥗
🥬🥒🥦🥕🥦🥒🥕🥬
ಸಣ್ಣ ಪ್ರಮಾಣದಲ್ಲಿ ಆಹಾರವನ್ನು ಆಗಾಗ ಸೇವಿಸಿ
🥬🥒🥦🥕🥦🥒🥕🥬
ಮಧುಮೇಹ ನಿರ್ವಹಣೆ

** Diabetes Management**
Diabetic diet should contain a greater number of green vegetables
Consume small quantities of food at regular intervals
** Diabetes Management**

****

ಮಧುಮೇಹ ನಿರ್ವಹಣೆ
💊💉💊💉💊💉💊💉
ಮಧುಮೇಹ ನಿಯಂತ್ರಣ ಔಷಧವು ರಕ್ತದಲ್ಲಿನ ಸಕ್ಕರೆಯ ಅಂಶವನ್ನು ನಿಯಂತ್ರಿಸುತ್ತದೆ
ಆದ್ದರಿಂದ ವೈದ್ಯರು ಸೂಚಿಸಿದಂತೆ ಯಾವಾಗಲೂ ನಿರ್ಧಿಷ್ಟ ಸಮಯದಲ್ಲಿ ಔಷಧಗಳನ್ನು ತಗದುಕೊಳ್ಳಿ
💊💉💊💉💊💉💊💉 ಹಾಗೆ ವೈದ್ಯರನ್ನು ತಪ್ಪದೇ ಭೇಟಿ ಮಾಡಿ 🏥
ಮಧುಮೇಹ ನಿರ್ವಹಣೆ

** Diabetes Management**
Diabetes medications control the quantity of glucose in blood. So, as directed by your doctor don’t miss your medications. I required, consult your doctor
** Diabetes Management**

****

ಮಧುಮೇಹ ನಿರ್ವಹಣೆ
🏃🏃🏃🏃🏃🏃🏃🏃🏃🏃
ದೈಹಿಕ ವ್ಯಾಯಾಮವು ರಕ್ತದಲ್ಲಿನ ಸಕ್ಕರೆ ಅಂಶವನ್ನು ನಿಯಂತ್ರಣದಲ್ಲಿಟ್ಟುಕೊಳ್ಳುವಲ್ಲಿ ಮಹತ್ವದ ಪಾತ್ರ ವಹಿಸುತ್ತದೆ. ಆದ್ದರಿಂದ ಕನಿಷ್ಠ 30 ನಿಮಿಷಗಳ ಕಾಲ ದೈನಂದಿನ ದೈಹಿಕ ವ್ಯಾಯಾಮ ಮಾಡಿ
🏃🏃🏃🏃🏃🏃🏃🏃🏃🏃
ಮಧುಮೇಹ ನಿರ್ವಹಣೆ

** Diabetes Management**

Regular physical activity plays a significant role in controlling blood glucose. Physical activity for a minimum of 30 minutes to be done everyday

** Diabetes Management**

****

ಮಧುಮೇಹ ನಿರ್ವಹಣೆ
🥂ಮಧ್ಯಪಾನದಿಂದ ರಕ್ತದಲ್ಲಿನ ಸೆಕ್ಕರೆಯ ಮಟ್ಟ ಹೆಚ್ಚಾಗುತ್ತದೆ
🚭ಧೂಮಪಾನ ಸೆಕ್ಕರೆ ಕಾಯಲೆಯ ಸಮಸ್ಯೆಗಳನ್ನು ಹೆಚಸುತ್ತೆದೆ
ಮಧುಮೇಹ ನಿರ್ವಹಣೆ

** Diabetes Management**

Consumption of alcohol elevates the blood glucose values. Smoking increases the risk for diabetes associated complications. Consumption of alcohol and smoking should be avoided

** Diabetes Management**
