# Supplementary material for: Intervention through Short Messaging System (SMS) and phone call alerts reduced HbA1C levels in ~47% type-2 diabetics–results of a pilot study
Source: PLoS One. 2020 Nov 17;15(11):e0241830. doi: 10.1371/journal.pone.0241830 (PMC7671489; doi:10.1371/journal.pone.0241830)
Supplement: S11 File — The log sheet was designed to enter the collected information such as patient name, mobile number and SMS delivery status every time an SMS was sent to the study participant. (PDF) [file pone.0241830.s011.pdf]

Annexure-6

SMS Log Sheet

SNo	Patient ID	Gender	Age	Mobile Number	SMS sent (Y/N)	Remarks
