# Supplementary material for: Intervention through Short Messaging System (SMS) and phone call alerts reduced HbA1C levels in ~47% type-2 diabetics–results of a pilot study
Source: PLoS One. 2020 Nov 17;15(11):e0241830. doi: 10.1371/journal.pone.0241830 (PMC7671489; doi:10.1371/journal.pone.0241830)
Supplement: S12 File — Participants’ knowledge on diabetes management practices was assessed using a simple structured questionnaire along with their feedback on study design. (PDF) [file pone.0241830.s012.pdf]

Diabetes Knowledge Assessment Questionnaire

a) Symptoms of diabetes include ____________________________________

1. Excessive thirst
2. Hunger
3. Frequent urination
4. Weight loss

b) Best practices to keep diabetes under control include ______________________

1. Regular exercise
2. Controlled diet
3. Medication adherence
4. Periodic doctor visit

c) Uncontrolled diabetes could lead to these diseases______________________

1. Heart disease
2. Eye problems
3. Stroke
4. Neurological diseases
5. Kidney diseases
6. foot ulcers

d) Symptoms of hypo glycaemia includes

1. Dizziness
2. Hunger
3. Sweating
4. Shakiness
5. Anxiety
6. Moodiness

e) Excellent glucose control is indicated by which of the below ranges mentioned

1. <80 MG/DL
2. 80-120 MG/DL
3. 120-180 MG/DL
4. >180 MG/DL

f) Blood test which gives accurate reading of glucose value and has to be done every 3-4 months is _________________________

1. Fasting
2. PPBS
3. HbA1C

Program Feedback

1. Is the program helping you in self management of diabetes? Y____ N____

2. Are weekly phone calls helping you better than SMS?   Y____N ____

3. Are you confident to manage diabetes on your own?   Y_____ N_____
