# Supplementary material for: Intervention through Short Messaging System (SMS) and phone call alerts reduced HbA1C levels in ~47% type-2 diabetics–results of a pilot study
Source: PLoS One. 2020 Nov 17;15(11):e0241830. doi: 10.1371/journal.pone.0241830 (PMC7671489; doi:10.1371/journal.pone.0241830)
Supplement: S16 File — The weekly phone call log sheet was designed to cross verify the delivery of diabetes educational materials as well as to add any additional remarks mentioned by study participants. (PDF) [file pone.0241830.s016.pdf]

Annexure-9

Diabetes Management

Date	call#	Call Start Time	Call End Time	Diet	Exercise	Medication adherence	Eye problem	Kidney Disorders	Cardiac Diseases	Stroke	Skin diseases	Foot Ulcers	Periodic doctor visit	Blood sugar investigations (Fasting, PPBS and HbA1C	If any problems consult doctor

Patient ID:
