# Supplementary material for: Intervention through Short Messaging System (SMS) and phone call alerts reduced HbA1C levels in ~47% type-2 diabetics–results of a pilot study
Source: PLoS One. 2020 Nov 17;15(11):e0241830. doi: 10.1371/journal.pone.0241830 (PMC7671489; doi:10.1371/journal.pone.0241830)
Supplement: S17 File — The form was designed to capture information related to participants’ disease management practices every month after receiving weekly diabetes management education. (PDF) [file pone.0241830.s017.pdf]

Annexure-10

Data Sheet for capturing diabetes management practices of participants

Patient ID:

Madhumeha Nirvahane (Diabetes Management)													
Call #	Date	Call Duration		Dietary Habits	Physical Exercise		Medication Adherence	Glucose Tests		Eye & Foot care	Doctor's visits	Remarks
		Start time	End Time		Exercise	Duration		Type of test	Reading			
