# Supplementary material for: Intervention through Short Messaging System (SMS) and phone call alerts reduced HbA1C levels in ~47% type-2 diabetics–results of a pilot study
Source: PLoS One. 2020 Nov 17;15(11):e0241830. doi: 10.1371/journal.pone.0241830 (PMC7671489; doi:10.1371/journal.pone.0241830)
Supplement: S20 File — (ZIP) [file pone.0241830.s020.zip › Supporting information Tables R4 - Pdf/Tables R3 - Pdf/Table1.pdf]

Table1: Demographic Information of Study Participants		
**Demographics**	**N=380**	**% of total**
Male	209	55
Female	171	45
**Age**		
**Gender**	**Mean value**	
Male	55	
Female	54	
**Age Range**		
**Age Range (Years)**	**Number of individuals**	**% of total**
31-40	29	7.6
41-50	114	30
51-60	86	22.6
61-70	104	27.4
71-80	47	12.4
**Location**		
**Location**	**Number of individuals**	**% of total**
Mysuru	276	72.6
Mandya	76	20
Chamrajanagara	19	5
Hassan	9	2.3
**Domicile**		
**Domicile**	**Number of individuals**	**% of total**
Urban	85	22.3
Semi-urban	124	32.6
Rural	171	45
**Profession**		
**Profession**	**Number of individuals**	**% of total**
Agriculture	144	37.8
Office	28	7.3
Service industry	47	12.3
Homemaker	161	42.3
**Average Years of diabetes (Mean)**		
**Gender**	**Mean Value**	
Male	7.8	
Female	7.1	
**Diabetes associated comorbidities**		

**Comorbidities**	**Number of individuals**	**% of total**
Hypertension	133	35
Thyroid disorders	10	2.6
Joint pains	19	5
Retinopathy	19	5
Renal disorders	57	15
Diabetic foot	10	2.6
