# Supplementary material for: Intervention through Short Messaging System (SMS) and phone call alerts reduced HbA1C levels in ~47% type-2 diabetics–results of a pilot study
Source: PLoS One. 2020 Nov 17;15(11):e0241830. doi: 10.1371/journal.pone.0241830 (PMC7671489; doi:10.1371/journal.pone.0241830)
Supplement: S20 File — (ZIP) [file pone.0241830.s020.zip › Supporting information Tables R4 - Pdf/Tables R3 - Pdf/Table2.pdf]

Table 2: Participants' Disease Management Practices		
**Disease Management Practices**	**N=380**	**% of total**
**Frequency of doctor visit**		
**Period**	**Number of individuals**	**% of total**
1 month	114	30
3 months	247	65
6 months	19	5
More than 6 months	0	0
**Frequency of blood investigation**		
**Period**	**Number of individuals**	**% of total**
1 month	190	50
3 months	171	45
6 months	9	2.3
More than 6 months	10	2.6
**Type of blood investigations**		
**Investigation**	**Number of individuals**	**% of total**
Fasting	219	57.6
Post prandial blood sugar (PPBS)	142	37.3
Random	19	5
HbA1C	0	0
**Medication regimen**		
**Medication**	**Number of individuals**	**% of total**
Single oral hypoglycemic agents	162	42.6
Multiple oral hypoglycemic agents	85	22.3
Insulin	133	35
**Adherence to Dietary restrictions**		
**Adherence**	**Number of individuals**	**% of total**
Always	143	37.6
Sometimes	218	57.3
Never	19	5
**Time spent on physical activity**		
**Time**	**Number of individuals**	**% of total**
<1 hour/ day	304	80
1-2 hours/ day	47	12.3
Not everyday	29	7.6
**Frequency of foot inspection**		

Frequency	Number of individuals	% of total
Always	57	15
Sometimes	228	60
Never	95	25
**Frequency of eye inspection**		
**Frequency**	**Number of individuals**	**% of total**
Always	57	15
Sometimes	228	60
Never	95	25
**Awareness on self-management of diabetes**		
**Awareness**	**Number of individuals**	**% of total**
Yes	333	87.6
No	47	12.3
