# Supplementary material for: Intervention through Short Messaging System (SMS) and phone call alerts reduced HbA1C levels in ~47% type-2 diabetics–results of a pilot study
Source: PLoS One. 2020 Nov 17;15(11):e0241830. doi: 10.1371/journal.pone.0241830 (PMC7671489; doi:10.1371/journal.pone.0241830)
Supplement: S20 File — (ZIP) [file pone.0241830.s020.zip › Supporting information Tables R4 - Pdf/Tables R3 - Pdf/Table3.pdf]

Table3: Knowledge Assessment About Diabetes Management After Intervention		
**Knowledge Assessment**	**N=120**	**% of total**
**Symptoms of diabetes**		
**Symptoms**	**Number of individuals**	**% of total**
Excessive thirst	67	55.8
Hunger	30	25
Frequent urination	34	28.3
Weight loss	30	25
**Best practices of diabetes management**		
**Best Practices**	**Number of individuals**	**% of total**
Regular exercise	90	75
Controlled diet	94	78.3
Medication adherence	75	62.5
Periodic doctor visit	56	46.6
**Complications of Uncontrolled diabetes**		
**Complications**	**Number of individuals**	**% of total**
Heart disease	19	15.8
Eye problems	71	59.1
Stroke	21	17.5
Neurological diseases	22	18.3
Kidney diseases	30	25
Foot ulcers	49	40.8
**Symptoms of hypo-glycemia**		
**Symptoms**	**Number of individuals**	**% of total**
Dizziness	30	25
Hunger	26	21.6
Sweating	45	37.5
Shakiness	34	28.3
Anxiety	22	18.3
Moodiness	30	25
**Excellent glucose range**		
**Glucose value/ range**	**Number of individuals**	**% of total**
<80	0	0
80-120	90	75
120-180	30	25
>180	0	0

Investigation that gives accurate glucose reading		
**Investigations**	**Number of individuals**	**% of total**
HbA1c	113	94.1
PPBS	0	0
Fasting	7	5.8
