# Supplementary material for: Intervention through Short Messaging System (SMS) and phone call alerts reduced HbA1C levels in ~47% type-2 diabetics–results of a pilot study
Source: PLoS One. 2020 Nov 17;15(11):e0241830. doi: 10.1371/journal.pone.0241830 (PMC7671489; doi:10.1371/journal.pone.0241830)
Supplement: S20 File — (ZIP) [file pone.0241830.s020.zip › Supporting information Tables R4 - Pdf/Tables R3 - Pdf/Table4.pdf]

Table4: Patient Feedback on SMS & Phone Call Interventions		
**Feedback**	**N=120**	**% of total**
**Satisfaction on diabetes self-management orientation class**		
**Satisfaction**	**Number of individuals**	**% of total**
Satisfied	113	94.1
Unsatisfied	7	5.9
**Frequency of orientation classes required**		
**Frequency**	**Number of individuals**	**% of total**
1 month	11	9.1
3 months	94	78.3
6 months	15	12.5
**Comfortable with weekly SMS**		
**SMS**	**Number of individuals**	**% of total**
Yes	98	81.6
No	22	18.3
**Comfortable with monthly phone calls**		
**Phone calls**	**Number of individuals**	**% of total**
Yes	90	75
No	30	25
**Study helps in self-management of diabetes**		
**Satisfaction on current study**	**Number of individuals**	**% of total**
Yes	120	100
No	0	0
**Preferred mode of intervention**		
**Participants preference**	**Number of individuals**	**% of total**
SMS	22	18.3
Phone calls	98	81.6
**Reason for preferring phone calls (multiple options)**		
**Reasons**	**Number of individuals**	**% of total**
Feels good to talk than read	67	55.8
Interactive communication	97	80.8
Both	113	94.1
