# Supplementary material for: Intervention through Short Messaging System (SMS) and phone call alerts reduced HbA1C levels in ~47% type-2 diabetics–results of a pilot study
Source: PLoS One. 2020 Nov 17;15(11):e0241830. doi: 10.1371/journal.pone.0241830 (PMC7671489; doi:10.1371/journal.pone.0241830)
Supplement: S20 File — (ZIP) [file pone.0241830.s020.zip › Supporting information Tables R4 - Pdf/Tables R3 - Pdf/Table5.pdf]

Table5: Comparison of HbA1C values throughout the study						
**HbA1C Range**	**Baseline Data**		**Data at 8 Months**		**Data at 14 Months**	
	**Number of Participants**	**Baseline HbA1C Values**	**Number of Participants**	**HbA1C Values at 8 Months**	**Number of Participants**	**HbA1C Values at 14 Months**
<5	0	0	0	0	5	4.16±0.06
5.1-7	27	6.4±0.08	37	6.2±0.09	37	6.2±0.09
7.1-9	55	8.0±0.08	38	7.7±0.08	42	8.1±0.08
9.1-11	26	9.9±0.10	29	9.8±0.10	21	9.9±0.11
11.1-13	10	12.2±0.13	12	12.1±0.16	10	11.8±0.17
13.1-15	2	13.9±0.10	4	14.3±0.46	5	13.8±0.20
